# A Randomized, Placebo‐Controlled Trial of Hydroxychloroquine in Incomplete Lupus

**DOI:** 10.1002/art.43391

**Published:** 2025-12-12

**Authors:** Nancy J. Olsen, Duanping Liao, Judith A. James, Joel M. Guthridge, Cristina Arriens, Diane Kamen, Mariko Ishimori, Daniel J. Wallace, Christopher Striebich, Sonali Narain, Benjamin F. Chong, Fan He, Eric W. Schaefer, Vernon M. Chinchilli, David R. Karp

**Affiliations:** ^1^ Department of Medicine Penn State Milton S. Hershey Medical Center Hershey Pennsylvania; ^2^ Department of Public Health Sciences Penn State College of Medicine Hershey Pennsylvania; ^3^ Department of Arthritis and Clinical Immunology Oklahoma Medical Research Foundation Oklahoma City; ^4^ Department of Medicine Medical University of South Carolina Charleston; ^5^ Kao Autoimmunity Institute, Cedars Sinai Medical Center Los Angeles California; ^6^ Department of Medicine Anschutz Medical Center, University of Colorado Aurora; ^7^ Donald and Barbara Zucker School of Medicine at Hofstra/Northwell Health Great Neck New York; ^8^ Department of Medicine University of Texas Southwestern Medical Center Dallas

## Abstract

**Objective:**

Patients with features of systemic lupus erythematosus (SLE) who do not fulfill classification criteria can be designated as incomplete lupus erythematosus (ILE). This condition includes individuals with a high risk of progression to SLE. Treatment of ILE may reduce symptoms, severity, and incidence of SLE.

**Methods:**

Hydroxychloroquine (HCQ) was chosen as an ILE intervention for a randomized, double‐blind trial to determine whether the rate of accumulation of SLE features defined by the 2012 Systemic Lupus Erythematosus International Collaborating Clinics (SLICC) criteria could be reduced. ILE was defined as antinuclear antibody positivity with one to two additional criteria. Patients 15 to 49 years old were eligible. Randomization was 1:1 HCQ to placebo. Evaluations were at 3‐month intervals over 24 months. Meeting SLICC classification sooner required exit.

**Results:**

Participants (N = 187) were randomized at seven sites. After excluding 7 patients who met SLE classification at baseline when screening laboratory data were completed, 180 patients were analyzed: 92 receiving HCQ and 88 receiving placebo. Considering all these enrollees, 55 developed additional criteria. Of the 118 participants who exited early with SLE or who completed 24 months of evaluation, SLE classification developed in 24 (13.3%); another 24 developed additional criteria but did not meet classification. The rates of acquisition of SLICC criteria and progression to SLE were similar in the two groups (*P* = 0.72 and *P* = 0.98, respectively). Development of SLE was associated with new malar rash, oral ulcers, joint tenderness, or pleurisy (*P* < 0.04).

**Conclusion:**

Although the Study of Antimalarials in Incomplete Lupus Erythematosus (SMILE) did not show effects of HCQ on ILE progression, the results offer insights into SLE risk in the ILE population.

## INTRODUCTION

Systemic lupus erythematosus (SLE) is a complex, chronic autoimmune disease with significant prevalence in women less than 40 years of age. Despite advances in therapies, morbidity and mortality remain unacceptably high, especially given the young age of those afflicted.^1^ Early treatment is key to effective prevention of long‐term tissue damage. Even short‐term and intermittent lupus flares contribute to accumulation of irreversible damage.^2^, ^3^ Therefore, identification of early stages of SLE before development of organ damage is an important objective for improving SLE outcomes.

The concept of early identification of autoimmune disease has taken hold in many conditions over the past two decades. This has led to a description of disease progression starting from the presence of genetic risk and asymptomatic autoantibodies (stage 1) to evidence of early pathophysiology (stage 2) to incident disease meeting classification criteria (Stage 3).^4^ Implied in this description is the idea that stage 2 can be a modifiable state, in which pharmacological or nonpharmacological interventions will alter the development to Stage 3. As an example, a US Food and Drug Administration (FDA)–approved drug, teplizumab, is now available to delay the clinical onset of type I diabetes mellitus (DM) in persons with stage 2 disease as defined by high‐risk autoantibodies and dysglycemia.^5^ In rheumatoid arthritis (RA), intervention studies in stage 2 defined by autoantibodies and arthralgia or subclinical radiographic joint inflammation have been performed.^6^ Rituximab^7^ and abatacept^8^ were able to delay the progression to objective synovitis, whereas hydroxychloroquine (HCQ) was not.^6^ For SLE, the recognition that autoantibodies and cytokine mediators appear in serum years before a clinical diagnosis has led to interest in the feasibility of identifying a preclinical stage appropriate for therapeutic intervention to delay or prevent disease.^9^, ^10^

A major obstacle in the recognition of early SLE is the lack of useful biomarkers. The usual entry of a patient into medical care for a possible lupus diagnosis is antinuclear antibody (ANA) positivity. However this is a nonspecific test, and most individuals presenting with this scenario do not in fact have SLE. Those that are lupus suspects may have additional findings, such as the more specific autoantibodies to double‐stranded DNA (dsDNA) or Smith antigen.^11^ The presence of SLE‐associated serological autoimmunity in combination with immunopathology, such as leukopenia, hypocomplementemia, or inflammatory arthritis, would fit the concept of stage 2 SLE. The ANA‐positive patient with some additional SLE features that are insufficient to meet SLE classification criteria can be identified as having incomplete lupus erythematosus (ILE). Previous studies have documented that the ILE population includes patients who subsequently develop SLE or other classifiable autoimmune diseases.^12^, ^13^, ^14^, ^15^ Although exact prediction as to which ILE patients will develop SLE is not yet possible, most estimates suggest that 15% to 20% will progress within a few years.

Targeting the ILE population for early intervention has the potential to decrease or prevent severe SLE manifestations. In considering choices for an intervention, one key requirement was that it be safe and well tolerated. Furthermore, evidence that the candidate intervention modulates immune processes that contribute to disease would be necessary. HCQ fulfills these criteria as a safe medication in longstanding use that has been shown to modulate expression of immune abnormalities such as the type I interferon (IFN) gene expression signature that contributes to lupus pathogenesis.^16^, ^17^ Additional support was derived from the retrospective analysis of the US military cohort in which individuals with incomplete forms of lupus who were treated with HCQ had delayed onset of clinical disease.^18^

## PATIENTS AND METHODS

### Study design, patients, and treatment

The Study of Antimalarials in Incomplete Lupus Erythematosus (SMILE, NCT03030118) was a 24‐month, phase 2, randomized, double‐blind, placebo‐controlled multicenter study evaluating whether treatment with HCQ could slow accumulation of the 2012 Systemic Lupus Erythematosus International Collaborating Clinics (SLICC) criteria^19^ for the classification of SLE compared to placebo treatment in persons with ILE. Progression to classifiable SLE was not used as a primary outcome measure given that it would be less likely than simple criterion accumulation but was designated as a key secondary outcome. The final design was based on feedback from a mock recruitment exercise conducted as part of the prestudy planning grant, which confirmed feasibility.^20^

Enrollment was conducted at seven US sites: Penn State Milton S. Hershey Medical Center, University of Texas Southwestern Medical Center, Oklahoma Medical Research Foundation (OMRF), Medical University of South Carolina, Cedars‐Sinai Medical Center, University of Colorado Anschutz Medical Center, and Northwell Health. Eligible patients were 15 to 49 years of age and male or female with a positive ANA of 1:80 or greater by the standard immunofluorescence assay at a College of American Pathologists/Clinical Laboratory Improvement Amendments (CAP/CLIA)‐certified central laboratory (OMRF) and one or two additional items (laboratory and/or clinical) from the SLICC criteria. Participants were required to have no contraindication to use of HCQ, no previous treatment with HCQ or with immunosuppressive agents, and a normal baseline ophthalmologic examination, including visual fields and optical coherence tomography. Racial and ethnic background was self‐declared.

HCQ tablets (200 mg) were purchased from FDA‐approved generic manufacturers and sent to the University of Iowa Pharmaceuticals, Iowa City, Iowa, for manufacture of overencapsulated HCQ and matching placebo. Bottles of capsules were transferred to the Clinical Materials Services Unit of the University of Rochester for preparation of kits that were then sent to investigational pharmacies at each site. Patients who passed screening were randomized 1:1 to receive HCQ or placebo. Randomization was stratified by site and by number of SLICC criteria, either two or three, and was conducted using a centralized interactive system. Randomization was done with varying permutation block sizes at each investigational drug service pharmacy to minimize likelihood of inadvertent unblinding. Participants and clinical study staff at each site remained blinded throughout the trial. Pharmacists at each site knew which study group was assigned A or B but did not know the identity of each treatment. Dosing of HCQ was weight‐based: patients who were more than 40 kg received 400 mg whereas those weighing 40 kg or less received 200 mg. Blinded study medication was taken once daily for 96 weeks. Compliance was measured by pill counts at each visit. Participants with less than 80% compliance were counseled by site staff.

The protocol (Supplementary Clinical Research Protocol) was reviewed and approved by institutional review boards at each study site. All patients provided written, informed consent before any study‐related activities. Assent of minor participants and consent by the responsible adult was also obtained when appropriate. Use of HCQ in this study was exempt from Investigational New Drug regulations.

### Assessments

The primary endpoint was the rate of accumulation of SLICC criteria. Secondary endpoints included the proportion of patients who transitioned to SLE using the SLICC criteria or the 1997 revised American College of Rheumatology (ACR) criteria.^21^ Other measures conducted at study visits every three months included the modified Systemic Lupus Erythematosus Disease Activity Index 2 K (SLEDAI‐2 K), including the physician global assessment and the SLE flare index,^22^ the Cutaneous Lupus Erythematosus Disease Area and Severity Index (CLASI),^23^ and patient global visual analog scale. The Patient‐Reported Outcomes Measurement Information System 29 (PROMIS‐29) adult or pediatric survey supplemented with additional questions from the PROMIS Fatigue item bank was administered at visits 3 to 11 and included eight domains (physical function, anxiety, depression, fatigue, sleep disturbance, ability to participate in social activities, pain interference, pain intensity) and four composite scores created based on these domains (pain, emotional distress, physical health, mental health).^24^ The Connective Tissue Disease Screening Questionnaire (CSQ) was completed screening.^25^ The SLICC/ACR Damage Index^26^ was assessed at baseline, midpoint (week 52), and at the final visit (week 100).

Laboratory measures sent to local site laboratories included complete blood count, serum chemistries, urinalysis, and complement proteins 3 and 4. Additional tests at baseline were Coombs direct antiglobulin, urine pregnancy, glucose‐6‐phosphate dehydrogenase, and 25‐OH‐vitamin D. Coombs and urine pregnancy tests were repeated at week 52.

ANA measurements at screening were conducted at the CAP/CLIA‐certified Morris Reichlin Clinical Immunology Laboratory at OMRF using the Hep‐2 immunofluorescence assay. At baseline and all subsequent visits, an autoantibody profile was conducted at OMRF using the Bio‐Rad BioPlex 2200 for measurement of the following IgG specificities: anti‐dsDNA, Ro60, Ro52, La, Sm, Sm/nRNP, U1‐RNP, U1‐RNA A, Jo‐1, centromere, and histones. This anti‐dsDNA assay has 90% or greater specificity for SLE against relevant disease controls. Cardiolipin and beta‐2 glycoprotein I autoantibodies of the IgG and IgM specificities were also tested by BioPlex 2200.

Samples of serum, plasma, DNA, RNA from PAXGene (BD Biosciences) tubes, peripheral blood mononuclear cells, and urine obtained at each visit were banked in the OMRF CAP‐certified biorepository. All samples sent to OMRF were assigned a patient/sample ID and barcoded.

### Safety assessments

Adverse events (AEs) were recorded and classified by severity. Relatedness to study intervention and determination of whether the event was expected or unexpected was determined by each site's principal investigator. AE severity was determined using the National Cancer Institute's Common Terminology Criteria for Adverse Events (CTCAE). Serious AEs were reported to the data safety and monitoring board and to local institutional review boards per applicable regulations.

### Statistical analysis

The target sample size was 240 patients, with equal numbers in the placebo and HCQ groups, which was estimated based on pilot data and the report by Wieczorek et al^12^ to estimate the probability of progression in the SLICC count. A 25% withdrawal rate was anticipated, and the power was 90% to detect that the probability of progressing at least one stage was 0.4 for placebo and 0.2 for HCQ. For the secondary outcome variable, time to progression to classifiable SLE, a sample size of 192 patients was estimated to have 80% power to detect a difference in the probabilities of progression of 0.30 for placebo and 0.12 for HCQ.

For the primary outcome of SLICC accumulation over time, an ordinal logistic regression model was applied to compare the estimated slopes over time for the HCQ and placebo groups with respect to the SLICC criteria count over the 96‐week follow‐up period. The model fitted was conditional on the SLICC score from the previous time point (2 or 3) and therefore was only applied to nonbaseline time points. The statistical model included fixed effects for time (elapsed time since baseline), group (HCQ vs placebo), and an interaction between time and group. The interaction term indicated whether the slopes differed between groups over time and was the parameter of interest that was tested using a two‐sided test at the 0.05 significance level. We investigated using random effects for person to account for correlation among measurements over time, but the models would not converge, and therefore, the random effects were dropped. For the secondary analysis of progression from ILE to SLE, a Cox proportional hazards model was used to test differences between groups, and a Kaplan–Meier plot^27^ was constructed to visually assess curves.^28^

No interim analysis was planned or conducted. The data from the SMILE trial will be shared via ImmPort (www.immport.org).

## RESULTS

### Patients

A total of 187 patients were randomized at the seven sites (Supplementary Figure 1). The enrollment period was January 2018 through June 2022; the final patient visit was completed in July 2024. Enrollment was negatively impacted by the COVID‐19 pandemic, which, for a time, limited research activities. After the return of screening laboratory tests, seven participants were found to satisfy SLICC classification criteria at baseline. They did not complete additional visits and were removed from the analysis. This left 180 participants, 92 receiving HCQ and 88 receiving placebo (Table 1). The overall mean age was 33 years (range 15–49 years), and 91% of participants were female. Race and ethnicity categories show that most patients were White (74.4%) and were not Hispanic or Latino (82.2%). Approximately two‐thirds of enrollees had two SLICC criteria at baseline, and the remainder had three criteria. The most common pattern of SLICC criteria at baseline was one immunologic (ANA) and one clinical feature, which was present in 56.7% of those randomized.

**Table 1:** Demographics and key baseline characteristics by randomized group*

Variable	HCQ (n = 92)	Placebo (n = 88)	Total (N = 180)
Age, mean (SD), y	32.8 (9.8)	33.5 (8.6)	33.1 (9.2)
Biologic sex, n (%)			
Male	8 (8.7)	8 (9.1)	16 (8.9)
Female	84 (91.3)	80 (90.9)	164 (91.1)
Race, n (%)			
American Indian/Alaska Native	2 (2.2)	2 (2.3)	4 (2.2)
Asian	3 (3.3)	4 (4.5)	7 (3.9)
Black/African American	13 (14.1)	9 (10.2)	22 (12.2)
White	68 (73.9)	66 (75.0)	134 (74.4)
Multiple	3 (3.3)	4 (4.5)	7 (3.9)
Unknown/missing	3 (3.3)	3 (3.4)	6 (3.3)
Ethnicity, n (%)			
Hispanic or Latino	14 (15.2)	17 (19.3)	31 (17.2)
Not Hispanic or Latino	78 (84.8)	70 (79.5)	148 (82.2)
Unknown/missing	0 (0)	1 (1.1)	1 (0.6)
Number of SLICC criteria at randomization, n (%)			
2	62 (67.4)	56 (63.6)	118 (65.6)
3	30 (32.6)	32 (36.4)	62 (34.4)
Type of SLICC criteria at randomization, n (%)			
2 immunologic	6 (6.5)	10 (11.4)	16 (8.9)
1 immunologic +1 clinical	56 (60.9)	46 (52.3)	102 (56.7)
3 immunologic	2 (2.2)	2 (2.3)	4 (2.2)
2 immunologic +1 clinical	9 (9.8)	14 (15.9)	23 (12.8)
1 immunologic +2 clinical	19 (20.7)	16 (18.2)	35 (19.4)

* HCQ, hydroxychloroquine; SLICC, Systemic Lupus Erythematosus International Collaborating Clinics.

### SLICC criteria

Changes in SLICC criteria from baseline to the end of study or to early termination from study are shown in Tables 2 and 3. The protocol was completed in 50% of participants (47% receiving HCQ; 53% receiving placebo), and 24% had early termination per protocol (25% HCQ; 23% placebo). The termination group included 24 who achieved SLE classification with four or more SLICC criteria, including 12 (13.0%) in the HCQ group and 12 (13.6%) in the placebo group. Discontinuation due to participant withdrawal was 26% overall (28% HCQ; 24% placebo). Reasons for early termination included pregnancy (two patients in each arm), use of prohibited concomitant medications (5% overall), or loss to follow‐up and are detailed in Supplementary Table 1. No significant differences were seen in patient loss between the two treatment arms.

**Table 2:** Changes in SLICC criteria from baseline to end of study or early termination including all participants*

	HCQ n = 92	Placebo n = 88	Total N = 180
Change in SLICC criteria, n (%)			
2 to 2	42 (45.7)	36 (40.9)	78 (43.9)
2 to 3	15 (16.3)	16 (18.2)	31 (17.2)
2 to 4	5 (5.4)	4 (4.5)	9 (5.0)
3 to 3	22 (23.9)	25 (28.4)	47 (26.1)
3 to 4	8 (8.7)	7 (8)	15 (8.3)
Classification as SLE, n (%)			
No	79 (85.9)	77 (87.5)	156 (86.7)
Yes	13 (14.1)	11 (12.5)	24 (13.3)

* HCQ, hydroxychloroquine; SLE, systemic lupus erythematosus; SLICC, Systemic Lupus Erythematosus International Collaborating Clinics.

**Table 3:** Number and percentage of SMILE participants with each SLICC criterion at baseline by outcome*

	Nonprog, n = 70;	Prog, n = 24		Developed SLE, n = 24		Association of baseline variable with progression status^a^	
2012 SLICC classification criterion^b^	Baseline, n (%)	Baseline, n (%)	Last visit, n (%)	Baseline, n (%)	Last visit, n (%)	Nonprog, achieved SLE, and Prog, *P*	Nonprog and any Prog, *P*
Acute malar rash	16 (22.9)	3 (12.5)	5 (20.8)	7 (29.2)	11 (45.8)	0.399	1.00
Subacute cutaneous lupus	1 (1.4)	0	0	0	0		
Classic discoid lupus	2 (2.9)	0	0	0	0		
Nonscarring alopecia	11 (15.7)	4 (16.7)	7 (29.2)	3 (12.5)	7 (29.2)	0.999	1.00
Oral ulcers	3 (4.3)	1 (4.2)	4 (16.7)	2 (8.3)	7 (29.2)	0.840	0.686
Nasal ulcers	4 (5.7)	0	3 (12.5)	2 (8.3)	3 (12.5)	0.443	1.000
Synovitis with swelling or effusion	11 (15.7)	2 (8.3)	3 (12.5)	3 (12.5)	3 (12.5)	0.810	0.585
Joint tenderness	21 (30.0)	7 (29.2)	10 (41.7)	9 (37.5)	15 (62.5)	0.79	0.554
Pleuritis	2 (2.9)	1 (4.2)	1 (4.2)	2 (8.3)	5 (20.8)	0.568	0.395
Pericarditis	1 (1.4)	0	0	1 (4.2)	1 (4.2)	0.361	1.000
Leukopenia; <4000/mm^3^	8 (11.4)	3 (12.5)	7 (29.2)	0	7 (29.2)	0.22	0.521
Lymphopenia; <1000/mm^3^	5 (7.1)	0	0	0	2 (8.3)	0.212	0.079
Thrombocytopenia; <100,000/mm^3^	1 (1.4)	1 (4.2)	1 (4.2)	0	0	0.650	1.00
ANA	70 (100)	24 (100)	24 (100)	24 (100)	24 (100)		
Anti‐dsDNA	5 (7.1)	1 (4.2)	3 (12.5)	2 (8.3)	3 (12.5)	0.999	1.00
Anti‐Sm	1 (1.4)	0	0	1 (4.2)	1 (4.2)	0.65	1.00
Antiphospholipid	3 (4.3)	1 (4.2)	1 (4.2)	3 (12.5)	4 (16.7)	0.562	0.440
Low C3	5 (7.1)	0	5 (20.8)	3 (12.5)	5 (20.8)	0.199	1.00
Low C4	4 (5.7)	1 (4.2)	1 (4.2)	3 (12.5)	4 (16.7)	0.582	0.721
Direct Coombs Test	1 (1.4)	0	2 (8.3)	0	0	>0.999	1.000

* Analysis includes only 118 participants who completed 24 months of treatment or achieved SLE classification sooner. ANA, antinuclear antibody; dsDNA, double‐stranded DNA; nonprog, nonprogressor; prog, progressor; SLE, systemic lupus erythematosus; SLICC, Systemic Lupus Erythematosus International Collaborating Clinics; Sm, Smith; SMILE, Study of Antimalarials in Incomplete Lupus Erythematosus.

^a^ Fisher's exact test used to compare 3 groups and 2 groups.

^b^ Variables not present in any group are not shown.

**Figure 1:**
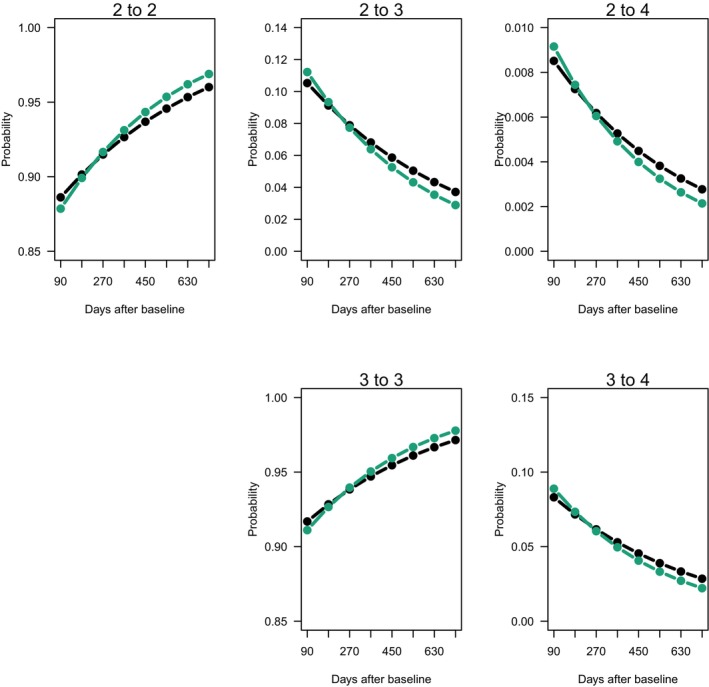
Progression of SLICC scores. Estimated probabilities for each possible transition of SLICC scores over time for HCQ (black) and placebo (green) groups. Transition probabilities are shown as a function of the previous score (2 for top row; 3 for bottom row), group, and time, using the fitted ordinal logistic regression model. Note that the y‐axis limits are different for each figure. HCQ, hydroxychloroquine; SLICC, Systemic Lupus Erythematosus International Collaborating Clinics.

The primary endpoint, rate of accumulation of SLICC criteria, showed similar odds of progressing to a higher SLICC score at each visit for each treatment arm (Figure 1). The odds ratio (OR) for the HCQ group was 0.85 and for the placebo group was 0.81, indicating that the groups had similar progression over time (*P* = 0.72). Two sensitivity analyses were done for the primary outcome: the first used visit labels instead of elapsed time, and the second relaxed the linearity assumption by allowing groups to be different at every time point (cell means model). Neither of these sensitivity analyses showed statistically significant differences between the two treatment groups (data not shown).

### Transition to SLE

**Figure 2:**
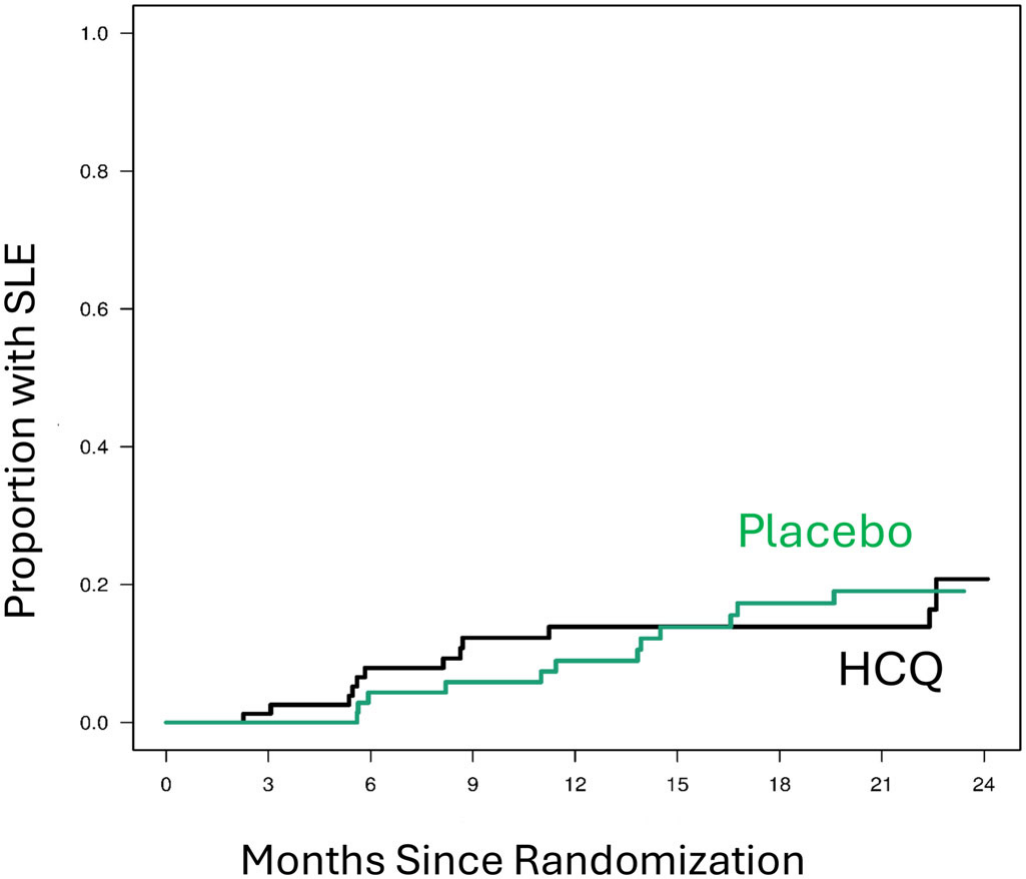
Kaplan–Meier curves for time to progression to SLE. HCQ, hydroxychloroquine; SLE, systemic lupus erythematosus.

A key secondary endpoint was transition to SLE, defined as achieving four or more SLICC criteria including at least one clinical and one immunologic criterion. A Cox proportional hazards regression analysis showed similar progression to SLE over time in the two groups with a hazard ratio of 1.01 (95% confidence interval [CI] 0.45–2.25; *P* = 0.98; Figure 2).

### Disease activity

Both treatment groups had significant decreases in the SLEDAI score over the study period. The HCQ group showed an increase in the proportion of low SLEDAI scores of 0 or 1, from 46.7% at baseline to 63.0% at visit 11. Similarly, the placebo increase in scores of 0 or 1 was from 51.1% to 72.0%. For every 90‐day increase in time, the HCQ group had higher odds of having a lower SLEDAI score (OR 1.12; 95% CI 1.03–1.21; *P* = 0.009), and in the placebo group, the odds were similar (OR 1.14; 95% CI 1.04–1.25; *P* = 0.004), with a nonsignificant difference between the two groups (*P* = 0.74).

### Patient reported outcomes (PROMIS)

For this secondary endpoint, each domain and composite score was analyzed for changes over time in the two treatment groups, and none of the slopes differed by group (not shown). A second analysis calculated the direct change in PROMIS T scores from baseline to the last recorded visit, and changes over time by group were compared using paired *t*‐tests. These also did not show significant changes in scores (not shown).

### Use of 2019 EULAR/ACR Classification Criteria for Systemic Lupus Erythematosus

The SMILE study used the 2012 SLICC criteria for the classification of lupus because those were the latest ones in existence at the time the trial was conceived and begun. Since then, newer criteria requiring the presence of a 1:80 ANA and using weighted values have been developed.^29^ In a post hoc analysis, the 2012 SLICC criteria were mapped to the 2019 EULAR/ACR criteria, and the appropriate weights were applied. Fever, which is not in the SLICC criteria, was not collected prospectively and could not be added to the equation. Using the 2019 EULAR/ACR criteria, 18 of 187 individuals randomized in SMILE would have been classified as having SLE at baseline with 10 or more points, including 4 of the 7 individuals classified as having SLE by SLICC criteria at baseline and excluded from the randomized trial, 3 of 24 individuals who later developed SLE by SLICC criteria, and 11 individuals who did not progress to SLE during SMILE. At the end of the SMILE trial, 6 of 32 participants who progressed from ANA and one SLICC criterion to ANA and two criteria would have been classified as SLE by the 2019 EULAR/ACR criteria. For the 24 participants who developed SLE according to SLICC criteria in SMILE, 12 of 12 who received placebo and 8 of 12 who received HCQ would have been classified as SLE by the 2019 criteria at their last visit.

### Predicting progression

The CSQ survey administered at screening has the potential to identify individuals at risk for development of SLE.^30^ In SMILE participants, SLE scores of 3 or greater, indicating possible SLE, were present in 64% of those receiving HCQ and 69% of those receiving placebo; scores of 4 or greater, considered higher risk of SLE, were present in 44% of both groups. Applying a logistic regression model to the pooled sample, a CSQ score of 3 or more was not significantly predictive of progression in number of SLICC criteria (OR 1.56; 95% CI 0.78–3.12; *P* = 0.21). Similarly, a CSQ score of 4 or more was not significantly predictive of SLICC progression (OR 1.25; 95% CI 0.66–2.35; *P* = 0.49).

SLICC classification criteria present at baseline were also examined for relation to progression, either addition of more criteria or achievement of SLE classification.^3^ In these analyses, progression or development of SLE did not show correlation with any baseline variables (Supplementary Table 1). The only one that even showed a trend toward significance, lymphopenia (*P* = 0.079), was actually in the opposite direction: 7% of nonprogressors had this feature at baseline, whereas it was present in none of the progressors. This result differs from at least one other study that showed a correlation between lymphopenia and progression of SLE features.^14^

SLICC criteria that developed during the trial in progressors, including those who developed SLE, revealed four variables that were significantly different from baseline: oral ulcers (*P* = 0.0031), joint tenderness (*P* = 0.0211), leukopenia (*P* = 0.0286), and low C3 (*P* = 0.0464) (Table 4). When this analysis was done for those who developed SLE compared to nonprogressors, four variables were significantly different: malar rash (*P* = 0.0394), oral ulcers (*P* = 0.0023), joint tenderness (*P* = 0.0071), and pleurisy (*P* = 0.0110). These findings suggest that ILE patients with these features may merit close observation and follow‐up.

**Table 4:** Number and percentage of SMILE participants with each SLICC criterion at final visit by outcome*

2012 SLICC classification criterion^b^	Nonprogressor N = 70, n (%)	Progressor and developed SLE n = 48, n (%)	*P* value	Developed SLE n = 24, n (%)	*P* value^a^
Malar rash	16 (22.9)	16 (33.3)	NS	11 (45.8)	**0.0394**
Nonscarring alopecia	11 (15.7)	14 (29.2)	NS	7 (29.2)	NS
Oral ulcers	3 (4.3)	11 (2.9)	**0.0031**	7 (29.2)	**0.0023**
Nasal ulcers	4 (5.7)	6 (12.5)	NS	3 (12.5)	NS
Synovitis on examination	11 (15.7)	6 (12.5)	NS	3 (12.5)	NS
Joint tenderness	21 (30.0)	25 (52.1)	**0.0211**	15 (62.5)	**0.0071**
Pleurisy	2 (2.9)	6 (12.5)	NS	5 (20.8)	**0.0110**
Pericarditis	1 (1.4)	1 (2.1)	NS	1 (4.2)	NS
Leukopenia	8 (11.4)	14 (29.2)	**0.0286**	7 (29.2)	0.0545
Lymphopenia	5 (7.1)	2 (4.2)	NS	2 (8.3)	NS
Thrombocytopenia	1 (1.4)	1 (2.1)	NS	0	NS
Anti‐DNA	5 (7.1)	6 (12.5)	NS	3 (12.5)	NS
Anti‐Smith	1 (1.4)	1 (2.1)	NS	1 (4.2)	NS
Antiphospholipid	3 (4.3)	10 (20.8)	NS	4 (16.7)	NS
Low C3	5 (7.1)	5 (10.4)	0.51	5 (20.8)	NS
Low C4	4 (5.7)	4 (8.3)	0.4830	4 (16.7)	NS

* Analysis includes only 118 participants who completed 24 months of treatment or achieved SLE classification sooner. Bolded values indicate significance. NS, not significant; SLE, systemic lupus erythematosus; SLICC, Systemic Lupus Erythematosus International Collaborating Clinics; SMILE, Study of Antimalarials in Incomplete Lupus Erythematosus.

^a^ Fisher's exact test used.

^b^ Variables not present in any group are not shown.

### Safety

HCQ has a long record of safe use in patients with SLE, and this finding was also observed in SMILE participants. A total of 20 severe AEs were reported in 16 participants, and these were equally split, with 10 in each of the treatment groups. None of the serious adverse events in the HCQ group were judged as related to use of the drug. Only two AEs were considered probably related to the study intervention (diarrhea and migraine), but neither of these were severe; both were in the HCQ group. Five participants failed the screening ophthalmologic examinations for abnormalities that were sufficient to raise concerns about the ability to monitor investigational treatment. No abnormal ophthalmologic findings were reported at the final posttreatment visit.

## DISCUSSION

Major advances have been made in the treatment of SLE over the past two decades. These include targeted therapies, deeper scientific understanding of the underlying pathophysiology, and development of personalized approaches to the heterogeneous manifestations. Survival rates have improved for some, but mortality rates remain unacceptably high.^31^ One approach that has potential to improve outcomes is early diagnosis and treatment before organ damage has become manifest. The recognition that autoantibodies and other mediators are present years before SLE diagnosis made feasible the possibility of finding patients to treat with interventions that would slow, ameliorate, or even prevent disease.^10^, ^32^ Because such individuals would not have organ threatening SLE features at the time this early stage was detected, any intervention would have to be well tolerated and have an established safety profile. Furthermore, a population with a well‐defined risk would need to be identified. The proportion of individuals who show an autoimmunity profile but never develop SLE is not known. SLE and RA are, in this way, different than type I DM, in which a combination of immune and endocrine markers identifies individuals who will with near certainty develop disease.

The SMILE study used criteria validated for classification of SLE to identify patients with incomplete disease because no classification criteria for ILE have been developed. Whether these elements of SLE in fact identify individuals who are at risk for disease progression is unknown, but this was the only reasonable tool available when the study was initiated. The SMILE results do indicate that at least a subset of the ILE individuals accumulated additional SLICC criteria, and approximately 13% progressed to SLE classification within the 24‐month treatment period. This is close to the prestudy estimate of 15% and compatible with results of other observational studies.^14^

The choice of HCQ as intervention was based on retrospective data indicating that it delayed the development of SLE in patients who had features similar to those treated in SMILE.^18^ Furthermore, HCQ has been shown to quench the type I IFN gene expression signature in ILE. The type I IFN pathway is considered a major contributor to pathogenesis of SLE, and blockade of the IFN receptor has therapeutic benefit.^33^ ILE patients expressing this signature have higher levels of autoantibodies.^34^ It was reasonable to consider that such an intervention could delay accumulation of SLICC criteria.

Two other studies of HCQ in preclinical autoimmunity have also had negative results. In the StopRA trial (NCT02603146), individuals with antibodies to citrullinated proteins were treated with HCQ in a placebo‐controlled, blinded design. After 36 months, clinically apparent RA developed in 29.0% of the HCQ group and in 32.9% of the placebo group (*P* = 0.522). Other attempts at preclinical RA treatment include a trial with abatacept, which did show a statistically significant difference in development of RA.^8^ Rituximab also was shown to delay onset of RA compared to placebo‐treated patients.^7^

The other HCQ trial was in individuals at risk for development of type I DM (NCT03428945).^35^ HCQ did not prevent progression to abnormal glucose tolerance or clinical diabetes, and the trial was stopped because of futility.

A recently reported observational study showed that initiation of HCQ in patients with cutaneous LE prevented progression to SLE.^36^ Progression was seen in 4.8% of 186 patients treated with HCQ versus 27% of 100 patients who received only topical agents (*P* < 0.001). A caveat was that this study was unblinded and not randomized, and the HCQ group had significantly more severe skin disease, a known risk for progressive illness. Although it might appear paradoxical that these were the patients who had less progression, the results may indicate that patients who have activated disease pathways present more suitable targets for HCQ action.

The predominance in SMILE of White patients over age 30 favors development of a milder form of SLE.^37^ It follows, therefore, that a limitation of SMILE was the underenrollment of Hispanic and Black participants, groups that have higher risk of poor SLE outcomes.^38^, ^39^ It may be that SLE onset is more rapid in these individuals, thus making an ILE phase less apparent. Existing ILE registries at two SMILE sites, Hershey and Oklahoma City, had a higher proportion of Hispanic and Black enrollees than in the trial, suggesting hesitancy or barriers to joining an interventional research study.^16^, ^40^

Other studies examining lupus progression have noted that development of systemic disease in patients presenting with only cutaneous or incomplete features resulted in mild SLE manifestations.^12^, ^14^ So, whether an intervention to prevent progression of what is likely to be milder disease would show significant long‐term benefits remains to be proven.

A further limitation of SMILE was that measurement of HCQ levels was not built into the protocol, and funding for such determinations was not available. The possibility that nonresponse to HCQ may have been related to subtherapeutic dosing cannot be excluded without these additional measurements, which might be possible in the future on stored samples.

The SMILE data indicate that the SLICC criteria do identify individuals who progress to SLE, but their sensitivity and specificity is not known. Moreover, evaluating changes in skin, mucous membranes, or joints is subjective, and there was inescapable ascertainment bias, given that these were all individuals being closely observed for new features. The relatively high proportion of reported acute cutaneous lupus, for example, might indicate that other etiologies for rash, such as rosacea, were not always able to be excluded. Progression was associated with some of the more quantitative outcomes including complement levels and leukopenia, as well increased pleurisy symptoms. Some or all of these may be useful in clinical practice. Whether combinations of these features might be more predictive of progression will be of interest for future studies. An additional twist is that the SLICC criteria count toward the overall score if ever present, even if those values normalize during the course of ILE. Whether some patients in SMILE in fact normalized some criteria and whether or not such changes were related to use of HCQ would be of interest. Post hoc analyses suggested that the EULAR/ACR 2019 criteria differs from the SLICC criteria in terms of classification of ILE, and its application may be useful in future studies of incomplete syndromes.

Undifferentiated patients might transition to classifiable conditions other than SLE. No such transitions were reported in SMILE patients, although one participant who withdrew was later found to have multiple sclerosis. In a previous observational study using a similar population, diagnoses including ankylosing spondylitis and malignancies developed in <5% of participants, and others developed classifiable Sjögren disease.^14^ Long‐term follow‐up of SMILE patients for development of SLE or other conditions will be of interest.

## AUTHOR CONTRIBUTIONS

All authors contributed to at least one of the following manuscript preparation roles: conceptualization AND/OR methodology, software, investigation, formal analysis, data curation, visualization, and validation AND drafting or reviewing/editing the final draft. As corresponding author, Dr Olsen confirms that all authors have provided the final approval of the version to be published and takes responsibility for the affirmations regarding article submission (eg, not under consideration by another journal), the integrity of the data presented, and the statements regarding compliance with institutional review board/Declaration of Helsinki requirements.

## Supporting information


**Disclosure Form**:

**Data S1:** Supporting Information

Figure S1:

**Table S1:** Final Study Termination Status
