# Supplementary material for: A Randomized, Placebo‐Controlled Trial of Hydroxychloroquine in Incomplete Lupus
Source: Arthritis Rheumatol. 2025 Dec 12;78(4):870–9. doi: 10.1002/art.43391 (PMC13054461; doi:10.1002/art.43391)
Supplement: Supplementary file 1 — Disclosure Form: [file ART-78-870-s001.pdf]

ICMJE DISCLOSURE FORM

Date: 8/28/2025

Your Name: Nancy J. Olsen

Manuscript Title: A randomized, placebo-controlled trial of hydroxychloroquine in incomplete lupus

Manuscript Number (if known): Ar-25-0931.R1

In the interest of transparency, we ask you to disclose all relationships/activities/interests listed below that are related to the content of your manuscript. "Related" means any relation with for-profit or not-for-profit third parties whose interests may be affected by the content of the manuscript. Disclosure represents a commitment to transparency and does not necessarily indicate a bias. If you are in doubt about whether to list a relationship/activity/interest, it is preferable that you do so.

The author's relationships/activities/interests should be defined broadly. For example, if your manuscript pertains to the epidemiology of hypertension, you should declare all relationships with manufacturers of antihypertensive medication, even if that medication is not mentioned in the manuscript.

In item #1 below, report all support for the work reported in this manuscript without time limit. For all other items, the time frame for disclosure is the past 36 months.

		**Name all entities with whom you have this relationship or indicate none (add rows as needed)**	**Specifications/Comments (e.g., if payments were made to you or to your institution)**
		Time frame: Since the initial planning of the work	
1	All support for the present manuscript (e.g., funding, provision of study materials, medical writing, article processing charges, etc.) **No time limit for this item.**	☐	
		NIH U01AR071077	All payments to institution.
		NIH U34AR06739201	
		U54 TR002014-05A1	Click the tab key to add additional rows.
		Time frame: past 36 months	
2	Grants or contracts from any entity (if not indicated in item #1 above).	☐ **None**	
		Zenas	Penn State
		SUNY – Syracuse	Penn State
		NYU	Penn State
		Benaroya Research Institute	Penn State

		Name all entities with whom you have this relationship or indicate none (add rows as needed)	Specifications/Comments (e.g., if payments were made to you or to your institution)
**3**	Royalties or licenses	☒ **None**	
**4**	Consulting fees	☒ **None**	
**5**	Payment or honoraria for lectures, presentations, speakers bureaus, manuscript writing or educational events	☐ **None**	
		NYU Langone Medical Center	Olsen
**6**	Payment for expert testimony	☒ **None**	
**7**	Support for attending meetings and/or travel	☒ **None**	
**8**	Patents planned, issued or pending	☒ **None**	
**9**	Participation on a Data Safety Monitoring Board or Advisory Board	☐ **None**	
		GSK – DSMB , Olsen	Olsen
		NIH – DSMB, Olsen, U01AR080985	Olsen,
**10**	Leadership or fiduciary role in	☒ **None**	

		Name all entities with whom you have this relationship or indicate none (add rows as needed)	Specifications/Comments (e.g., if payments were made to you or to your institution)
	other board, society, committee or advocacy group, paid or unpaid		
**11**	Stock or stock options	☒ **None**	
**12**	Receipt of equipment, materials, drugs, medical writing, gifts or other services	☒ **None**	
**13**	Other financial or non-financial interests	☒ **None**	

Please place an "X" next to the following statement to indicate your agreement:

☒ I certify that I have answered every question and have not altered the wording of any of the questions on this form.

ICMJE DISCLOSURE FORM

Date:________________08/30/2025____
Your Name:________Duanping Liao
Manuscript Title:___A Randomized, placebo-controlled trial of hydroxychloroquine in incomplete lupus_____________
Manuscript number (if known):___________________ar-25-0931.R1____

In the interest of transparency, we ask you to disclose all relationships/activities/interests listed below that are related to the content of your manuscript. "Related" means any relation with for-profit or not-for-profit third parties whose interests may be affected by the content of the manuscript. Disclosure represents a commitment to transparency and does not necessarily indicate a bias. If you are in doubt about whether to list a relationship/activity/interest, it is preferable that you do so.

The following questions apply to the author's relationships/activities/interests as they relate to the current manuscript only.

The author's relationships/activities/interests should be defined broadly. For example, if your manuscript pertains to the epidemiology of hypertension, you should declare all relationships with manufacturers of antihypertensive medication, even if that medication is not mentioned in the manuscript.

In item #1 below, report all support for the work reported in this manuscript without time limit. For all other items, the time frame for disclosure is the past 36 months.

		**Name all entities with whom you have this relationship or indicate none (add rows as needed)**	**Specifications/Comments (e.g., if payments were made to you or to your institution)**
		**Time frame: Since the initial planning of the work**	
1	All support for the present manuscript (e.g., funding, provision of study materials, medical writing, article processing charges, etc.) **No time limit for this item.**	____	
		U01AR071077	institution
		U34AR06739201	institution
		**Time frame: past 36 months**	
2	Grants or contracts from any entity (if not indicated in item #1 above).	__X__ None	
3	Royalties or licenses	__X__ None	

4	Consulting fees	__X__ None	
5	Payment or honoraria for lectures, presentations, speakers bureaus, manuscript writing or educational events	__X__ None	
6	Payment for expert testimony	__X__ None	
7	Support for attending meetings and/or travel	__X__ None	
8	Patents planned, issued or pending	__X__ None	
9	Participation on a Data Safety Monitoring Board or Advisory Board	__X__ None	
10	Leadership or fiduciary role in other board, society, committee or advocacy group, paid or unpaid	__X__ None	
11	Stock or stock options	__X__ None	
12	Receipt of equipment, materials, drugs, medical writing, gifts or other services	__X__ None	
13	Other financial or non-financial interests	__X__ None	

Please place an "X" next to the following statement to indicate your agreement:

__X_ I certify that I have answered every question and have not altered the wording of any of the questions on this form.

ICMJE DISCLOSURE FORM

Date: 8/28/2025

Your Name: Judith James

Manuscript Title: A randomized, placebo-controlled trial of hydroxychloroquine in incomplete lupus

Manuscript Number (if known): Ar-25-0931

In the interest of transparency, we ask you to disclose all relationships/activities/interests listed below that are related to the content of your manuscript. "Related" means any relation with for-profit or not-for-profit third parties whose interests may be affected by the content of the manuscript. Disclosure represents a commitment to transparency and does not necessarily indicate a bias. If you are in doubt about whether to list a relationship/activity/interest, it is preferable that you do so.

The author's relationships/activities/interests should be defined broadly. For example, if your manuscript pertains to the epidemiology of hypertension, you should declare all relationships with manufacturers of antihypertensive medication, even if that medication is not mentioned in the manuscript.

In item #1 below, report all support for the work reported in this manuscript without time limit. For all other items, the time frame for disclosure is the past 36 months.

		Name all entities with whom you have this relationship or indicate none (add rows as needed)	Specifications/Comments (e.g., if payments were made to you or to your institution)
		**Time frame: Since the initial planning of the work**	
1	All support for the present manuscript (e.g., funding, provision of study materials, medical writing, article processing charges, etc.) **No time limit for this item.**	☐	
		NIH U01AR071077	All payments to institutions.
		NIH U34AR06739201	
			Click the tab key to add additional rows.
		UM1AI44292	
		U01AI76244	
		U54GM104938	
		P30AR073750	
		**Time frame: past 36 months**	
2	Grants or contracts from any entity (if not indicated in item #1 above).	☒ **None**	

		Name all entities with whom you have this relationship or indicate none (add rows as needed)	Specifications/Comments (e.g., if payments were made to you or to your institution)
**3**	Royalties or licenses	☒ **None**	
**4**	Consulting fees	☐ **None**	
		GSK	James
**5**	Payment or honoraria for lectures, presentations, speakers bureaus, manuscript writing or educational events	☒ **None**	
**6**	Payment for expert testimony	☒ **None**	
**7**	Support for attending meetings and/or travel	☒ **None**	
**8**	Patents planned, issued or pending	☒ **None**	
**9**	Participation on a Data Safety Monitoring Board or Advisory Board	☒ **None**	

		Name all entities with whom you have this relationship or indicate none (add rows as needed)	Specifications/Comments (e.g., if payments were made to you or to your institution)
**10**	Leadership or fiduciary role in other board, society, committee or advocacy group, paid or unpaid	☒ **None**	
**11**	Stock or stock options	☒ **None**	
**12**	Receipt of equipment, materials, drugs, medical writing, gifts or other services	☒ **None**	
**13**	Other financial or non-financial interests	☒ **None**	

Please place an "X" next to the following statement to indicate your agreement:

☒ I certify that I have answered every question and have not altered the wording of any of the questions on this form.

ICMJE DISCLOSURE FORM

**Date:**	8/28/2025
**Your Name:**	Joel Guthridge
**Manuscript Title:**	A randomized, placebo-controlled trial of hydroxychloroquine in incomplete lupus
**Manuscript Number (if known):**	Ar-25-0931

In the interest of transparency, we ask you to disclose all relationships/activities/interests listed below that are related to the content of your manuscript. "Related" means any relation with for-profit or not-for-profit third parties whose interests may be affected by the content of the manuscript. Disclosure represents a commitment to transparency and does not necessarily indicate a bias. If you are in doubt about whether to list a relationship/activity/interest, it is preferable that you do so.

The author's relationships/activities/interests should be defined broadly. For example, if your manuscript pertains to the epidemiology of hypertension, you should declare all relationships with manufacturers of antihypertensive medication, even if that medication is not mentioned in the manuscript.

In item #1 below, report all support for the work reported in this manuscript without time limit. For all other items, the time frame for disclosure is the past 36 months.

		**Name all entities with whom you have this relationship or indicate none (add rows as needed)**	**Specifications/Comments (e.g., if payments were made to you or to your institution)**
		Time frame: Since the initial planning of the work	
**1**	All support for the present manuscript (e.g., funding, provision of study materials, medical writing, article processing charges, etc.) **No time limit for this item.**	☐	
		NIH U01AR071077	All payments to institutions.
		NIH U34AR06739201	
			Click the tab key to add additional rows.
		UM1AI44292	
		U01AI76244	
		U54GM104938	
		P30AR073750	
		Time frame: past 36 months	
**2**	Grants or contracts from any entity (if not indicated in item #1 above).	☒ **None**	

		Name all entities with whom you have this relationship or indicate none (add rows as needed)	Specifications/Comments (e.g., if payments were made to you or to your institution)
**3**	Royalties or licenses	☒ **None**	
**4**	Consulting fees	☒ **None**	
			James
**5**	Payment or honoraria for lectures, presentations, speakers bureaus, manuscript writing or educational events	☒ **None**	
**6**	Payment for expert testimony	☒ **None**	
**7**	Support for attending meetings and/or travel	☒ **None**	
**8**	Patents planned, issued or pending	☒ **None**	
**9**	Participation on a Data Safety Monitoring Board or Advisory Board	☒ **None**	

		Name all entities with whom you have this relationship or indicate none (add rows as needed)	Specifications/Comments (e.g., if payments were made to you or to your institution)
**10**	Leadership or fiduciary role in other board, society, committee or advocacy group, paid or unpaid	☒ **None**	
**11**	Stock or stock options	☒ **None**	
**12**	Receipt of equipment, materials, drugs, medical writing, gifts or other services	☒ **None**	
**13**	Other financial or non-financial interests	☒ **None**	

Please place an "X" next to the following statement to indicate your agreement:

☒ I certify that I have answered every question and have not altered the wording of any of the questions on this form.

ICMJE DISCLOSURE FORM

Date:________________08/30/2025____
Your Name:________Cristina Arriens____
Manuscript Title:___A Randomized, placebo-controlled trial of hydroxychloroquine in incomplete lupus_____________
Manuscript number (if known):___________________ar-25-0931.R1____

In the interest of transparency, we ask you to disclose all relationships/activities/interests listed below that are related to the content of your manuscript. "Related" means any relation with for-profit or not-for-profit third parties whose interests may be affected by the content of the manuscript. Disclosure represents a commitment to transparency and does not necessarily indicate a bias. If you are in doubt about whether to list a relationship/activity/interest, it is preferable that you do so.

The following questions apply to the author's relationships/activities/interests as they relate to the current manuscript only.

The author's relationships/activities/interests should be defined broadly. For example, if your manuscript pertains to the epidemiology of hypertension, you should declare all relationships with manufacturers of antihypertensive medication, even if that medication is not mentioned in the manuscript.

In item #1 below, report all support for the work reported in this manuscript without time limit. For all other items, the time frame for disclosure is the past 36 months.

		**Name all entities with whom you have this relationship or indicate none (add rows as needed)**	**Specifications/Comments (e.g., if payments were made to you or to your institution)**
		**Time frame: Since the initial planning of the work**	
1	All support for the present manuscript (e.g., funding, provision of study materials, medical writing, article processing charges, etc.) **No time limit for this item.**	____	
		U01AR071077	institution
		U34AR06739201	institution
		**Time frame: past 36 months**	
2	Grants or contracts from any entity (if not indicated in item #1 above).	____	
		AstraZeneca	institution
		Bristol Myers Squibb	institution
3	Royalties or licenses	__X__ None	

4	Consulting fees	__X__ None	
5	Payment or honoraria for lectures, presentations, speakers bureaus, manuscript writing or educational events	____	
		Aurinia	self
		AstraZeneca	self
6	Payment for expert testimony	__X__ None	
7	Support for attending meetings and/or travel	____<br>AstraZeneca	self
		Bristol Myers Squibb	self
		Kezar	self
		UCB	self
8	Patents planned, issued or pending	__X__ None	
9	Participation on a Data Safety Monitoring Board or Advisory Board	____<br>AstraZeneca	self
		Aurinia	self
		Bristol Myers Squibb	self
		Cabaletta	self
		Health and Wellness Partners	self
		Kezar	self
		Synthekine	self
		UCB	self
10	Leadership or fiduciary role in other board, society, committee or advocacy group, paid or unpaid	_X___ None	
11	Stock or stock options	__X__ None	
12	Receipt of equipment, materials, drugs, medical writing, gifts or other services	____<br>AstraZeneca	institution
		Aurinia	institution
		Bristol Myers Squibb	institution
13	Other financial or non-financial interests	__X__ None	

Please place an "X" next to the following statement to indicate your agreement:

__X_ I certify that I have answered every question and have not altered the wording of any of the questions on this form.

ICMJE DISCLOSURE FORM

**Date:**	8/30/2025
**Your Name:**	Diane Kamen
**Manuscript Title:**	A randomized, placebo-controlled trial of hydroxychloroquine in incomplete lupus
**Manuscript Number (if known):**	Ar-25-0931

In the interest of transparency, we ask you to disclose all relationships/activities/interests listed below that are related to the content of your manuscript. "Related" means any relation with for-profit or not-for-profit third parties whose interests may be affected by the content of the manuscript. Disclosure represents a commitment to transparency and does not necessarily indicate a bias. If you are in doubt about whether to list a relationship/activity/interest, it is preferable that you do so.

The author's relationships/activities/interests should be defined broadly. For example, if your manuscript pertains to the epidemiology of hypertension, you should declare all relationships with manufacturers of antihypertensive medication, even if that medication is not mentioned in the manuscript.

In item #1 below, report all support for the work reported in this manuscript without time limit. For all other items, the time frame for disclosure is the past 36 months.

		**Name all entities with whom you have this relationship or indicate none (add rows as needed)**	**Specifications/Comments (e.g., if payments were made to you or to your institution)**
		**Time frame: Since the initial planning of the work**	
1	All support for the present manuscript (e.g., funding, provision of study materials, medical writing, article processing charges, etc.) **No time limit for this item.**	☐	
		NIH U01AR071077	All payments to institution.
			Click the tab key to add additional rows.
		**Time frame: past 36 months**	
2	Grants or contracts from any entity (if not indicated in item #1 above).	☐ **None**	
		NIAID co-funded with LFA/Phase II Controlled Trial of Allogeneic MSCs for the Treatment of Refractory Lupus - MPI	All payments to institution.
		NIAID/Mechanisms of New-Onset Autoimmunity/Longitudinal Immune Systems Analysis (MONA-LISA) - Site PI	All payments to institution.
		NIAID/ITN Trial of VIB4920 for Active Lupus Nephritis (VIBRANT) - Site PI	All payments to institution.

		Name all entities with whom you have this relationship or indicate none (add rows as needed)	Specifications/Comments (e.g., if payments were made to you or to your institution)
**3**	Royalties or licenses	☒ **None**	
**4**	Consulting fees	☒ **None**	
**5**	Payment or honoraria for lectures, presentations, speakers bureaus, manuscript writing or educational events	☒ **None**	
**6**	Payment for expert testimony	☒ **None**	
**7**	Support for attending meetings and/or travel	☒ **None**	
**8**	Patents planned, issued or pending	☒ **None**	
**9**	Participation on a Data Safety Monitoring Board or Advisory Board	☒ **None**	
**10**	Leadership or fiduciary role in other board, society,	☒ **None**	

		Name all entities with whom you have this relationship or indicate none (add rows as needed)	Specifications/Comments (e.g., if payments were made to you or to your institution)
	committee or advocacy group, paid or unpaid		
**11**	Stock or stock options	☒ **None**	
**12**	Receipt of equipment, materials, drugs, medical writing, gifts or other services	☒ **None**	
**13**	Other financial or non-financial interests	☒ **None**	

Please place an "X" next to the following statement to indicate your agreement:

☒ I certify that I have answered every question and have not altered the wording of any of the questions on this form.

ICMJE DISCLOSURE FORM

Date:________________08/30/2025____
Your Name:________Mariko Ishimori____
Manuscript Title:___A Randomized, placebo-controlled trial of hydroxychloroquine in incomplete lupus_____________
Manuscript number (if known):___________________ar-25-0931.R1____

In the interest of transparency, we ask you to disclose all relationships/activities/interests listed below that are related to the content of your manuscript. "Related" means any relation with for-profit or not-for-profit third parties whose interests may be affected by the content of the manuscript. Disclosure represents a commitment to transparency and does not necessarily indicate a bias. If you are in doubt about whether to list a relationship/activity/interest, it is preferable that you do so.

The following questions apply to the author's relationships/activities/interests as they relate to the <u>current manuscript only</u>.

The author's relationships/activities/interests should be <u>defined broadly</u>. For example, if your manuscript pertains to the epidemiology of hypertension, you should declare all relationships with manufacturers of antihypertensive medication, even if that medication is not mentioned in the manuscript.

In item #1 below, report all support for the work reported in this manuscript without time limit. For all other items, the time frame for disclosure is the past 36 months.

		**Name all entities with whom you have this relationship or indicate none (add rows as needed)**	**Specifications/Comments (e.g., if payments were made to you or to your institution)**
		**Time frame: Since the initial planning of the work**	
1	All support for the present manuscript (e.g., funding, provision of study materials, medical writing, article processing charges, etc.) **No time limit for this item.**	____ UL1TR001881	CSMC
		U01AR071077	CSMC
		U34AR06739201	CSMC
		**Time frame: past 36 months**	
2	Grants or contracts from any entity (if not indicated in item #1 above).	_X___ None	
3	Royalties or licenses	__X__ None	

4	Consulting fees	__X__ None	
5	Payment or honoraria for lectures, presentations, speakers bureaus, manuscript writing or educational events	__X__ None	
6	Payment for expert testimony	__X__ None	
7	Support for attending meetings and/or travel	__X__ None	
8	Patents planned, issued or pending	__X__ None	
9	Participation on a Data Safety Monitoring Board or Advisory Board	__X__ None	
10	Leadership or fiduciary role in other board, society, committee or advocacy group, paid or unpaid	_X___ None	
11	Stock or stock options	__X__ None	
12	Receipt of equipment, materials, drugs, medical writing, gifts or other services	__X__ None	
13	Other financial or non-financial interests	_X___ None	

Please place an "X" next to the following statement to indicate your agreement:

__X_ I certify that I have answered every question and have not altered the wording of any of the questions on this form.

ICMJE DISCLOSURE FORM

Date:________________08/30/2025____
Your Name:________Daniel J Wallace
Manuscript Title:___A Randomized, placebo-controlled trial of hydroxychloroquine in incomplete lupus_____________
Manuscript number (if known):___________________ar-25-0931.R1____

In the interest of transparency, we ask you to disclose all relationships/activities/interests listed below that are related to the content of your manuscript. "Related" means any relation with for-profit or not-for-profit third parties whose interests may be affected by the content of the manuscript. Disclosure represents a commitment to transparency and does not necessarily indicate a bias. If you are in doubt about whether to list a relationship/activity/interest, it is preferable that you do so.

The following questions apply to the author's relationships/activities/interests as they relate to the current manuscript only.

The author's relationships/activities/interests should be defined broadly. For example, if your manuscript pertains to the epidemiology of hypertension, you should declare all relationships with manufacturers of antihypertensive medication, even if that medication is not mentioned in the manuscript.

In item #1 below, report all support for the work reported in this manuscript without time limit. For all other items, the time frame for disclosure is the past 36 months.

		**Name all entities with whom you have this relationship or indicate none (add rows as needed)**	**Specifications/Comments (e.g., if payments were made to you or to your institution)**
		**Time frame: Since the initial planning of the work**	
1	All support for the present manuscript (e.g., funding, provision of study materials, medical writing, article processing charges, etc.) **No time limit for this item.**	____	
		U01AR071077	institution
		**Time frame: past 36 months**	
2	Grants or contracts from any entity (if not indicated in item #1 above).	___X_ None	
3	Royalties or licenses	__X__ None	

4	Consulting fees	_X___ None	
5	Payment or honoraria for lectures, presentations, speakers bureaus, manuscript writing or educational events	__X__ None	
6	Payment for expert testimony	__X__ None	
7	Support for attending meetings and/or travel	__X__ None	
8	Patents planned, issued or pending	__X__ None	
9	Participation on a Data Safety Monitoring Board or Advisory Board	__X__ None	
10	Leadership or fiduciary role in other board, society, committee or advocacy group, paid or unpaid	__X__ None	
11	Stock or stock options	__X__ None	
12	Receipt of equipment, materials, drugs, medical writing, gifts or other services	__X__ None	
13	Other financial or non-financial interests	__X__ None	

Please place an "X" next to the following statement to indicate your agreement:

__X_ I certify that I have answered every question and have not altered the wording of any of the questions on this form.

ICMJE DISCLOSURE FORM

Date:________________08/30/2025____
Your Name:________Christopher Striebich____
Manuscript Title:___A Randomized, placebo-controlled trial of hydroxychloroquine in incomplete lupus_____________
Manuscript number (if known):___________________ar-25-0931.R1____

In the interest of transparency, we ask you to disclose all relationships/activities/interests listed below that are related to the content of your manuscript. "Related" means any relation with for-profit or not-for-profit third parties whose interests may be affected by the content of the manuscript. Disclosure represents a commitment to transparency and does not necessarily indicate a bias. If you are in doubt about whether to list a relationship/activity/interest, it is preferable that you do so.

The following questions apply to the author's relationships/activities/interests as they relate to the current manuscript only.

The author's relationships/activities/interests should be defined broadly. For example, if your manuscript pertains to the epidemiology of hypertension, you should declare all relationships with manufacturers of antihypertensive medication, even if that medication is not mentioned in the manuscript.

In item #1 below, report all support for the work reported in this manuscript without time limit. For all other items, the time frame for disclosure is the past 36 months.

		Name all entities with whom you have this relationship or indicate none (add rows as needed)	Specifications/Comments (e.g., if payments were made to you or to your institution)
		**Time frame: Since the initial planning of the work**	
1	All support for the present manuscript (e.g., funding, provision of study materials, medical writing, article processing charges, etc.) **No time limit for this item.**	____	
		U01AR071077	U Colorado/subcontract
		**Time frame: past 36 months**	
2	Grants or contracts from any entity (if not indicated in item #1 above).	__X__ None	
3	Royalties or licenses	__X__ None	

4	Consulting fees	_X___ None	
5	Payment or honoraria for lectures, presentations, speakers bureaus, manuscript writing or educational events	__X__ None	
6	Payment for expert testimony	__X__ None	
7	Support for attending meetings and/or travel	__X__ None	
8	Patents planned, issued or pending	__X__ None	
9	Participation on a Data Safety Monitoring Board or Advisory Board	__X__ None	
10	Leadership or fiduciary role in other board, society, committee or advocacy group, paid or unpaid	__X__ None	
11	Stock or stock options	__X__ None	
12	Receipt of equipment, materials, drugs, medical writing, gifts or other services	__X__ None	
13	Other financial or non-financial interests	__X__ None	

Please place an "X" next to the following statement to indicate your agreement:

__X_ I certify that I have answered every question and have not altered the wording of any of the questions on this form.

ICMJE DISCLOSURE FORM

Date: 8/28/2025

Your Name: Sonali Narain

Manuscript Title: A randomized, placebo-controlled trial of hydroxychloroquine in incomplete lupus

Manuscript Number (if known): Ar-25-0931

In the interest of transparency, we ask you to disclose all relationships/activities/interests listed below that are related to the content of your manuscript. "Related" means any relation with for-profit or not-for-profit third parties whose interests may be affected by the content of the manuscript. Disclosure represents a commitment to transparency and does not necessarily indicate a bias. If you are in doubt about whether to list a relationship/activity/interest, it is preferable that you do so.

The author's relationships/activities/interests should be defined broadly. For example, if your manuscript pertains to the epidemiology of hypertension, you should declare all relationships with manufacturers of antihypertensive medication, even if that medication is not mentioned in the manuscript.

In item #1 below, report all support for the work reported in this manuscript without time limit. For all other items, the time frame for disclosure is the past 36 months.

		Name all entities with whom you have this relationship or indicate none (add rows as needed)	Specifications/Comments (e.g., if payments were made to you or to your institution)
		**Time frame: Since the initial planning of the work**	
1	All support for the present manuscript (e.g., funding, provision of study materials, medical writing, article processing charges, etc.) **No time limit for this item.**	☐	
		NIH U01AR071077	All payments to institutions.
			Click the tab key to add additional rows.
		**Time frame: past 36 months**	
2	Grants or contracts from any entity (if not indicated in item #1 above).	☐ **None**	
		Janssen	All payments to Institution
		BMS	
		Novrtis	
		Sanofi	
		Kyverna	
		Boehringer Ingelheim (Narain)	

		Name all entities with whom you have this relationship or indicate none (add rows as needed)	Specifications/Comments (e.g., if payments were made to you or to your institution)
**3**	Royalties or licenses	☒ **None**	
**4**	Consulting fees	☒ **None**	
**5**	Payment or honoraria for lectures, presentations, speakers bureaus, manuscript writing or educational events	☐ **None**	
		RheumNow	Narain
**6**	Payment for expert testimony	☒ **None**	
**7**	Support for attending meetings and/or travel	☒ **None**	
**8**	Patents planned, issued or pending	☒ **None**	
**9**	Participation on a Data Safety Monitoring Board or Advisory Board	☒ **None**	
**10**	Leadership or fiduciary role in other board, society,	☒ **None**	

		Name all entities with whom you have this relationship or indicate none (add rows as needed)	Specifications/Comments (e.g., if payments were made to you or to your institution)
	committee or advocacy group, paid or unpaid		
**11**	Stock or stock options	☒ **None**	
**12**	Receipt of equipment, materials, drugs, medical writing, gifts or other services	☒ **None**	
**13**	Other financial or non-financial interests	☒ **None**	

Please place an "X" next to the following statement to indicate your agreement:

☒ I certify that I have answered every question and have not altered the wording of any of the questions on this form.

ICMJE DISCLOSURE FORM

Date: 8/28/2025

Your Name: Benjamin F Chong

Manuscript Title: A randomized, placebo-controlled trial of hydroxychloroquine in incomplete lupus

Manuscript Number (if known): Ar-25-0931

In the interest of transparency, we ask you to disclose all relationships/activities/interests listed below that are related to the content of your manuscript. "Related" means any relation with for-profit or not-for-profit third parties whose interests may be affected by the content of the manuscript. Disclosure represents a commitment to transparency and does not necessarily indicate a bias. If you are in doubt about whether to list a relationship/activity/interest, it is preferable that you do so.

The author's relationships/activities/interests should be defined broadly. For example, if your manuscript pertains to the epidemiology of hypertension, you should declare all relationships with manufacturers of antihypertensive medication, even if that medication is not mentioned in the manuscript.

In item #1 below, report all support for the work reported in this manuscript without time limit. For all other items, the time frame for disclosure is the past 36 months.

		Name all entities with whom you have this relationship or indicate none (add rows as needed)	Specifications/Comments (e.g., if payments were made to you or to your institution)
**Time frame: Since the initial planning of the work**			
**1**	All support for the present manuscript (e.g., funding, provision of study materials, medical writing, article processing charges, etc.) **No time limit for this item.**	☐	
		NIH U01AR071077	All payments to institutions.
		NIH U34AR06739201	
		K23AR061441	Click the tab key to add additional rows.
**Time frame: past 36 months**			
**2**	Grants or contracts from any entity (if not indicated in item #1 above).	☐ **None**	
		Daavlin Corp	Institution - UTSW
		EMD Serono	Institution - UTSW

		Name all entities with whom you have this relationship or indicate none (add rows as needed)	Specifications/Comments (e.g., if payments were made to you or to your institution)
**3**	Royalties or licenses	☐ **None**	
		MAPI research trust	UTSW and to Chong
**4**	Consulting fees	☐ **None**	
		BMS	Chong
		Biogen	Chong
		Horizon Therapeutics	Chong
		EMD Serono	Chong
		Amgen	Chong
		AstraZeneca	Chong
		Lupus Research Alliance	Chong
**5**	Payment or honoraria for lectures, presentations, speakers bureaus, manuscript writing or educational events	☐ **None**	
		Amgen	Chong
		Cesas Medical	Chong
**6**	Payment for expert testimony	☒ **None**	
**7**	Support for attending meetings and/or travel	☐ **None**	
		Amgen	Chong
**8**	Patents planned, issued or pending	☒ **None**	
**9**	Participation on a Data Safety Monitoring Board or Advisory Board	☐ **None**	
		Biogen	Chong
		BMS	Chong

		Name all entities with whom you have this relationship or indicate none (add rows as needed)	Specifications/Comments (e.g., if payments were made to you or to your institution)
**10**	Leadership or fiduciary role in other board, society, committee or advocacy group, paid or unpaid	☐ **None**	
		Rheum/Derm Society Director at-large	Chong / unpaid position
**11**	Stock or stock options	☒ **None**	
**12**	Receipt of equipment, materials, drugs, medical writing, gifts or other services	☒ **None**	
**13**	Other financial or non-financial interests	☒ **None**	

Please place an "X" next to the following statement to indicate your agreement:

☒ I certify that I have answered every question and have not altered the wording of any of the questions on this form.

ICMJE DISCLOSURE FORM

Date:________________08/30/2025____
Your Name:________Fan He
Manuscript Title:___A Randomized, placebo-controlled trial of hydroxychloroquine in incomplete lupus_____________
Manuscript number (if known):___________________ar-25-0931.R1____

In the interest of transparency, we ask you to disclose all relationships/activities/interests listed below that are related to the content of your manuscript. "Related" means any relation with for-profit or not-for-profit third parties whose interests may be affected by the content of the manuscript. Disclosure represents a commitment to transparency and does not necessarily indicate a bias. If you are in doubt about whether to list a relationship/activity/interest, it is preferable that you do so.

The following questions apply to the author's relationships/activities/interests as they relate to the current manuscript only.

The author's relationships/activities/interests should be defined broadly. For example, if your manuscript pertains to the epidemiology of hypertension, you should declare all relationships with manufacturers of antihypertensive medication, even if that medication is not mentioned in the manuscript.

In item #1 below, report all support for the work reported in this manuscript without time limit. For all other items, the time frame for disclosure is the past 36 months.

		**Name all entities with whom you have this relationship or indicate none (add rows as needed)**	**Specifications/Comments (e.g., if payments were made to you or to your institution)**
**Time frame: Since the initial planning of the work**			
1	All support for the present manuscript (e.g., funding, provision of study materials, medical writing, article processing charges, etc.) **No time limit for this item.**	____	
		U01AR071077	institution
**Time frame: past 36 months**			
2	Grants or contracts from any entity (if not indicated in item #1 above).	___X_ None	
3	Royalties or licenses	__X__ None	

4	Consulting fees	_X___ None	
5	Payment or honoraria for lectures, presentations, speakers bureaus, manuscript writing or educational events	__X__ None	
6	Payment for expert testimony	__X__ None	
7	Support for attending meetings and/or travel	__X__ None	
8	Patents planned, issued or pending	__X__ None	
9	Participation on a Data Safety Monitoring Board or Advisory Board	__X__ None	
10	Leadership or fiduciary role in other board, society, committee or advocacy group, paid or unpaid	__X__ None	
11	Stock or stock options	__X__ None	
12	Receipt of equipment, materials, drugs, medical writing, gifts or other services	__X__ None	
13	Other financial or non-financial interests	__X__ None	

Please place an "X" next to the following statement to indicate your agreement:

__X_ I certify that I have answered every question and have not altered the wording of any of the questions on this form.

ICMJE DISCLOSURE FORM

Date:________________08/30/2025____
Your Name:________Eric W. Schaefer____
Manuscript Title:___A Randomized, placebo-controlled trial of hydroxychloroquine in incomplete lupus_____________
Manuscript number (if known):___________________ar-25-0931.R1____

In the interest of transparency, we ask you to disclose all relationships/activities/interests listed below that are related to the content of your manuscript. "Related" means any relation with for-profit or not-for-profit third parties whose interests may be affected by the content of the manuscript. Disclosure represents a commitment to transparency and does not necessarily indicate a bias. If you are in doubt about whether to list a relationship/activity/interest, it is preferable that you do so.

The following questions apply to the author's relationships/activities/interests as they relate to the current manuscript only.

The author's relationships/activities/interests should be defined broadly. For example, if your manuscript pertains to the epidemiology of hypertension, you should declare all relationships with manufacturers of antihypertensive medication, even if that medication is not mentioned in the manuscript.

In item #1 below, report all support for the work reported in this manuscript without time limit. For all other items, the time frame for disclosure is the past 36 months.

		Name all entities with whom you have this relationship or indicate none (add rows as needed)	Specifications/Comments (e.g., if payments were made to you or to your institution)
**Time frame: Since the initial planning of the work**			
1	All support for the present manuscript (e.g., funding, provision of study materials, medical writing, article processing charges, etc.) **No time limit for this item.**	____	
		U01AR071077	institution
**Time frame: past 36 months**			
2	Grants or contracts from any entity (if not indicated in item #1 above).	_X___ None	
3	Royalties or licenses	__X__ None	

4	Consulting fees	_X___ None	
5	Payment or honoraria for lectures, presentations, speakers bureaus, manuscript writing or educational events	__X__ None	
6	Payment for expert testimony	__X__ None	
7	Support for attending meetings and/or travel	__X__ None	
8	Patents planned, issued or pending	__X__ None	
9	Participation on a Data Safety Monitoring Board or Advisory Board	__X__ None	
10	Leadership or fiduciary role in other board, society, committee or advocacy group, paid or unpaid	__X__ None	
11	Stock or stock options	__X__ None	
12	Receipt of equipment, materials, drugs, medical writing, gifts or other services	__X__ None	
13	Other financial or non-financial interests	__X__ None	

Please place an "X" next to the following statement to indicate your agreement:

__X_ I certify that I have answered every question and have not altered the wording of any of the questions on this form.

ICMJE DISCLOSURE FORM

Date:________________08/30/2025____
Your Name:________Vernon Chinchilli____
Manuscript Title:___A Randomized, placebo-controlled trial of hydroxychloroquine in incomplete lupus_____________
Manuscript number (if known):___________________ar-25-0931.R1____

In the interest of transparency, we ask you to disclose all relationships/activities/interests listed below that are related to the content of your manuscript. "Related" means any relation with for-profit or not-for-profit third parties whose interests may be affected by the content of the manuscript. Disclosure represents a commitment to transparency and does not necessarily indicate a bias. If you are in doubt about whether to list a relationship/activity/interest, it is preferable that you do so.

The following questions apply to the author's relationships/activities/interests as they relate to the current manuscript only.

The author's relationships/activities/interests should be defined broadly. For example, if your manuscript pertains to the epidemiology of hypertension, you should declare all relationships with manufacturers of antihypertensive medication, even if that medication is not mentioned in the manuscript.

In item #1 below, report all support for the work reported in this manuscript without time limit. For all other items, the time frame for disclosure is the past 36 months.

		**Name all entities with whom you have this relationship or indicate none (add rows as needed)**	**Specifications/Comments (e.g., if payments were made to you or to your institution)**
		**Time frame: Since the initial planning of the work**	
1	All support for the present manuscript (e.g., funding, provision of study materials, medical writing, article processing charges, etc.) **No time limit for this item.**	____	
		U01AR071077	institution
		U34AR06739201	institution
		**Time frame: past 36 months**	
2	Grants or contracts from any entity (if not indicated in item #1 above).	__X__ None	
3	Royalties or licenses	__X__ None	

4	Consulting fees	_X___ None	
5	Payment or honoraria for lectures, presentations, speakers bureaus, manuscript writing or educational events	__X__ None	
6	Payment for expert testimony	_X___ None	
7	Support for attending meetings and/or travel	___X_ None	
8	Patents planned, issued or pending	_X___ None	
9	Participation on a Data Safety Monitoring Board or Advisory Board	__X__ None	
10	Leadership or fiduciary role in other board, society, committee or advocacy group, paid or unpaid	_X___ None	
11	Stock or stock options	__X__ None	
12	Receipt of equipment, materials, drugs, medical writing, gifts or other services	__X__ None	
13	Other financial or non-financial interests	__X__ None	

Please place an "X" next to the following statement to indicate your agreement:

__X_ I certify that I have answered every question and have not altered the wording of any of the questions on this form.

ICMJE DISCLOSURE FORM

**Date:**	8/28/2025
**Your Name:**	David R Karp
**Manuscript Title:**	A randomized, placebo-controlled trial of hydroxychloroquine in incomplete lupus
**Manuscript Number (if known):**	Ar-25-0931

In the interest of transparency, we ask you to disclose all relationships/activities/interests listed below that are related to the content of your manuscript. "Related" means any relation with for-profit or not-for-profit third parties whose interests may be affected by the content of the manuscript. Disclosure represents a commitment to transparency and does not necessarily indicate a bias. If you are in doubt about whether to list a relationship/activity/interest, it is preferable that you do so.

The author's relationships/activities/interests should be defined broadly. For example, if your manuscript pertains to the epidemiology of hypertension, you should declare all relationships with manufacturers of antihypertensive medication, even if that medication is not mentioned in the manuscript.

In item #1 below, report all support for the work reported in this manuscript without time limit. For all other items, the time frame for disclosure is the past 36 months.

		Name all entities with whom you have this relationship or indicate none (add rows as needed)	Specifications/Comments (e.g., if payments were made to you or to your institution)
		**Time frame: Since the initial planning of the work**	
**1**	All support for the present manuscript (e.g., funding, provision of study materials, medical writing, article processing charges, etc.) **No time limit for this item.**	☐	
		NIH U01AR071077	All payments to institution.
		NIH U34AR06739201	
			Click the tab key to add additional rows.
		**Time frame: past 36 months**	
**2**	Grants or contracts from any entity (if not indicated in item #1 above).	☐ **None**	
		BMS	Institution - UTSW
		Biogen	Institution - UTSW
		Celgene	Institution - UTSW
		Eli Lily	Institution - UTSW
		Novartis	Institution - UTSW
		UCB	Institution - UTSW

		Name all entities with whom you have this relationship or indicate none (add rows as needed)	Specifications/Comments (e.g., if payments were made to you or to your institution)
**3**	Royalties or licenses	☒ **None**	
**4**	Consulting fees	☐ **None**	
		Ampel Biosciences	Karp
**5**	Payment or honoraria for lectures, presentations, speakers bureaus, manuscript writing or educational events	☐ **None**	
		RheumNow	Karp
**6**	Payment for expert testimony	☒ **None**	
**7**	Support for attending meetings and/or travel	☒ **None**	
**8**	Patents planned, issued or pending	☒ **None**	
**9**	Participation on a Data Safety Monitoring Board or Advisory Board	☐ **None**	
		PPD/Sanofi	Karp
		NIH – DSMB, U01AR080985	Karp
		PPD/Zura	Karp
		Prometrika/Caribou	Karp

		Name all entities with whom you have this relationship or indicate none (add rows as needed)	Specifications/Comments (e.g., if payments were made to you or to your institution)
**10**	Leadership or fiduciary role in other board, society, committee or advocacy group, paid or unpaid	☒ **None**	
**11**	Stock or stock options	☒ **None**	
**12**	Receipt of equipment, materials, drugs, medical writing, gifts or other services	☒ **None**	
**13**	Other financial or non-financial interests	☒ **None**	

Please place an "X" next to the following statement to indicate your agreement:

☒ I certify that I have answered every question and have not altered the wording of any of the questions on this form.
