# Supplementary material for: A Randomized, Placebo‐Controlled Trial of Hydroxychloroquine in Incomplete Lupus
Source: Arthritis Rheumatol. 2025 Dec 12;78(4):870–9. doi: 10.1002/art.43391 (PMC13054461; doi:10.1002/art.43391)
Supplement: Supplementary file 2 — Data S1: Supporting Information [file ART-78-870-s003.pdf]

Clinical Research Protocol
STUDY OF ANTIMALARIALS IN INCOMPLETE LUPUS ERYTHEMATOSUS (SMILE)

Protocol Number:	ClinicalTrials.gov – NCT03030118
Version Date:	04/01/2022
Investigational Product:	Hydroxychloroquine sulfate
IND Number:	Exempt per FDA
Development Phase:	Phase 2
Sponsor:	Nancy J. Olsen, M.D., M.S.<br>Penn State MS Hershey Medical Center<br>500 University Drive<br>Hershey, PA 17033-0850
Funding Organization:	National Institute of Arthritis Musculoskeletal and Skin Disorders U01 AR071077
Principal Investigator:	Name: Nancy J. Olsen, M.D., M.S.<br>Telephone: 717-531-5384<br>Fax: 717-531-8274<br>E-mail: nolsen@pennstatehealth.psu.edu
Medical Monitor:	Name: Joan T. Merrill, M.D.<br>Telephone: 405-271-7805<br>Fax: 405-271-8797<br>E-mail: joan-merrill@omrf.org
Coordinating Center:	Name: Duanping Liao, M.D., PhD.<br>Telephone: 717-531-4149<br>Fax: 717-531-5779<br>E-mail: dliao@phs.psu.edu

Approval:

Nancy J. Olsen, Principal Investigator/Sponsor   04/01/2022

PI or Sponsor Signature (Name and Title)   *Date*

This confidential information about an investigational product is provided for the exclusive use of investigators of this product and is subject to recall at any time. The information in this document may not be disclosed unless federal or state law or regulations require such disclosure. Subject to the foregoing, this information may be disclosed only to those persons involved in the study who have a need to know, with the obligation not to further disseminate this information.

PROTOCOL AGREEMENT

I have read the protocol specified below. In my formal capacity as Investigator, my duties include ensuring the safety of the study participants enrolled under my supervision and providing Dr. Nancy J. Olsen with complete and timely information, as outlined in the protocol. It is understood that all information pertaining to the study will be held strictly confidential and that this confidentiality requirement applies to all study staff at this site. Furthermore, on behalf of the study staff and myself, I agree to maintain the procedures required to carry out the study in accordance with accepted GCP principles and to abide by the terms of this protocol.

Protocol Number: N/A

Protocol Title: Study of Anti-Malarials in Incomplete Lupus Erythematosus (SMILE)

Protocol Date: 04/01/2022

______________________________ ______________

Investigator Signature *Date*

____

Print Name and Title

Site # ______________

Site Name ______________________________

Address ______________________________

____

____

____

Phone Number ______________________________

TABLE OF CONTENTS

1 Background 13
1.1 Overview of Non-Clinical Studies 13
1.2 Overview of Clinical Studies 14
2 Study Rationale 16
2.1 Risk / Benefit Assessment 18
3 Study Objectives 19
3.1 Primary Objective 19
3.2 Secondary Objectives 19
4 Study Design 19
4.1 Study Overview 19
5 Criteria for Evaluation 20
5.1 Primary Efficacy Endpoint 20
5.2 Secondary Efficacy Endpoints 20
5.3 Safety Evaluations 20
5.4 Other Evaluations 20
6 Subject Selection 21
6.1 Study Population 21
6.2 Inclusion Criteria 21
6.3 Exclusion Criteria 21
6.4 Premature Termination of a Participant from the Study 22
7 Concurrent Medications 22
7.1 Allowed Medications and Treatments 22
7.2 Prohibited Medications and Treatments 23
7.3 Contraception 23
8 Study Procedures 24
8.1 Visit Windows 24
8.2 Enrollment, Randomization and Blinding 24
8.3 Study Treatments 25
8.4 Supply of Study Drug at the Site 25
8.5 Study Drug Accountability 26
8.6 Discontinuation of Study Drug 26
9 Study Assessments and Guidelines 27
9.1 Clinical Assessments 27
9.2 Clinical Laboratory Measurements 28
9.3 Hematology 29
9.4 Blood Chemistry 29
9.5 Planned Mechanistic Assays 29
9.6 Future/Unplanned Studies 30
9.7 Specimen Logistics 30
9.8 Specimen Tracking Procedures 30
9.9 Specimen Storage 31
10 Evaluations by Visit 31

10.2 Visit 2 and 12 (Week 1-5 and week 100-104) ... 31
10.3 Visit 3 (Up to 5 weeks after Visit 1) ... 32
10.4 Visits 4, 5, 6, and 8, 9, 10 (Weeks 16, 28, 40, and weeks 64, 76, and 88 ± 7 days) ... 32
10.5 Visits 7 and 11 (Weeks 52 and 100 ± 7 days) ... 33
10.6 Unscheduled Visit / Early Withdrawal Visit ... 33

11 Adverse Event Documentation and Reporting ... 34
11.1 Adverse Events ... 34
11.2 Serious Adverse Events (SAE) ... 36
11.3 Medical Monitoring ... 36

12 Discontinuation and Replacement of Subjects ... 37
12.1 Early Discontinuation of Study Drug ... 37
12.3 Withdrawal of Subjects from the Study ... 37
12.4 Replacement of Subjects ... 38

13 Protocol Violations ... 38

14 Data Safety Monitoring ... 38

15 Statistical Methods and Considerations ... 38
15.1 Data Sets Analyzed ... 38
15.2 Demographic and Baseline Characteristics ... 39
15.3 Analysis of Primary Endpoint ... 39
15.4 Analysis of Secondary Endpoints ... 41
15.5 Interim Analysis ... 43
15.6 Sample Size and Randomization ... 43

16 Data Collection, Retention, and Monitoring ... 44
16.1 Data Collection Instruments ... 44
16.2 Data Management Procedures ... 44
16.3 Data Quality Control and Reporting ... 44
16.4 Archival of Data ... 44
16.5 Availability and Retention of Investigational Records ... 45
16.6 Monitoring ... 45
16.7 Subject Confidentiality ... 45

17 Administrative, Ethical, and reporting Considerations ... 46
17.1 Multi-Site Research Administration ... 46
17.2 Protocol Amendments ... 47
17.3 Institutional Review Boards and Independent Ethics Committees ... 47
17.4 Informed Consent Form ... 48
17.5 Publications ... 48
17.6 Investigator Responsibilities ... 48

18 References ... 49

Appendix 1 – Schedule of Study Visits ... 52

Appendix 2 - Systemic lupus international collaborating clinics 2012 criteria for the classification of systemic lupus erythematosus[1] ... 54

Appendix 3 - 1997 update of the 1982 American college of rheumatology revised criteria for classification of systemic lupus erythematosus[1] ... 57

LIST OF ABBREVIATIONS

**ACR**	American College of Rheumatology
**AE**	adverse event
**ALT**	alanine aminotransferase
**AST**	aspartate aminotransferase
**BUN**	blood urea nitrogen
**CCP**	Cyclic Citrullinated Peptide
**CFR**	Code of Federal Regulations
**CLASI**	Cutaneous Lupus Disease Area and Severity Index
**CMSU**	University of Rochester Clinical Materials Services Unit
**CRF**	case report form
**CSQ**	Connective Tissue Disease Screening Questionnaire
**DFE**	dilated fundoscopic examination
**DSMB**	Data Safety Monitoring Board
**FDA**	Food and Drug Administration
**GCP**	Good Clinical Practice
**G6PD**	glucose-6-phosphate dehydrogenase
**HCQ**	hydroxychloroquine
**HIPAA**	Health Insurance Portability and Accountability Act of 1996
**HVF**	Humphrey visual fields
**ICF**	informed consent form
**ICH**	International Conference on Harmonization
**IEC**	Independent Ethics Committee
**ILE**	incomplete lupus erythematosus
**IRB**	Institutional Review Board
**IVIG**	intravenous immunoglobulin
**LR**	Likelihood ratio
**MAR**	missing-at-random
**ML**	maximum likelihood
**PI**	Principal Investigator
**PROMIS**	Patient Reported Outcomes Measurement Information System
**PSU**	Pennsylvania State University
**SAE**	serious adverse event
**SD-OCT**	spectral domain ocular coherence tomography
**SLEDAI-2K**	Systemic Lupus Erythematosus Disease Activity Index 2000
**SGOT**	serum glutamic oxaloacetic transaminase
**SGPT**	serum glutamate pyruvate transaminase
**SLE**	Systemic Lupus Erythematosus

**SLICC**	Systemic Lupus International Collaborating Clinics
**UIP**	University of Iowa Pharmaceuticals

PROTOCOL SYNOPSIS

**TITLE**	**S**tudy of Anti-**M**alarials in **I**ncomplete **L**upus **E**rythematosus (**SMILE**)
**SPONSOR**	Nancy J. Olsen, M.D., Penn State Hershey Medical Center
**FUNDING ORGANIZATION**	National Institute of Arthritis, Musculoskeletal and Skin Diseases
**NUMBER OF SITES**	Seven
**RATIONALE**	Onset of systemic lupus erythematosus is preceded by a preclinical phase characterized by expression of autoantibodies and nonspecific clinical symptoms. Hydroxychloroquine is a treatment for lupus that is widely used based on longstanding experience and a very good safety profile. Retrospective analyses suggest that treatment with hydroxychloroquine may postpone onset of disease, and clinical experience supports a role for this drug in improving clinical outcomes. However, prospective studies that prove and quantify the efficacy of hydroxychloroquine in the preclinical phase of lupus have not been done. This study will test the hypothesis that early hydroxychloroquine use can modify immune responses and prevent accumulation of abnormalities that define systemic lupus.
**STUDY DESIGN**	Double-blind, placebo-controlled, randomized trial of hydroxychloroquine vs. placebo in a 1:1 allocation.
**PRIMARY OBJECTIVE**	The primary objective is to assess the ability of HCQ to prevent the acquisition of SLE criteria in persons at risk for the disease.
**SECONDARY OBJECTIVES**	The secondary objectives are to determine the ability of HCQ to 1) lessen lupus disease activity scores, 2) improve patient reported outcomes, and 3) prevent the accumulation of autoantibodies, inflammatory cytokines, and other biomarkers.
**NUMBER OF SUBJECTS**	240 subjects (120 hydroxychloroquine/120 placebo)
**SUBJECT SELECTION CRITERIA**	<u>Inclusion Criteria</u>:<br>1. Between 15 and 49 years of age, inclusive, at Visit 1.<br>2. Anti-nuclear antibody (ANA) titer of 1:80, or greater, as determined by immunofluorescence assay (IFA).<br>3. Participants must have at least one (but not three or more) additional clinical or laboratory criterion from the 2012 Systemic Lupus International Collaborating Clinics (SLICC) classification criteria.

	4. Written informed consent (and assent when applicable) obtained from subject or subject's legal representative and ability for subject to comply with the requirements of the study.<br><br><u>Exclusion Criteria</u>:<br>**1.** The subject meets the 2012 SLICC classification criteria for SLE at Visit 1 (i.e., ANA plus 3 other criteria, or ANA plus biopsy-proven lupus nephritis).<br>2. The subject has been diagnosed with another autoimmune disorder, other than autoimmune thyroid conditions.<br>3. The subject has fibromyalgia, based on clinical history and exam.<br>4. The subject has previously been or is currently being treated with oral antimalarial agents including hydroxychloroquine, chloroquine, or quinacrine.<br>5. The subject is currently or has been treated with immunosuppressive, immune modifying, or cytotoxic medications as listed in Section 7.2.<br>6. Use of any investigational agent within the preceding 12 months.<br>7. History of primary immunodeficiency.<br>8. Active bacterial, viral, fungal, or opportunistic infection.<br>9. Evidence of infection with human immunodeficiency virus (HIV), Hepatitis B, or Hepatitis C.<br>10. Concomitant malignancy or history of malignancy with the exception of adequately treated basal or squamous cell carcinoma of the skin, or carcinoma in situ of the cervix.<br>11. The subject has significant findings on ophthalmological examination that, in the opinion of the examining Ophthalmologist, prevent safe use of hydroxychloroquine.<br>12. The subject has other contraindications to treatment with hydroxychloroquine including pre-existing ocular disease, hepatic impairment, psoriasis, porphyria, or allergy to the drug or class.<br>13. Co-morbidities requiring systemic corticosteroid therapy greater than 10 mg of prednisone per day, or equivalent, or a change in corticosteroid dose within the 4 weeks prior to Visit 1.<br>14. Pregnant, breastfeeding, or unwilling to practice birth control during participation in the study.<br>15. Presence of a condition or abnormality that in the opinion of the Investigator would compromise the safety of the patient or the quality of the data.<br>16. Inability to comply with the study visit schedule and procedures.

**TEST PRODUCT, DOSE, AND ROUTE OF ADMINISTRATION**	Hydroxychloroquine sulfate, 400 mg/day for subjects weighing more than 40 kg (200 mg/d for subjects weighing 40 kg or less)<br>Hydroxychloroquine will be administered daily for 96 weeks. Capsules will be taken with a meal once a day.
**CONTROL PRODUCT, DOSE AND ROUTE OF ADMINISTRATION**	Matching placebo for hydroxychloroquine<br>Placebo will be administered daily for 96 weeks. Capsules will be taken with a meal once a day.
**DURATION OF SUBJECT PARTICIPATION AND DURATION OF STUDY**	Subjects will be on study for up to 104 weeks<br>**Screening and Ophthalmology Exam:** up to 4 weeks<br>**Treatment:** 96 weeks<br>**Follow-up Ophthalmology Exam:** up to 4 weeks<br>The total duration of the study is expected to be 48 months. 24 months for subject recruitment and 24 months for final subject follow-up.
**CONCOMITANT MEDICATIONS**	Allowed:<br>1. Use of standard (i.e., FDA approved) doses of over-the-counter or prescription non-steroidal anti-inflammatory agents (NSAIDs).<br>2. Low-dose (81 mg) aspirin for cardioprotection is allowed at any time during the trial. Its use must be documented.<br>3. Use of oral corticosteroids equivalent to 10 mg or less of prednisone for lupus-related conditions is permitted at entry into the study. Oral corticosteroids equivalent to 10 mg or less of prednisone may be started after week 4 (baseline) for lupus-related conditions. The corticosteroid dose can be increased or decreased at the discretion of the site physician as long as the dose does not exceed 10 mg of prednisone equivalent per day. Participants may use higher dose corticosteroids (i.e., greater than 10 mg per day of prednisone equivalent per day) to treat conditions that are not SLICC criteria for the classification of lupus such as asthma, contact dermatitis, etc., at the discretion of the site physician.<br>4. Topical and/or intralesional corticosteroids to control skin disease are permitted, if subjects do not respond to study drug. Their use must be recorded at each visit.<br>5. Topical calcineurin inhibitors including pimecrolimus and tacrolimus to control skin disease if subjects do not respond to study drug. Their use must be recorded at each visit.<br>6. Subjects may receive one intra-articular corticosteroid injection to control arthritis in a single joint that does not

	respond to study drug during the entire 24 months of study treatment.<br>7. Vitamin D preparations may be used by subjects. Their use must be recorded at each visit.<br>Prohibited:<br>1. Oral corticosteroids in excess of 10 mg per day of prednisone, or equivalent, if used to treat lupus-related symptoms.<br>2. Intramuscular or intravenous corticosteroids, if used to treat lupus-related symptoms<br>3. More than <u>one</u> intra-articular corticosteroid injection during the entire 24 months of study treatment.<br>4. Methotrexate (oral or parenteral), leflunomide, sulfasalazine, azathioprine, tofacitinib, mycophenolate, thalidomide, dapsone, danazol, minocycline, oral cyclophosphamide, and penicillamine may not be used at any time before or during the study.<br>5. TNF inhibitors: infliximab, adalimumab, golimumab, certolizomab, and etanercept may not be used at any time before or during the study.<br>6. Abatacept, tocilizumab, rituximab, or belimumab may not be used at any time before or during the study.<br>7. Intravenous immunoglobulin (IVIG), plasmapheresis, or leukopheresis may not be used during the study.<br>8. Intravenous cyclophosphamide may not be used during the study.
**EFFICACY EVALUATIONS**	
PRIMARY ENDPOINT	• The primary endpoint is the increase in the number of clinical and/or laboratory features of SLE defined by the 2012 SLICC classification criteria between week 4 and week 100.
SECONDARY ENDPOINTS	• The proportion of participants who transition to a classification of SLE as defined by the 2012 SLICC classification criteria.<br>• The proportion of participants who transition to a classification of SLE by the 1997 revised ACR classification criteria.<br>• The change in Modified SLEDAI-2K score at weeks 16, 28, 40, 52, 64, 76, 88 and 100.<br>• The change in CLASI cutaneous lupus scores at weeks 16, 28, 40, 52, 64, 76, 88 and 100.

	• The frequency of participants with clinically relevant autoantibodies (anti-Sm, anti-RNP, anti-Ro, and anti-La) at weeks 28, 52, 76 and 100.<br>• The frequency of participants with abnormal levels of anti-DNA, C3, and C4 at weeks 28, 52, 76 and 100.<br>• The increase in disease damage score determined by the SLICC/ACR Damage Index scale between week 4 and week 100.<br>• The proportions of participants who start, stop, or modify their use of oral or topical corticosteroids during the study.<br>• Analysis of responses by race and ethnicity.
**OTHER EVALUATIONS**	• The change in the level of IgG, IgM, and IgA autoantibodies as measured on an autoantibody array at weeks 52 and 100.<br>• The change in the serum concentration of cytokines on a multiplex panel of 30 specificities at weeks 52 and 100.
**SAFETY EVALUATIONS**	• Development of changes seen on ophthalmological assessment including a dilated fundoscopic examination, spectral domain ocular coherence tomography, and Humphrey visual field testing.<br>• Safety as measured by physical examination, vital signs, adverse events, and laboratory tests.
**PLANNED INTERIM ANALYSES**	Approximately 12 months following the enrollment of the first patient an interim analysis for safety will be conducted by an independent data safety monitoring board appointed by NIAMS. Serious adverse events will be monitored by the committee on an ongoing basis throughout the study.
**STATISTICS**<br>**Primary Analysis Plan**	The primary outcome variable in this double blind, randomized trial is the number of SLICC classification criteria, assessed, every 12 weeks over a 96-week period. Ordinal logistic regression analysis with random effects will be applied to compare the estimated slopes for the HCQ and placebo groups with respect to the SLICC classification criteria count over the 96-week follow-up period. The primary null hypothesis is that the HCQ slope equals the placebo slope, and its corresponding alternative hypothesis is that the HCQ slope does not equal the placebo slope. Thus, a two-sided test with a 0.05 significance level will be applied. The statistical model will account for the ordinal and non-decreasing properties of the SLICC classification criteria during the 96-week follow-up period, as well as account for the repeated measurements on each patient.

**Rationale for Number of Subjects**	The target sample size to be analyzed for this trial is 240 patients (120 randomized to each of the placebo and HCQ groups). For the primary outcome variable of the SLICC classification criteria count, it is anticipated that the probability of the SLICC classification criteria count increasing by at least one criterion during the minimum 96-week follow-up period is 0.4 for a placebo patient and 0.2 for and HCQ patient. There is 90% statistical power to reject the null hypothesis with a two-sided, 0.05 significance level if 192 subjects can be analyzed, allowing for 20% drop out over 96 weeks. Up to 300 subjects may need to be consented to account for screen failure prior to randomization.

1 BACKGROUND

Hydroxychloroquine sulfate, also available under the trade name Plaquenil®, was approved in 1955 by the United States FDA for the treatment of systemic lupus erythematosus. It is also approved for the treatment of discoid lupus erythematosus, rheumatoid arthritis, and malaria due to certain *Plasmodium* species[1]. Hydroxychloroquine sulfate is a 4-aminoquinolone in the anti-inflammatory and anti-malarial classes of pharmacologic agents. This drug also has immunomodulatory properties. Other drugs in the antimalarial class that have been used for treatment of lupus include chloroquine phosphate (Aralen®) and quinacrine (Atabrine®). The retinal toxicity of chloroquine makes it less desirable than hydroxychloroquine sulphate. Quinacrine is currently not available as a formulated preparation in the United States. For these reasons, hydroxychloroquine sulphate is the medication being used in this trial.

1.1 Overview of Non-Clinical Studies

Given the length of time that HCQ has been marketed in the United States, there is little relevant pre-clinical data. Studies were done in early stages of HCQ development that compared this agent to chloroquine[2]. In general, the lower toxicity of HCQ was apparent in studies using both agents administered intramuscularly to dogs. Doses of 25 mg/kg of hydroxychloroquine were well tolerated and gave mean peak plasma levels of 1400 μg/L. By contrast, no more than 6 mg/kg of chloroquine could be given, with mean peak plasma levels of only 140 μg/L. Dogs readily tolerated oral doses of 20 mg/kg HCQ given six days a week for 13 weeks while identical doses of chloroquine, given in another study daily (7 days per week) resulted in the death of three out of 4 animals within 19 days. Overall, the margin of safety comparing chloroquine to hydroxychloroquine was significant and it was estimated that HCQ was only 40% as toxic as chloroquine.

The pharmacokinetics and disposition of hydroxychloroquine sulphate have been characterized in the rat[2]. Tissue levels of HCQ in albino rats dosed orally with 40 mg/kg/day, 6 days per week for 3 months were analyzed. Results showed rapid accumulation of drug in multiple tissues during the first two weeks. Additional accumulation at a slower rate was seen into the third month. The order of increasing concentrations (lowest to highest) was: muscle, eye, heart, kidney, liver, lung and spleen. Other studies have shown that concentrations in skin and fat were just below muscle. After discontinuation of HCQ, all levels fell rapidly, by 80% in the first 8 days.

HCQ and chloroquine increase lysosomal pH, which may be responsible for altered antigen processing[3-5]. Other data suggest that blockade of endosomal Toll-like receptors (TLR) by anti-malarials may be independent of pH changes and rather involve direct binding of the drug to nucleic acid structures that target TLRs[6]. Production of Type I interferon is one consequence of TLR activation, so blockade of these receptors would be predicted to result in lower levels of this pivotal lupus cytokine [7] and this has been shown in SLE patients treated with HCQ[8]. HCQ inhibits calcium-channel-dependent T cell activation[9]; chloroquine mediates increases in interleukin-10, an immunosuppressive cytokine[10] and decreases in production of the pro-inflammatory cytokines TNF-alpha, IL6 and IL8[6].

1.2 Overview of Clinical Studies

Given the length of time that has passed since FDA approval, data from the currently used standard stages of development (Phase I-IV) are not available. However, HCQ is now the most commonly used drug for the treatment of SLE, endorsing its efficacy in this condition. The Canadian Hydroxychloroquine Study Group carried out a key study documenting this efficacy[11]. The design was a blinded, randomized discontinuation of HCQ. During the 6-month period following randomization to placebo or continued HCQ, the risk of flares was increased 2.5-fold in the placebo group. Other studies have shown efficacy in active SLE patients and use of HCQ is associated with renal remission when given in combination with other drugs such as mycophenolate mofetil[12]. Relevant to the current study in ILE are previous observations demonstrating that treatment of patients with incomplete lupus syndromes with HCQ results in a delay in the onset of full SLE as defined by the classification criteria[13,14]. A systematic review of the quality of evidence supporting clinical efficacy of HCQ in SLE including randomized trials and prospective and retrospective cohort studies showed that the antimalarial drugs significantly reduced disease activity and the rate of lupus flares[15]. Three studies also demonstrated a steroid-sparing effect.

Pharmacokinetics and pharmacodynamics:

Absorption: Hydroxychloroquine bioavailability in healthy volunteers is approximately 0.7. Bioavailability is highly variable using plasma data, and for this reason whole blood rather than plasma is preferred for measuring concentrations. The mean absorption half-life is 4 hours. Mean absorption in patients with rheumatoid arthritis is similar to that of healthy volunteers. Bioavailability of the enantiomers was not statistically different. Very severe malnutrition such as in patients with kwashiorkor can inhibit absorption. Recommended dosing is on a full stomach with a full glass of water or milk.

Distribution: Hydroxychloroquine binds avidly to tissues, especially melanin-containing tissues. Therefore, the volume of distribution in humans is very large[16]. Binding to serum proteins is about 50% measured in vitro. Uptake is greatest in mononuclear cells and is not detectable in polymorphonuclear cells.

Single-dose studies: The terminal half-life of hydroxychloroquine in blood has been estimated from single-dose studies as 50 +/- 16 days and the clearance form whole blood has been estimated as 96 ml/min.

Excretion: Renal clearance of unchanged hydroxychloroquine has been shown to account for only 21% of the administered dose.

Pharmacokinetic-pharmacodynamic considerations: Several studies have shown correlation between clinical efficacy and blood concentrations. Patients with concentrations of greater than 700 ng/ml may have a better overall clinical response to administration of hydroxychloroquine.

Efficacy:

The Canadian study cited above remains one of the cornerstones endorsing HCQ activity in reducing lupus flares. This was a blinded and randomized trial, so the evidence is level IB (evidence from at least one randomized controlled trial). Furthermore, a systematic review of antimalarials in SLE (not limited to HCQ) using the GRADE methodology was carried out by Ruiz-Irastorze et al. This group found 11 studies including, 4 that were randomized controlled designs, 4 that were prospective and 2 retrospective. The analysis showed that treatment with

antimalarials reduced SLE activity significantly and in most the effect was more than 50% reduction in activity levels.

Safety and Toxicity:

Hydroxychloroquine sulphate is generally well tolerated. Two types of symptomatic side effects that are seen relatively frequently are GI intolerance and cutaneous manifestations. These are usually not severe and may respond to reduction of the dose or may subside with continuing the dose. Rare serious toxicities can involve tissue damage to eyes (retinas)[17,18] or muscles (skeletal, cardiac) or cause hematological abnormalities. Overall, recent comprehensive reviews suggest that toxicity is low and most side effects are in the mild category. Specific types of toxicities are considered in the following paragraphs.

Gastrointestinal toxicities are most commonly nausea, vomiting and diarrhea, estimated to occur in up to 10% of HCQ patients. Other GI problems including decreased appetite, heartburn, abdominal distension and elevation of liver transaminases are rare. GI manifestations may resolve with time even if treatment is continued.

Cutaneous toxicities include changes in pigmentation. Most reported cases are related to other anti-malarials such as chloroquine and this is rarely seen with HCQ. A skin biopsy may show melanin granules and hemosiderin deposits. Pruritus may also occur.

Ophthalmologic toxicity is a more serious concern because it may be asymptomatic and can cause irreversible retinal damage. Eye toxicity is due to binding of the drug to the melanin in pigmented epithelial cells where damage to rods and cones may occur. Some of the early changes, in the category of pre-maculopathy, are thought to be reversible upon discontinuation of the drug. More advanced maculopathy shows depigmentation in the macula, with a pattern known as the bull's eye lesion. These changes may be irreversible even when drug treatment is discontinued. Progression of visual loss may also occur after the drug is stopped. The incidence of retinopathy with HCQ is low, and is definitely lower than with chloroquine. One recent retrospective study of 1207 patients treated with HCQ found definite eye toxicity in 0.08% and possible or probable toxicity in 0.4%. Toxicity in the eye is associated with higher doses and longer period of use, but the exact relationship is not understood.

Screening recommendations for ophthalmologic damage: While the overall incidence of retinal toxicity as the result of hydroxychloroquine is low, early detection of toxicity can prevent serious loss of vision. The 2011 recommendations from the American Academy of Ophthalmology recommend daily doses of up to 400 mg daily or 6.5 mg/kg ideal body weight for short individuals. Since the drug does not accumulate in adipose tissue, a recent analysis recommended that dose of 5 mg/kg of real body weight be used as the upper limit to avoid possible overdose[18]. In both studies, the risk of retinal toxicity rose after a cumulative dose of 1 kilogram of drug – approximately 82 months. A baseline screening eye examination is recommended within the first year of use and annual screening is recommended after 5 years of use. For higher risk patients, such as patients with baseline maculopathy, elderly patients or patients with liver or kidney dysfunction, screening is recommended annually. The screening procedures include an ocular examination with dilated retinal examination for detection of baseline retinal disorders, automated visual field testing with white 10-2 threshold testing and one of the following objective tests: 1) spectral domain optical coherence tomography (SD-OCT), 2) multifocal electroretinogram (mfERG), or 3) fundus autofluorscence (FAF)[17].

Cardiac toxicity occurs but is rare; less than 100 cases have been reported in the scientific literature. There are no recommendations for routine cardiac screening.

Neuromyotoxicity is also rare with fewer cases reported than cardiac toxicity. Manifestations that might suggest this complication include progressive weakness with insidious onset. Involvement is thought to usually start in proximal muscles of the legs; a peripheral neuropathy may be also present. Patients who develop proximal myopathy, neuropathy or cardiomyopathy who are being treated with HCQ should be considered as possibly having drug-induced toxicity. A muscle biopsy may be diagnostic.

For more detail refer to the FDA-approved Prescribing Information (http://www.accessdata.fda.gov/drugsatfda_docs/label/2007/009768s041lbl.pdf)

2 STUDY RATIONALE

Systemic lupus erythematosus (SLE) is a multi-organ disease with protean manifestations that afflicts primarily persons younger than 40 years of age[19]. Consequences of SLE include life-threatening organ damage, including lupus nephritis which is a major cause of end stage renal disease [20]. Other significant manifestations include seizures and cerebrovascular accidents, bone marrow insufficiency with anemia, thrombocytopenia and lymphopenia, polyserositis, blood clotting disorders and avascular necrosis. In the past decade, cardiovascular abnormalities have been recognized to be an intimate part of lupus, and SLE is an independent risk factor for the development of cardiovascular disease[21]. Treatments and approaches to therapy have improved in the past two decades, and mortality rates have markedly decreased. However, even the most intensive immunomodifying therapies available cannot reverse organ damage that is present at the time of clinical diagnosis in up to half of patients. Since peak ages of onset of SLE are in young persons, especially in the second to third decade of life, the consequence is a longstanding chronic illness with socioeconomic impact on the individual and society in general. A frontier in SLE research, therefore, is identification of individuals in early stages, when treatments have great potential to ameliorate or cure disease.

Study of the natural history of lupus prior to complete disease classification has been facilitated by the fact that clinical symptoms appear over time. This is made it possible to identify persons at risk for development of lupus who may have one or two features of the disease. These patients have been termed Incomplete Lupus Erythematosus, or ILE. One cohort of patients with ILE has been followed at UT Southwestern Medical Center[22,23]. Typically, these patients have an ANA and one or two other features of SLE. 22 patients were seen who had ILE were seen at baseline and at follow up visits an average of 2.4 years apart (range 0.5-6.5 yr.). In this time period, three of 22 patients progressed to SLE. One of the tools used to follow patients was an autoantigen microarray consisting of over 100 antigens previously described as targets of antibodies in lupus and other autoimmune conditions. The patients progressed from ILE to SLE had higher baseline levels of seven IgG autoantibodies. These antibodies were not necessarily ones commonly associated with SLE or available clinically. They included thyroid peroxidase, thyroglobulin, PCNA, hemocyanin, beta-2 microglobulin, threonine tRNA synthetase and liver cytosol. Levels of some of these autoantibodies were also significantly elevated in the final blood samples of subjects who progressed to SLE. Moreover, the total IgG autoreactivity increased significantly in the subjects who progressed to lupus.

In addition to autoantibodies, several other biomarkers were tested. In established SLE, there is up-regulation of genes driven by Type I interferons (e.g., interferon alpha or beta). The source of this cytokine is presumed to be plasmacytoid dendritic cells chronically simulated by immune complexes and resulting in perpetuation of innate and adaptive autoimmunity. As a group, subjects with ILE also had levels of mRNA for interferon alpha responsive genes that were intermediate between healthy controls and patients with SLE, suggesting that the increased level of autoantibodies was functionally relevant[24]. The ILE subjects also had intermediate levels of certain cytokines including MCP-1, EGF, VEGF, and eotaxin. By combining age, female gender, anti-nuclear antibody titer and overall autoantibody burden, the investigators were able to develop a scoring system that was highly predictive of the progression from ILE to SLE.

Blood relatives of lupus patients also represent an at-risk population for the development of SLE. This is most obvious in twin studies where the concordance rate for monozygotic twins developing lupus is between 24 and 69%, while the concordance for dizygotic twins is between 2 and 5%. Researchers at the Oklahoma Medical Research Foundation have followed 409 unaffected blood relatives of lupus patients (M. Monroe, unpublished). They were evaluated clinically at baseline and at an average of 6.4 years later. Blood was obtained for autoantibody and biomarker analysis. 45 blood relatives of lupus patients transitioned to SLE during this time. Subjects who transitioned had more autoantibodies present at baseline, particularly DNA and RNA binding antibodies. In addition, they had higher levels of some biomarkers, Including stem cell factor, MCP-1, MCP-3, and B lymphocyte stimulator (BLyS). They also have lower levels of anti-inflammatory cytokines including IL-10 and TGF-beta. Investigators also administered the SLE-specific portions of a validated, patient self-reported questionnaire, termed the Connective Tissue Disease Screening Questionnaire" (CSQ). This questionnaire is designed to elicit symptoms of lupus and other connective tissue diseases from patients. It has shown to have high validity and sufficient sensitivity and specificity to serve as an initial screening tool in high-risk populations. Using generalized estimating equations, a model that includes the number of SLE classification criteria at baseline, the baseline CSQ, and baseline concentrations of stem cell factor and TGF-beta, but not age, gender, race or autoantibody status was able to correctly classify 89% of blood relatives of SLE patients who would later go on to develop lupus.

These studies and many others support the current paradigm for the development of SLE and other autoimmune rheumatic diseases[25,26]. The development of autoantibodies, in and of themselves, may not be pathogenic. Their presence is influenced by the genetic makeup of the person, as well as environmental factors such as infections, exposure to UV light, tobacco smoke, nutritional factors, hormones, and the person's microbiome. These people are felt to have "benign autoimmunity". That is, they have evidence of serological autoimmunity without evidence of tissue damage. In a subset of these persons, the number and titer of autoantibodies increases, triggering activation of the inmate and adaptive immune systems. The triggers for such epitope spreading and expansion of autoantibody repertoires are not known. Ultimately, the level of inflammatory mediators and immune complexes reaches a critical threshold and damage occurs to tissues and cells. Unfortunately, treatment of the patient at this point is centered on limiting tissue damage. In order to prevent autoimmune disease, treatment must be given to people who have only the earliest signs or symptoms.

A challenge to early treatment in SLE is that the reliable identification of individuals at risk for organ-damaging disease is not available in clinical practice. The usual screening starts by measuring serum levels of anti-nuclear antibodies (ANAs). Since ANA positivity is essentially

required for SLE diagnosis, it is a useful filter that permits focus on the segment of the population in which SLE is likely to occur. However, the problem with ANA testing is the high prevalence of positivity, at least at low levels, in the general population, which may approach 25%[27,28]. Interpretation of ANA positivity in persons with vague or inconsistent symptoms is not straightforward and identification of the small number of individuals who are developing SLE is challenging.

Within the ANA positive referred patient population, other basic demographic and laboratory features are likely to further define higher lupus risk. One is female gender, since 85% of SLE patients are female. Another is age, since increasing age, especially after menopause in females, is associated with decreased incidence of lupus. Racial disparities also exist[29]. The presence of suggestive clinical features is another category that likely adds to SLE risk. This parallels what has been described in longitudinal studies of early synovitis in which joint complaints and symptoms such as morning stiffness and responses on visual analog measures can identify a group at risk for development of RA [30]. For lupus, this is where the ILE model is especially useful. As first described by Greer and Panush in 1989[31], these patients exhibit some features of lupus but do not satisfy sufficient criteria for classification as SLE[22,27,32-34]. A significant subset of ILE patients is likely to progress to SLE.

Given the relative safety and tolerability of hydroxychloroquine, many physicians prescribe this medication for patients who present with evidence of autoimmunity (i.e., an ANA) and some evidence of immune pathology such as a rash or joint inflammation, or even just constitutional symptoms of arthralgia and fatigue. However, the efficacy of HCQ in reducing either the objective signs or patient reported symptoms has not been tested prospectively in this population. While hydroxychloroquine is usually well tolerated, it has known side effects and its use requires regular safety monitoring. This adds risk and cost to the management of patients with a drug that has not undergone specific testing for this indication.

This study is designed to demonstrate that hydroxychloroquine can slow the progression of disease in subjects who have incomplete lupus erythematosus as measured by the accumulation of internationally defined lupus criteria.

2.1 Risk / Benefit Assessment

Systemic lupus erythematosus is a severe life-long illness that disproportionately affects young women. It can cause disfigurement, organ dysfunction, disability, and death. There is no cure, and current therapies are incompletely effective. In the last decade, numerous clinical trials of immunosuppressive or targeted biological therapies have failed to control the signs and symptoms of established lupus. In that context, a treatment that could prevent the development of SLE would be extremely beneficial. At the individual level, subjects may have reduction in their symptoms and correction of their immunological dysfunction. This study will also benefit the broader public by increasing our understanding of early autoimmunity and the mechanisms that cause some, but not all people to progress to organ- and life-threatening disease. The risks of being in the study are primarily those of taking hydroxychloroquine, a drug with a known safety profile. The investigators are all very familiar with this drug and its use in their clinical practices. Participants will be monitored for adverse effects of the drug at each visit, and global safety concerns will be reviewed by the DSMB. The most severe, but rare, adverse effect of hydroxychloroquine is eye toxicity. This will be minimized by the requirement for standard ophthalmological examinations of all participants before and after the trial. Finally, there is a

risk of receiving placebo while the participant's autoimmune disease is progressing to frank lupus. These participants will be removed from the trial if they progress and will be treated appropriately. Overall, there is reasonable prospect of benefits to participants, and risks will be carefully monitored and minimized.

3 STUDY OBJECTIVES

3.1 Primary Objective

The primary objective of this study is to assess the ability of hydroxychloroquine sulfate to prevent the development of SLE in persons at risk for the disease.

3.2 Secondary Objectives

- The effect of hydroxychloroquine on disease activity in patients with incomplete lupus erythematosus will be determined.
- The ability of hydroxychloroquine to improve patient reported outcomes related to physical functioning, anxiety, depression, fatigue, sleep disturbance, social activities, and pain will be measured.
- Changes in the levels of laboratory biomarkers including cytokines, autoantibodies, and RNA transcripts in response to therapy with hydroxychloroquine will be studied.

4 STUDY DESIGN

4.1 Study Overview

This is a multi-center, randomized, double-blind, placebo-controlled trial. The trial plans to enroll 240 subjects. Each subject will receive study drug, either hydroxychloroquine or placebo, daily for 96 weeks. Subjects will be randomized by study site and number of existing lupus criteria that they satisfy. Clinical and laboratory evaluations will take place every twelve weeks.

Screening data including history, physical, and laboratory analyses will be reviewed to determine subject eligibility. Subjects who meet all inclusion criteria and none of the exclusion criteria will be entered into the study.

The following treatment regimens will be used:

Hydroxychloroquine sulfate at 400 mg/day for subjects weighing more than 40 kilograms, and 200 mg/day for subjects weighing 40 kilograms or less.

Matching placebo – two capsules daily for subjects weighing 40 kilograms or more and one capsule/day for subjects weighing less than 40 kilograms.

Total duration of subject participation will be 104 weeks. Subjects who meet a total of four or more SLICC classification criteria for SLE during the study will stop their participation (see section 8.6) Total duration of the study is expected to be 48 months.

5 CRITERIA FOR EVALUATION

5.1 Primary Efficacy Endpoint

The primary endpoint is the increase in clinical and laboratory disease features of SLE defined by the 2012 SLICC classification criteria between week 4 and week 100.

5.2 Secondary Efficacy Endpoints

1. The proportion of participants who transition to a classification of SLE as defined by the 2012 SLICC classification criteria.
2. The proportion of participants who transition to a classification of SLE by the 1997 revised ACR classification criteria.
3. The change in Modified SLEDAI-2K score at weeks 16, 28, 40, 52, 64, 76, 88 and 100.
4. The change in CLASI cutaneous lupus scores at weeks 16, 28, 40, 52, 64, 76, 88 and 100.
5. The frequency of participants with clinically relevant autoantibodies (anti-Sm, anti-RNP, anti-Ro, and anti-La) at weeks 28, 52, 76 and 100.
6. The frequency of participants with abnormal levels of anti-DNA, C3, and C4 at weeks 28, 52, 76 and 100.
7. The increase in disease damage scores determined by the SLICC/ACR Damage Index between week 4 and week 100.
8. The proportions of participants who start, stop, or modify their use of oral or topical corticosteroids during the study.
9. Analysis of responses by race and ethnicity.

5.3 Safety Evaluations

1. Development of changes seen on ophthalmological assessment including a dilated fundoscopic examination, spectral domain ocular coherence tomography, and Humphrey visual field testing.
2. Safety as measured by physical examination, vital signs, adverse events, and laboratory tests.

5.4 Other Evaluations

1. The change in the level of IgG, IgM, and IgA autoantibodies as measured on an autoantibody array at weeks 52 and 100.
2. The change in the serum concentration of cytokines on a multiplex panel of 30 specificities at weeks 52 and 100.

6 SUBJECT SELECTION

6.1 Study Population

Female and male subjects who meet the inclusion and exclusion criteria will be recruited from a collaborative group of study sites within the United States. Typically, subjects will have presented to a health care provider for evaluation of a clinical feature consistent with partial or incomplete lupus erythematosus, such as rash or musculoskeletal complaints. Participants who meet the eligibility criteria will be enrolled without regard to gender, race or ethnicity.

6.2 Inclusion Criteria

1. Between 15 and 49 years of age, inclusive, at Visit 1. Subjects may participate in the study after their 50th birthday but Screening must occur before that date.
2. Anti-nuclear antibody (ANA) titer of 1:80, or greater, as determined by immunofluorescence assay (IFA).
3. Participants must have at least one (but not three or more) additional clinical or laboratory criterion from the 2012 Systemic Lupus International Collaborating Clinics (SLICC) classification criteria.
4. Written informed consent (and assent when applicable) obtained from subject or subject's legal representative and ability for subject to comply with the requirements of the study.

6.3 Exclusion Criteria

1. The subject meets the 2012 SLICC classification criteria for SLE at Visit 1 (i.e., ANA plus 3 other criteria, or ANA plus biopsy-proven lupus nephritis).
2. The subject has been diagnosed with another autoimmune disorder, other than autoimmune thyroid conditions.
3. The subject has fibromyalgia, based on clinical history and exam.
4. The subject has previously been or is currently being treated with oral antimalarial agents including hydroxychloroquine, chloroquine, or quinacrine.
5. The subject is currently or has been treated with immunosuppressive, immune modifying, or cytotoxic medications as listed in Section 7.2.
6. Use of any investigational agent within the preceding 12 months.
7. History of primary immunodeficiency.
8. Active bacterial, viral, fungal, or opportunistic infection.
9. Known history of infection with human immunodeficiency virus (HIV), Hepatitis B, or Hepatitis C.
10. Concomitant malignancy or history of malignancy with the exception of adequately treated basal or squamous cell carcinoma of the skin, or carcinoma in situ of the cervix.
11. The subject has significant findings on ophthalmological examination that, in the opinion of the examining Ophthalmologist, prevent safe use of hydroxychloroquine.

12. The subject has other contraindications to treatment with hydroxychloroquine including pre-existing ocular disease, hepatic impairment, psoriasis, porphyria, or allergy to the drug or class.
13. Co-morbidities requiring systemic corticosteroid therapy greater than 10 mg of prednisone, or equivalent, per day or a change in corticosteroid dose within the four weeks prior to Visit 1.
14. Pregnant, breastfeeding, or unwilling to practice birth control during participation in the study.
15. Presence of a condition or abnormality that in the opinion of the Investigator would compromise the safety of the patient or the quality of the data.
16. Inability to comply with the study visit schedule and procedures.

6.4 Premature Termination of a Participant from the Study

6.4.1 Withdrawal of Consent

Participants who withdraw their consent will be asked to complete all assessments for Visit 11 in the Schedule of Events.

6.4.2 Failure to Return

Participants who fail to return for study visits or who do not respond to repeated attempts of study staff to contact them will be considered lost to follow up.

Participants who prematurely terminate from the study prior to randomization (Visit 3) will be replaced in the study. They will not be replaced if they withdraw after randomization.

7 CONCURRENT MEDICATIONS

All subjects should be maintained on the same medications throughout the entire study period, as medically feasible, with no introduction of new chronic therapies.

7.1 Allowed Medications and Treatments

1. Use of standard (i.e., FDA approved) doses of over-the-counter or prescription non-steroidal anti-inflammatory agents (NSAIDs) is permitted.
2. Low-dose (81 mg) aspirin for cardioprotection is allowed at any time during the trial. Its use must be documented.
3. Use of oral corticosteroids equivalent to 10 mg or less of prednisone for lupus-related conditions is permitted at entry into the study as long as they were started at least 4 weeks before Visit 1, and have not changed in drug or dose. Oral corticosteroids equivalent to 10 mg or less of prednisone may be started after week 4 (baseline) for lupus-related conditions. The corticosteroid dose can be increased or decreased at the discretion of the site physician as long as the dose does not exceed 10 mg of prednisone equivalent per day for lupus-related conditions. Participants may use higher dose corticosteroids (i.e., greater than 10 mg per day of prednisone equivalent per day) to treat conditions that are not

SLICC criteria for the classification of lupus such as asthma, contact dermatitis, etc., at the discretion of the site physician.

4. Topical and/or intralesional corticosteroids to control skin disease are permitted, if subjects do not respond to study drug. Their use must be recorded at each visit.
5. Topical calcineurin inhibitors including pimecrolimus and tacrolimus to control skin disease if subjects do not respond to study drug. Their use must be recorded at each visit.
6. Subjects may receive one intra-articular corticosteroid injection to control arthritis in a single joint that does not respond to study drug during the entire 24 months of study treatment.
7. Vitamin D preparations may be used by subjects. Their use must be recorded at each visit.

7.2 Prohibited Medications and Treatments

The following medications are prohibited during the study and administration will be considered a protocol violation.

1. Oral corticosteroids in excess of 10 mg per day of prednisone, or equivalent, if used to treat lupus-related symptoms including but not limited to arthritis, serositis, or rash
2. Intramuscular or intravenous corticosteroids, if used to treat lupus-related symptoms.
3. More than one intra-articular corticosteroid injection during the entire 24 months of study treatment.
4. Methotrexate (oral or parenteral), leflunomide, sulfasalazine, azathioprine, tofacitinib, mycophenolate, thalidomide, dapsone, danazol, minocycline, oral cyclophosphamide, and penicillamine may not be used at any time before or during the study.
5. TNF inhibitors: infliximab, adalimumab, golimumab, certolizomab, and etanercept may not be used at any time before or during the study.
6. Abatacept, tocilizumab, rituximab, or belimumab may not be used at any time before or during the study.
7. Intravenous immunoglobulin (IVIG), plasmapheresis, or leukopheresis may not be used during the study.
8. Intravenous cyclophosphamide may not be used during the study.

7.3 Contraception

Although hydroxychloroquine has an excellent safety record for use in lupus and similar conditions during pregnancy, all female subjects must use a medically acceptable form of contraception during this study. Pregnancy itself may have effects on the immune system and immune function that cannot be accounted for.

8 STUDY PROCEDURES

8.1 Visit Windows

8.1.1 Scheduled Visits

A schedule of assessments by visit is given in Section 10, and the Table, "Schedule of Events," in the Appendix. The screening visit is Visit 1. Randomization will occur at Visit 3, which will occur no later than 5 weeks after Visit 1. Visits 4-11 will occur at twelve-week intervals ± 7 days.

8.1.2 Telephone Visits

At approximately one-month intervals, study personnel will call each subject to reinforce compliance with study procedures. Any potential adverse events or significant changes in the subject's medical condition will prompt an unscheduled study visit.

8.1.3 Unscheduled Visits

Unscheduled visits may be performed to document adverse events, worsening of the subject's medical condition, or withdrawal from the study. Assessments for Unscheduled Visits are given in Section 10 and the Appendix.

8.2 Enrollment, Randomization and Blinding

8.2.1 Enrollment

Participants who meet the eligibility criteria in Section 6 will be enrolled at study Visit 1. Prior to that time, the participants will have had a copy of the informed consent document to review and will have had an opportunity to ask questions of the study team. Informed Consent and Authorization for Disclosure under HIPAA will be documented before any study procedures take place.

8.2.2 Randomization

Participants will be randomly assigned to receive hydroxychloroquine or matching placebo at Visit 3 (week 4) using a centralized system that is stratified by treatment site and number of baseline SLICC classification criteria. That is, ANA plus one criterion, or ANA plus two criteria. Randomization will be done with varying permutation block sizes at each IDS pharmacy to minimize inadvertent un-blinding of treatment group assignment.

8.2.3 Blinding

Participants and the clinical study staff will be blinded as to which treatment is administered. Study pharmacists at each site (if required) will not be blinded. Other personnel (study monitors, statisticians, NIAMS project officers, etc.) will remain blinded to study treatment until all subjects have completed the trial and the database is locked.

Every effort will be undertaken to maintain the blinding of study treatment. In the event that a participant has a medical emergency the site principal investigator may break the blind by contacting their local IDS pharmacy. This should be done only if it will affect the participant's immediate medical care and should be discussed with the Medical Monitor and Sponsor.

Information for un-blinding at each site will be kept in a printed document that will be stored in a secure location within the local IDS pharmacy and will be used by the site investigator with permission from the Sponsor.

8.3 Study Treatments

8.3.1 Hydroxychloroquine sulfate

Hydroxychloroquine tablets will be purchased from commercial generic pharmaceutical vendors that have been approved by the US Food and Drug Administration for sale in the United States. The University of Iowa Pharmaceuticals (UIP) will over-encapsulate 200 mg hydroxychloroquine tablets, filling the capsules with microcrystalline cellulose.

8.3.2 Placebo

Placebo capsules consisting of microcrystalline cellulose and identical in size and shape to the hydroxychloroquine capsules will be manufactured by UIP.

8.3.3 Packaging and Labeling

Study drug, either hydroxychloroquine or matching placebo, will be packaged by UIP in 150 cc HDPE Drug Plastics 38/400 round, white bottles with child-resistant induction sealed closures. The bottles will be packaged into kits, each containing two bottles by the University of Rochester Clinical Materials Services Unit (CMSU).

Each kit and the two included bottles will be labeled with the protocol name/number, the participant's randomization number, the name of the sponsor, and the directions for participant use and storage.

8.4 Supply of Study Drug at the Site

CMSU will ship the study drug to the investigational sites in batches of up to sixty (60) kits per shipment. The initial study drug shipment will be shipped after site activation (i.e., all required regulatory documentation has been received by the Sponsor and a contract (NIH sub-award) has been executed). Subsequent study drug shipments will be made after site request to the Sponsor for resupply.

8.4.1 Storage

Study drug should be stored by the study site at controlled room temperature, 15 to 30ºC (59 to 86ºF). If the temperature of study drug storage in the clinic/pharmacy exceeds or falls below this range, this should be reported to the Sponsor or designee and captured as a deviation. Subjects will be instructed to store the medication in original packaging (plastic bottles) at room temperature.

8.4.2 Dosage/Dosage Regimen

Hydroxychloroquine will be dosed as follows: Participants weighing more than 40 kilograms at the time of randomization (Visit 3) will be given 400 mg per day. Participants weighing 40 kilograms or less at the time of randomization (Visit 3) will be given 200 mg per day. Participant weight will be verified at each subsequent visit and the dose adjusted if necessary. The blinded drug capsules contain 200 mg of hydroxychloroquine or placebo. Thus, the dosing

regimen will be 2 capsules once a day for participants >40 kilograms and one capsule once a day for participants ≤40 kilograms.

8.4.3 Dispensing

Study drug will be dispensed once the site investigator or sub-investigator has certified that they meet all eligibility requirements, and they have had a satisfactory ophthalmological evaluation. The local IDS pharmacist will randomize the participants using the REDCap system. The pharmacy will dispense the appropriate treatment to the research coordinator. The dispensation of the study drug will be documented on a log in the pharmacy as well as on a medication dispensation form at the clinic. The study coordinator will verify the labeling on the kit and both bottles before giving them to the participant along with instructions for use of the study drug.

8.5 Study Drug Accountability

Under federal regulations (21CFR 312.62) an investigator is required to maintain adequate records of the disposition of the investigational product, including the date and quantity of drug that was received, the participants to whom drug was dispensed (participant by participant accounting), and an account of any drug accidentally or deliberately destroyed.

Records for receipt, storage, use, and disposition of the study drug will be maintained by the study sites. A drug-dispensing log will be kept current for each participant and will contain the identification of each participant and the date and quantity of drug dispensed.

All records regarding disposition of the investigational product will be available for inspection by the clinical trial monitor.

8.6 Discontinuation of Study Drug

Participants **must** discontinue the study drug (hydroxychloroquine sulfate or placebo) if any of the following occurs:

- The participant develops a total of four or more SLICC criteria for the classification of SLE
- The participant withdraws consent or wishes to stop study treatment
- The investigator determines it is in the best interest of the participant to discontinue treatment due to intercurrent illness (including ILE), laboratory abnormality, clinical adverse event, or non-compliance with study procedures
- The participant becomes pregnant
- The use of prohibited medications including doses of corticosteroids greater than the equivalent of 10 mg/d of prednisone or IM or IV steroids, when used to treat lupus-related symptoms.
- Un-blinding of study medication, either intentional or un-intentional

After discontinuation of study drug, any needed medical care should be provided at the discretion and practice of the principal investigator or the patient’s personal physician.

The Medical Monitor and Sponsor must be notified of any decision to discontinue study drug

If the study treatment is discontinued at a regularly scheduled visit, the participant should complete all the assessments and laboratory testing for that visit. If the study drug is discontinued between scheduled visits (e.g., due to an adverse event), the participant should complete the assessments for Visit 11 unless they have withdrawn consent to participate.

9 STUDY ASSESSMENTS AND GUIDELINES

A Schedule of Events representing the required testing procedures to be performed for the duration of the study is diagrammed in Appendix 1.

Prior to conducting any study-related activities, written informed consent and the Health Insurance Portability and Accountability Act (HIPAA) authorization must be signed and dated by the subject or subject's legal representative. If appropriate, assent of minor participants must also be obtained prior to conducting any study-related activities.

9.1 Clinical Assessments

9.1.1 Concomitant Medications

All concomitant medication and concurrent therapies will be documented at Visit 1 (screening) and all subsequent visits. Dose, route, frequency of administration, and indication for administration and dates of medication will be captured.

9.1.2 Demographics

Demographic information (date of birth, gender, race and ethnicity) will be recorded at Visit 1.

9.1.3 Medical and Family History

Relevant medical history, including features of incomplete lupus erythematosus, and other pertinent medical conditions, and their treatment will be recorded at Visit 1. A history of autoimmune disease in the participant's biological parents, biological siblings, and biological children will be elicited and recorded without direct identifiers.

9.1.4 Physical Examination

Either the investigator or a sub-investigator who is a physician at Visits 1 and 3-11 will perform a complete physical examination. New abnormal physical exam findings must be documented and will be followed by a physician or other qualified staff at the next scheduled visit.

9.1.5 Vital Signs

Body temperature, blood pressure, and pulse will be performed after resting for 5 minutes at all visits.

9.1.6 Other Clinical Procedures

9.1.6.1 Ophthalmological Screening for Retinal Pathology

At Visit 2, and again at Visit 12, participants will undergo ophthalmological examination including:

1. Dilated fundoscopic examination (DFE)

2. Spectral domain ocular coherence tomography (SD-OCT)
3. Humphrey visual field (HVF) testing using a 10-2 pattern

9.1.6.2 2012 SLICC Criteria for the Classification of Lupus

At each visit, the presence or absence of clinical classification criteria for SLE set forth by the Systemic Lupus International Collaborating Clinics (SLICC) (see Appendix) will be determined and recorded.

9.1.6.3 1997 ACR Criteria for the Classification of Lupus

At each visit, the presence or absence of clinical classification criteria for SLE set forth by the American College of Rheumatology (ACR) (see Appendix) will be determined and recorded.

9.1.6.4 Modified SLEDAI-2K

At visits 3 through 11, the Modified SLEDAI-2K disease activity instrument for SLE will be determined and recorded. The Modified SLEDAI-2K is identical to the original SLEDAI-2K, but includes the provider's global assessment (PGA) and SLE Flare Index incorporated into the document. The period of assessment is the 30 days prior the visit as this has been effective in longitudinal studies[35].

9.1.6.5 CLASI

At visits 3 through 11, the Cutaneous Lupus Disease Area and Severity Index (CLASI) will be determined and recorded

9.1.6.6 SLICC/ACR Damage Index

At Visit 3 (Baseline), Visit 7 (Week 52) and Visit 11 (Week 100), the SLICC/ACR Damage Index will be determined and recorded.

9.1.6.7 Patient Reported Outcomes

At each visit, patient reported outcomes instruments will be administered and recorded. These include the PROMIS 29 Adult or Pediatric Profile supplemented with additional questions from the PROMIS Fatigue item bank, and a Patient Global Visual Analogue Scale.

9.1.6.8 Connective Tissue Disease Screening Questionnaire (CSQ)

At Visit 1 (Screening) the CSQ will be administered and recorded.

9.1.7 Adverse Events

Information regarding occurrence of adverse events will be captured throughout the study. Duration (start and stop dates), severity/grade, outcome, treatment and relation to study drug will be recorded on the case report form (CRF).

9.2 Clinical Laboratory Measurements

The following tests (9.3 – 9.4.6) will be performed as outlined in Section 10 and the Schedule of Events (Appendix 1)

9.3 Hematology

Blood will be obtained and sent to each site's clinical hematology lab for a complete blood count (hemoglobin, hematocrit, red blood cell count, white blood cell count, white blood cell differential, and platelet count), and direct anti-globulin (Coombs) test.

9.4 Blood Chemistry

Blood will be obtained and sent to each site's clinical chemistry lab for determination of serum sodium, potassium, chloride, bicarbonate, random glucose, BUN, creatinine, aspartate aminotransferase (AST/SGOT), alanine aminotransferase (ALT/SGPT), alkaline phosphatase, calcium, total bilirubin, total protein, and albumin.

9.4.1 Glucose-6-phosphate dehydrogenase (G6PD)

Blood will be obtained and sent to each site's clinical lab for determination of the blood level of glucose-6-phosphate dehydrogenase.

9.4.2 Vitamin D

Blood will be obtained and sent to each site's clinical chemistry lab for determination of the serum concentration of 25-OH-vitamin D.

9.4.3 Pregnancy Test

A urine pregnancy test will be obtained from female subjects who are of childbearing age prior to their participation in the study, at week 52 and week 100.

9.4.4 Urinalysis

Urine will be obtained and sent to each site's clinical laboratory for determination of color, specific gravity, pH, protein (qualitative and quantitative), glucose, ketones, blood and creatinine (quantitative). Microscopic analysis will be performed to determine the presence of casts and cellular elements.

9.4.5 Complement

Blood will be obtained and sent to each site's clinical chemistry lab for determination of complement 3 (C3) and complement 4 (C4) concentrations.

9.4.6 Clinical Autoantibodies

Blood will be obtained and sent to a central laboratory for determination of anti-nuclear antibodies by immunofluorescence, anti-CCP 3.1, antibodies to double-stranded DNA, Ro60, Ro52, La, Sm, U1-RNP, Jo-1, centromere, histones, cardiolipin, and beta-2-glycoprotein I.

9.5 Planned Mechanistic Assays

The following studies will be performed as outlined in Section 10 and the Schedule of Events (Appendix).

9.5.1 Autoantigen Array

Blood will be obtained and sent to a central lab for determination of serum/plasma IgG, IgM, and IgA autoantibodies to a panel of ~100 auto-antigens using microarray methodology.

Serum levels of specific antibodies will be compared between baseline and different time points. Levels of antibodies will be compared to the presence of clinical features of SLE. The presence and concentration of antibodies will be correlated with treatment.

9.5.2 Serum Cytokine Measurements

Blood will be obtained and sent to a central lab for determination of serum/plasma concentrations of ~30 cytokines and inflammatory mediators using multiplex bead technology.

Serum levels of specific cytokines/mediators will be compared between baseline and different time points. Levels of cytokines will be compared to the presence of clinical features of SLE. The presence and concentration of cytokines will be correlated with treatment.

9.6 Future/Unplanned Studies

Samples of serum, plasma, DNA, RNA from peripheral blood mononuclear cells, and urine will be obtained from participants at the visits indicated in Section 10 and the Schedule of Events. These samples will be banked and used for exploratory analyses outside the scope of this clinical trial. It is likely that both the investigators of this trial, as well as other investigators, will perform these studies. Re-evaluations or new assays will only be carried out on samples from participants who have given their consent for such future studies and those studies have received appropriate approval from an Institutional Review Board. The sample sharing policies of NIAMS will apply to these specimens.

9.7 Specimen Logistics

All specimens collected from patients will be barcoded according to the Subject ID obtained from the Interactive Web-Based Randomization Service. A SMILE project sample collection sheet will be provided to sites to fill out, with the attached subject ID barcode. This indicates both the number of sample collection tubes specified in the protocol and the number of each tube type collected and shipped to the OMRF central processing/biorespository.

9.8 Specimen Tracking Procedures

Upon shipment of specimens, the clinical site coordinator will enter the information about the shipment, including the Federal Express tracking number and number of subjects contained in the shipment into the REDCap system. An email alert will automatically be sent to the OMRF specimen processing unit. All shipments will be sent under ambient temperature conditions. Shipments will be packaged and labeled according to IATA approved clinical specimen shipment requirements and will contain a copy of the sample collection sheet for each subject included in that shipment. Shipments will only occur on Monday-Thursday, to be received Tuesday-Friday. No weekend or holiday shipments will be allowed.

Upon receipt of samples, the OMRF Biorepository staff will record the information about the specimens in the OMRF Autoimmune Disease Institute Data System (ADIDS) to track receipt, processing, storage and distribution of derivatives from the SMILE project. The OMRF Biorepository acts in the capacity as an honest broker for samples, only processing storing and

distributing samples to OMRF central laboratories or to UT Southwestern autoantibody Core facility as directed by this protocol, or stored for future studies as approved by the SMILE Executive Committee and appropriate Institutional Review Boards.

All sample processing will occur according to the standard operating procedures for SMILE samples, including collection and generation of the appropriate derivative aliquots and storage according to the specified project protocol.

9.9 Specimen Storage

The OMRF Biorepository stores ambient, 4° C, -20° C, -80° C or -196° C (Liquid nitrogen vapor phase) samples in highly secured area only accessible by OMRF Biorepository Staff, that is monitored and has redundantly backed up informatics systems. Business continuity plans are in place for all types of emergency or unexpected situations. All cryostorage systems are monitored 24/7 with multiple levels of notification and verification procedures when deviations in temperatures outside of normal limits occur. All systems are logged and validated by NIST certified temperature-mapping systems once each year.

Storage locations of each derivative aliquot are tracked in the ADIDS system and every usage is noted, integrity updated and QC parameters assessed prior to distribution to the OMRF and UT Southwestern laboratories, as well as users who have requested and been approved for samples from the SMILE project.

10 EVALUATIONS BY VISIT

10.1.1 Visit 1 (Week 0)

1. Review the study with the subject (or subject's legal representative) and obtain written informed consent and HIPAA authorization. Subjects under the age of 18 will be required to give assent.
2. Assign the subject a unique screening number provided by the PSU Data Center.
3. Record demographics data.
4. Record medical history, including a history of incomplete lupus erythematosus, diagnosis date, and prior medical treatments.
5. Record concomitant medications.
6. Perform a complete physical examination.
7. Perform and record vital signs.
8. Collect blood and urine for clinical laboratory tests (chemistry, hematology, urine pregnancy test, urinalysis with microscopic examination, urine protein and creatinine concentration, complement 3 and complement 4 concentrations, direct anti-globulin test, G6PD level, and 25-OH-vitamin D) and, anti-CCP3.1, and clinical autoantibodies.
9. Administer the Connective Tissue Disease Screening Questionnaire (CSQ)
10. If the participant meets eligibility criteria, schedule ophthalmological evaluation.

10.2 Visit 2 and 12 (Week 1-5 and week 100-104)

1. Obtain Ophthalmological screening for retinal pathology

2. If the initial ophthalmological examination is satisfactory, schedule the participant for Visit 3
3. In order to facilitate participant and site logistics, Visit 2 can occur at any time after Visit 1, including the same day, and any time before Visit 3, including the same day. However, no participant can be randomized to study medication without a satisfactory ophthalmological evaluation.

10.3 Visit 3 (Up to 5 weeks after Visit 1)

1. Confirm eligibility criteria based on laboratory data from Visit 1.
2. Record any Adverse Events.
3. Concomitant medications review.
4. Perform physical examination.
5. Perform and record vital signs.
6. Perform assessment for SLICC and ACR Classification Criteria for SLE
7. Perform assessment for Modified SLEDAI-2K and CLASI instruments
8. Perform and SLICC/ACR Damage Index assessments
9. Administer PROMIS, and Patient Global Assessment patient reported outcomes assessments.
10. Collect blood and urine for clinical laboratory tests (hematology, urinalysis with microscopic examination, urine protein concentration, anti-double stranded DNA antibodies by BioPlex, antibodies to extractable nuclear antigens by BioPlex, complement 3 and complement 4 concentrations).
11. Collect blood for multiplex cytokine measurement
12. Collect blood for autoantibody array measurement
13. Collect blood for DNA, RNA, serum, plasma biobanking
14. Collect urine for biobanking.
15. Randomize subject at each site by study coordinator.
16. Dispense study medication.

10.4 Visits 4, 5, 6, and 8, 9, 10 (Weeks 16, 28, 40, and weeks 64, 76, and 88 ± 7 days)

1. Record any Adverse Events.
2. Concomitant medications review.
3. Perform physical examination.
4. Perform and record vital signs.
5. Perform assessment for SLICC and ACR Classification Criteria for SLE
6. Perform assessment for Modified SLEDAI-2K and CLASI instruments.

7. Administer PROMIS, and Patient Global Assessment patient reported outcomes assessments.
8. Collect blood and urine for clinical laboratory tests (hematology, urinalysis with microscopic examination, urine protein and creatinine concentration, anti-double stranded DNA antibodies by BioPlex, antibodies to extractable nuclear antigens by BioPlex, complement 3 and complement 4 concentrations)
9. Collect blood for multiplex cytokine measurement
10. Collect blood for autoantibody array measurement
11. Collect blood for RNA, serum, plasma biobanking
12. Collect urine for biobanking
13. Record medication compliance and dispense study medication.

10.5 Visits 7 and 11 (Weeks 52 and 100 ± 7 days)

1. Record any Adverse Events.
2. Concomitant medications review.
3. Perform physical examination.
4. Perform and record vital signs.
5. Perform assessment for SLICC and ACR Classification Criteria for SLE
6. Perform Modified SLEDAI-2K and CLASI instruments.
7. Perform SLICC/ACR Damage Index assessments
8. Administer PROMIS, and Patient Global Assessment patient reported outcomes assessments.
9. Collect blood and urine for clinical laboratory tests (chemistry, hematology, urine pregnancy test, urinalysis with microscopic examination, urine protein and creatinine concentration, complement 3 and complement 4 concentrations, direct anti-globulin test).
10. Collect blood for multiplex cytokine measurement
11. Collect blood for autoantibody array measurement
12. Collect blood for RNA, serum, plasma biobanking
13. Collect urine for biobanking.
14. Record medication compliance (both Visits) and dispense study medication (Visit 7 only).

10.6 Unscheduled Visit / Early Withdrawal Visit

1. Record any Adverse Events.
2. Concomitant medications review.
3. Perform physical examination.

4. Perform and record vital signs.
5. Perform assessment for SLICC and ACR Classification Criteria for SLE. **Note:** Only the clinical assessments will be done at an Unscheduled Visit to determine whether the participant has a finding that would lead to study withdrawal, i.e., progression to SLE classification. Laboratory assessments will only be done if medically necessary.
6. Perform Modified SLEDAI-2K and CLASI instruments only if the participant is withdrawing from the study.
7. Perform SLICC/ACR Damage Index assessments only if the participant is withdrawing from the study.
8. Administer PROMIS, and Patient Global Assessment patient reported outcomes assessments only if the participant is withdrawing from the study.
9. Collect blood and urine for clinical laboratory tests (chemistry, hematology, urinalysis with microscopic examination, urine protein and creatinine concentration, complement 3 and complement 4 concentrations, direct anti-globulin test) only if the participant is withdrawing from the study.
10. Collect blood for DNA, RNA, serum, plasma biobanking only if the participant is withdrawing from the study.
11. Collect urine for biobanking only if the participant is withdrawing from the study.
12. Collect blood for multiplex cytokine measurement only if the participant is withdrawing from the study.
13. Collect blood for autoantibody array measurement only if the participant is withdrawing from the study.
14. Record medication compliance in all participants.

11 ADVERSE EVENT DOCUMENTATION AND REPORTING

11.1 Adverse Events

An adverse event (AE) is any untoward medical occurrence in a clinical investigation of a patient administered a pharmaceutical product and that does not necessarily have a causal relationship with the treatment. An AE is therefore any unfavorable and unintended sign (including an abnormal laboratory finding), symptom or disease temporally associated with the administration of an investigational product, whether or not related to that investigational product. An unexpected AE is one of a type not identified in nature, severity, or frequency in the current Investigator's Brochure or of greater severity or frequency than expected based on the information in the Investigator's Brochure.

The Investigator will probe, via discussion with the subject, for the occurrence of AEs during each subject visit and record the information in the site's source documents. Adverse events will be recorded in the patient CRF. Adverse events will be described by duration (start and stop dates and times), severity, outcome, treatment and relation to study drug, or if unrelated, the cause.

AE Severity

The National Cancer Institute's Common Terminology Criteria for Adverse Events (CTCAE) Version 4.03 (June 14, 2010), should be used to assess and grade AE severity, including laboratory abnormalities judged to be clinically significant. The modified criteria can be found in the study manual. If the event is not covered in the modified criteria, the guidelines shown in Table 1 below should be used to grade severity. It should be pointed out that the term "severe" is a measure of intensity and that a severe AE is not necessarily serious.

Table 1. AE Severity Grading

Severity (Toxicity Grade)	Description
Mild (1)	Transient or mild discomfort; no limitation in activity; no medical intervention or therapy required. The subject may be aware of the sign or symptom but tolerates it reasonably well.
Moderate (2)	Mild to moderate limitation in activity, no or minimal medical intervention/therapy required.
Severe (3)	Marked limitation in activity, medical intervention/therapy required, hospitalizations possible.
Life-threatening (4)	The subject is at risk of death due to the adverse event as it occurred. This does not refer to an event that hypothetically might have caused death if it were more severe.
Death (5)	The subject has died as a direct result of the adverse event.

AE Relationship to Study Drug

The relationship of an AE to the study drug should be assessed using the following the guidelines in Table 2.

Table 2. AE Relationship to Study Drug

Relationship to Drug	Comment
Definitely (3)	Previously known toxicity of agent; or an event that follows a reasonable temporal sequence from administration of the drug; that follows a known or expected response pattern to the suspected drug; that is confirmed by stopping or reducing the dosage of the drug; and that is not explained by any other reasonable hypothesis.
Probably (2)	An event that follows a reasonable temporal sequence from administration of the drug; that follows a known or expected response pattern to the suspected drug; that is confirmed by stopping or reducing the dosage of the drug; and that is unlikely to be explained by the known characteristics of the subject's clinical state or by other interventions.
Possibly (1)	An event that follows a reasonable temporal sequence from administration of the drug; that follows a known or expected response pattern to that suspected drug; but that could readily have been produced by a number of other factors.
Unrelated (0)	An event that can be determined with certainty to have no relationship to the study drug.

11.2 Serious Adverse Events (SAE)

An SAE is defined as any AE occurring at any dose that results in any of the following outcomes:

- Death
- A life-threatening adverse event
- Inpatient hospitalization or prolongation of existing hospitalization
- A persistent or significant disability/incapacity
- A congenital anomaly/birth defect

Other important medical events may also be considered an SAE when, based on appropriate medical judgment, they jeopardize the subject or require intervention to prevent one of the outcomes listed.

11.2.1 Serious Adverse Event Reporting

Study sites will document all SAEs that occur (whether or not related to study drug) on an SAE Report Form. The collection period for all SAEs will begin after informed consent is obtained and end after procedures for the final study visit have been completed.

All SAE Report Forms will be reviewed by the site investigator and sent to the Protocol Chair for Regulatory Affairs (Dr. David Karp) within one business day of the site learning of the event. Sites will fax the SAE report to:

- David R. Karp, MD, PhD SAE Fax: (214) 648-7995

Dr. Karp will notify Dr. Nancy Olsen, Sponsor and Protocol Chair, as well as the Medical Monitor and the Independent Safety Officer, Extramural Program, NIAMS/NIH of the SAE.

The site will notify Dr. Karp of additional information or follow-up to an initial SAE Report as soon as relevant information is available. Follow-up information is reported on an SAE Report Form.

In accordance with the standard operating procedures and policies of the local Institutional Review Board (IRB)/Independent Ethics Committee (IEC), the site investigator will report SAEs to the IRB/IEC.

The NIAMS Independent Safety Officer will provide the DSMB with data on all SAEs on an ongoing basis, including quarterly reports of all SAEs.

11.3 Medical Monitoring

Joan T. Merrill, MD should be contacted directly at these numbers to report medical concerns or questions regarding safety.

Phone: 405-271-7805
24 hour answering Service: 405-330-4471

12 DISCONTINUATION AND REPLACEMENT OF SUBJECTS

12.1 Early Discontinuation of Study Drug

A subject may be discontinued from study treatment at any time if the subject, the investigator, or the Sponsor feels that it is not in the subject's best interest to continue. The following is a list of possible reasons for study treatment discontinuation:

1. Subject withdrawal of consent (or assent)
2. Subject is not compliant with study procedures
3. Adverse event that in the opinion of the investigator would be in the best interest of the subject to discontinue study treatment
4. Protocol violation requiring discontinuation of study treatment
5. Lost to follow-up
6. Sponsor request for early termination of study
7. Positive pregnancy test (females)
8. The subject can be classified as having SLE by the 2012 SLICC classification criteria

If a subject is withdrawn from treatment due to an adverse event, the subject will be followed and treated by the Investigator until the abnormal parameter or symptom has resolved or stabilized.

All subjects who discontinue study treatment should come in for an early discontinuation visit as soon as possible and then should be encouraged to complete all remaining scheduled visits and procedures.

All subjects are free to withdraw from participation at any time, for any reason, specified or unspecified, and without prejudice.

Reasonable attempts will be made by the investigator to provide a reason for subject withdrawals. The reason for the subject's withdrawal from the study will be specified in the subject's source documents Refer to Section 10.6 for early termination procedures.

12.3 Withdrawal of Subjects from the Study

A subject may be withdrawn from the study at any time if the subject, the investigator, or the Sponsor feels that it is not in the subject's best interest to continue.

All subjects are free to withdraw from participation at any time, for any reason, specified or unspecified, and without prejudice.

Reasonable attempts will be made by the investigator to provide a reason for subject withdrawals. The reason for the subject's withdrawal from the study will be specified in the subject's source documents. As noted above, subjects who discontinue study treatment early (i.e., they withdraw prior to Visit 10) should have an early discontinuation visit. Refer to Section 10.6 for early termination procedures. Subjects who withdraw after Visit 3 but prior to Visit 11 should be encouraged to come in for a final visit (and the procedures to be followed would include those for their next scheduled visit).

12.4 Replacement of Subjects

Subjects who withdraw from the study treatment will not be replaced if they have received at least one dose of study drug.

Subjects who withdraw from the study prior to randomization (Visit 3) be replaced.

13 PROTOCOL VIOLATIONS

A protocol violation occurs when the subject, investigator, or Sponsor fails to adhere to significant protocol requirements affecting the inclusion, exclusion, subject safety and primary endpoint criteria. Protocol violations for this study include, but are not limited to, the following:

1. Failure to meet inclusion/exclusion criteria
2. Use of a prohibited concomitant medication
3. Non-compliance with study drug regimen
4. Failure to return for study visits within the prescribed windows
5. Failure to comply with Good Clinical Practice (GCP) guidelines will also result in a protocol violation. The Sponsor will determine if a protocol violation will result in withdrawal of a subject.

When a protocol violation occurs, it will be discussed with the investigator and a Protocol Violation Form detailing the violation will be generated. This form will be signed by a Sponsor representative and the Investigator. A copy of the form will be filed in the site’s regulatory binder and in the Sponsor’s files.

14 DATA SAFETY MONITORING

The NIAMS will appoint an Independent Safety Officer and establish a Data Safety Monitoring Board (DSMB) to review data relating to safety and efficacy, to conduct and review interim analyses, and to ensure the continued scientific validity and merit of the study, according to the NIAMS Data Safety Monitoring Board Operations Manual and a DSMB Charter to be established for this protocol. Interim reviews will be conducted by the DSMB for the purpose of monitoring study conduct and assessing patient safety on a twice-yearly basis. Further details regarding the timing and content of the interim reviews are included in the statistical section below.

15 STATISTICAL METHODS AND CONSIDERATIONS

Prior to the analysis of the final study data, a detailed Statistical Analysis Plan (SAP) will be written describing all analyses that will be performed. The SAP will contain any modifications to the analysis plan described below.

15.1 Data Sets Analyzed

All participants who are randomized and receive at least one dose of study medication will be included in the efficacy and safety data sets (intention to treat).

All data from participants who receive any study treatment and for whom there is a baseline (Visit 3) and at least one other biological sample available will be included in the data set for mechanistic (proteomic) analyses.

15.2 Demographic and Baseline Characteristics

The following baseline and demographic variables will be summarized by randomization to placebo or hydroxychloroquine:

- Gender
- Age
- Ethnicity
- Race
- Number and type of SLICC classification criteria met
- Duration of symptoms
- Titer of antinuclear antibody
- Other clinical autoantibodies present
- Past medical history of autoimmune disease
- Family history of autoimmune disease
- Medical treatments including topical or systemic corticosteroids.

15.3 Analysis of Primary Endpoint

Randomization or participants will be stratified according to clinical site and number of SLICC criteria at baseline. That is, ANA plus one additional criterion (two total) or ANA plus two additional criteria (three total). The primary outcome variable in this double blind, randomized trial is the SLICC classification criteria count, measured every 12 weeks over a 96-week period. Patients are eligible for the trial if they exhibit a SLICC classification criteria count of 2 or 3 at the baseline visit. Ordinal logistic regression analysis with random effects will be applied to compare the estimated slopes for the HCQ and placebo groups with respect to the SLICC classification criteria count over the 96-week follow-up period[36]. The primary null hypothesis is that the HCQ slope equals the placebo slope, and its corresponding alternative hypothesis is that the HCQ slope does not equal the placebo slope. Thus, a two-sided test with a 0.05 significance level will be applied. The statistical model will account for the ordinal and non-decreasing properties of the SLICC classification criteria count during the 96-week follow-up period, as well as account for the repeated measurements on each patient. This statistical model will form the basis of the primary statistical analysis of the SLICC classification criteria count, as well as all secondary and subgroup analyses with the SLICC classification criteria count.

A maximum likelihood (ML) estimation will be applied for estimating all of the model parameters, namely, the intercepts, the fixed-effects parameter vector, and the variance-covariance matrix for the random effects, and apply likelihood ratio (LR) tests for testing hypotheses. Finally, a sensitivity analysis will be conducted by constructing a cell means model

(a mean parameter for each time point and each treatment group, instead of a slope model) and comparing the 96-week change between the HCQ and placebo groups.

The ML estimation and LR tests are valid under a missing-at-random (MAR) mechanism. If there is reason to suspect non-ignorable missing data mechanism, then the statistical model will be expanded to be a joint model that includes (1) the ordinal logistic regression model of the SLICC repeated measurements, (2) a hazards regression model for the time-to-withdrawal, and (3) shared fixed-effects and random-effects parameters between the two models[37,38].

The following statistical principles will guide the development of SAP for Primary Outcome:

For simplicity, we present the statistical models as if each participant will terminate the study at the 96-week visit. In actuality, however, we will target a follow-up period of 96 weeks for each participant but follow each participant as long as possible. Let Y_{ijk} denote the SLICC score for the kth time point (k = 0, 1, … , 8, corresponding to 0, 12, … , 96 weeks) of the jth patient (j = 1, 2, … , n_i) within the ith clinical site (i = 1, 2, 3, 4, 5). We define a set of three conditional cumulative probabilities for follow-up visits (k = 1, 2, … , 8) as

$$p_{ijk,3|2} = \Pr[Y_{ijk} = 3, 4 \mid Y_{ij,k-1} = 2] \qquad p_{ijk,4|2} = \Pr[Y_{ijk} = 4 \mid Y_{ij,k-1} = 2]$$

$$p_{ijk,4|3} = \Pr[Y_{ijk} = 4 \mid Y_{ij,k-1} = 3]$$

and we construct a corresponding set of three conditional cumulative logits for follow-up visits (k = 1, 2, … ,8) as

$$\mu_{ijk,3|2} = \log_e\{p_{ijk,3|2}/(1 - p_{ijk,3|2})\} \qquad \mu_{ijk,4|2} = \log_e\{p_{ijk,4|2}/(1 - p_{ijk,4|2})\}$$

$$\mu_{ijk,4|3} = \log_e\{p_{ijk,4|3}/(1 - p_{ijk,4|3})\}$$

Finally, we model the three conditional cumulative logits in the following manner:

$$\mu_{ijk,3|2} = \alpha_{i,3|2} + x_{ijk}^T\beta + z_{ijk}^T\delta_{ij} \qquad \mu_{ijk,4|2} = \alpha_{i,4|2} + x_{ijk}^T\beta + z_{ijk}^T\delta_{ij}$$

$$\mu_{ijk,4|3} = \alpha_{i,4|3} + x_{ijk}^T\beta + z_{ijk}^T\delta_{ij}$$

where:

- $\alpha_{i,3|2}$, $\alpha_{i,4|2}$, and $\alpha_{i,4|3}$ are intercept parameters for the three logits within the ith clinical site
- x_{ijk} is a vector of regressors for the fixed-effects parameter vector at the kth time point of the jth patient within the ith clinical site
- β is a fixed-effects parameter vector
- z_{ijk} is a vector of regressors for the random-effects parameter vector at the kth time point of the jth patient within the ith clinical site

- δ_{ij} is a random-effects parameter vector for the jth patient within the ith clinical site, and we assume that the δ_{ij}'s are independent and identically distributed according to a multivariate normal distribution with a null mean vector and an unstructured, positive-definite, variance-covariance matrix Δ

For the primary statistical analysis of the SLICC scores measured during the 96-week follow-up period,

- $x_{ijk}^T\beta = k \cdot \beta_{HCQ}$ if the patient is randomized to the HCQ group and $x_{ijk}^T\beta = k \cdot \beta_{placebo}$ if the patient is randomized to the placebo group, where k represents the time point (k = 1, 2, … , 8) and β_{HCQ} and $\beta_{placebo}$ represent the fixed-effect slopes for the HCQ and placebo groups, respectively
- $z_{ijk}^T\delta_{ij} = k \cdot \delta_{ij}$, where δ_{ij} is univariate

For secondary and subgroup analyses, the vector of fixed-effects regressors, x_{ijk}, and the vector of random-effects regressors, z_{ijk}, will include baseline, demographic, and co-morbidity variables.

15.4 Analysis of Secondary Endpoints

The following statistical principles will guide the development of SAP for secondary outcomes:

The important secondary outcomes variables in this 96-week trial are **(1)** the time to progression from ILE to clinical SLE; **(2)** disease activity as measured by SLEDAI and CLASI scores and patient-reported outcomes as measured by PROMIS questionnaires at each visit; **(3)** concentrations of at least 50 soluble mediators and the number of positive autoantibodies (out of approximately 100 autoantibodies) detected; and **(4)** the incidences of HCQ-related ophthalmologic toxicity. Outcome variables in items **(2)** and **(3)** above are measured at the baseline and at each follow-up visit, and can be treated as continuous variables except for the proportion of positive antibodies will be regarded as having a binomial distribution. Safety variables in item **(4)** above are measured twice, baseline and at the end of the treatment period as binary variables.

15.4.1 Statistical Analysis for the time to progression from ILE to SLE

Because the time to progression from ILE to clinical SLE will be assessed at each follow-up visit, it is interval-censored. Therefore, we will apply a discrete time-to-event hazard model[39]. In effect, the discrete time-to-event hazard model reduces to a logistic regression model with a complementary log-log link function. Let $Y_{ijk} = 0$ or 1 according to whether the progression event is not, or is, detected at the kth time point (k = 0, 1, … , 8, corresponding to 0, 12, … , 96 weeks) of the jth patient (j = 1, 2, … , n_i) within the ith clinical site (i = 1, 2, 3, 4, 5). Next, let $p_{ijk} = \Pr[Y_{ijk} = 1]$ and denote its log-log link function as $\mu_{ijk} = \log_e[-\log_e\{1 - \Pr(Y_{ijk} = 1)\}]$. Then the statistical model is

$$\mu_{ijk} = \alpha_{ik} + x_{ij}^T\beta$$

where

- α_{ik} is the intercept parameter for the kth time point within the ith clinical site
- x_{ij} is a vector of regressors for the jth patient within the ith clinical site
- β is a fixed-effects parameter vector

The vector of regressors, x_{ij}, will consist of an indicator function for treatment group assignment (HCQ or placebo) and will include baseline, demographic, and co-morbidity variables for secondary and subgroup analyses. We will apply ML estimation for estimating all of the model parameters, and apply LR tests for testing hypotheses, via SAS proc logistic, Version 9.4.

15.4.2 Statistical Analysis for the continuous secondary outcome variables:

The concentrations of soluble mediators (at least 50 such mediators) are measured at each follow-up visit, so we will apply a linear mixed-effects model to compare the estimated slopes for the HCQ and placebo groups with respect to each mediator over the minimum 96-week follow-up period. Let Y_{ijk} denote the natural logarithm of the concentration of one of the soluble mediators at the kth time point (k = 0, 1, … , 8, corresponding to 0, 12, … , 96 weeks) of the jth patient (j = 1, 2, … , n_i) within the ith clinical site (i = 1, 2, 3, 4, 5). Then the statistical model is

$$Y_{ijk} = \alpha_i + x_{ijk}^T\beta + z_{ijk}^T\delta_{ij} + \varepsilon_{ijk}$$

where

- α_i is the intercept parameter for the ith clinical site
- x_{ijk} is a vector of regressors at the kth time point for the jth patient within the ith clinical site
- β is a fixed-effects parameter vector
- z_{ijk} is a vector of regressors for the random-effects parameter vector at the kth time point for the jth patient within the ith clinical site
- δ_{ij} is a random-effects parameter vector for the jth patient within the ith clinical site, and we assume that the δ_{ij}'s are independent and identically distributed according to a multivariate normal distribution with a null mean vector and an unstructured, positive-definite, variance-covariance matrix Δ
- ε_{ijk} is a random error term at the kth time point for the jth patient within the ith clinical site, and we assume that the ε_{ijk}'s are independent and identically distributed according to a normal distribution with a null mean and variance σ^2

Safety and tolerability data will be summarized by treatment group, with descriptive statistics, and tested for association with treatment by Chi square or Fisher's exact test, as appropriate.

All safety analyses will be conducted in the safety population; all safety variables will be listed. Incidence and severity of treatment emergent AEs and SAEs and their relationship to treatment

will be summarized. Early discontinuation of study drug treatment will be presented and will include the reasons for and timing of such discontinuations. Abnormal physical examinations will be listed. Concomitant medications will be summarized. Laboratory evaluations will be summarized as observed values and changes from Baseline; shifts with respect to the laboratory reference range will be summarized. Vital signs will be summarized as observed values and changes from baseline.

Adverse event rates will be coded by body system and MedDra classification term. Adverse events will be tabulated by treatment group and will include the number of patients for whom the event occurred, the rate of occurrence, and the severity and relationship to study drug.

15.5 Interim Analysis

No interim efficacy analysis is planned for this study. Interim safety analyses will be performed as outlined in Section 14.

15.6 Sample Size and Randomization

The target sample size for this trial is 240 patients (120 randomized to each of the placebo and HCQ groups), which was calculated in the following manner. For the primary outcome variable of the SLICC classification criteria count, it is anticipated that the probability of the SLICC score progressing at least one stage during the 96-week follow-up period is 0.4 for a placebo patient, based on a composite of the pilot data and the report by Wieczorek et al[40]. The assumption is that HCQ will be deemed clinically effective if it can reduce this probability of progression to 0.2. The sample size must be large enough such that a two-sided, 0.05 significance level test, based on the statistical analysis for the primary outcome described above, can detect this clinically meaningful effect with 90% statistical power while accounting for a 25% loss to follow-up. Because the planned statistical analysis for the primary outcome variable is rather complex, a closed-form expression for the sample size calculation is not possible. Therefore, a computer study was performed with 1,000 simulated data sets such that, on the average, one-half of the placebo patients and one-fourth of the HCQ patients progressed at least one stage with respect to the SLIC score over a 96-week period. The null hypothesis of equal placebo and HCQ slopes was rejected in 901 of the 1,000 simulated data sets (90.1% statistical power) when the sample size was 240 subjects with a 25% withdrawal rate per data set.

With respect to the secondary outcome variable of the time to progression from ILE to clinical SLE, it is anticipated that a placebo patient has a 0.30 probability of such an occurrence during the 24-month follow-up period[40]. With the given sample size of 192 patients, there is 80% statistical power with a two-sided, 0.05 significance level test to detect a probability of 0.12 of progression to clinical SLE for an HCQ patient. This effect size in terms of a reduction in the probability from 0.30 to 0.12 corresponds to detecting the number need to treat (NNT) equaling 6. For the secondary outcome variable of the change in each of the 52 soluble mediators over the 96-week follow-up period with a sample size of 240 patients, there is 80% statistical power with a two-sided, 0.05 significance level, test (unadjusted for multiplicity) to detect a difference of 0.42 standard deviation units between the placebo and HCQ groups. This calculation is based on unadjusted analyses, however, so the actual effect size will be slightly larger than 0.42 standard deviation units when we apply the Hochberg step-down procedure. A conservative

approximation to the impact of the Hochberg procedure, based on a Bonferroni correction, is that the effect size will be 0.575 to 0.65 standard deviation units for each of the proteomic analytes.

Participant randomization occurs at Visit 3 and will be stratified by study site and SLICC score (seven sites, SLICC score of 2 or 3) with varying permutation block sizes to minimize the chance of inadvertent un-masking of group assignment.

16 DATA COLLECTION, RETENTION, AND MONITORING

16.1 Data Collection Instruments

The Investigator will prepare and maintain adequate and accurate source documents designed to record all observations and other pertinent data for each subject treated with the study drug.

Study personnel at each site will enter data from source documents corresponding to a subject's visit into the protocol-specific electronic Case Report Form (eCRF) when the information corresponding to that visit is available. Subjects will not be identified by name in the study database or on any study documents to be collected by the Sponsor (or designee), but will be identified by a site number, subject number and initials.

If a correction is required for an eCRF, the time and date stamps track the person entering or updating eCRF data and creates an electronic audit trail.

The Investigator is responsible for all information collected on subjects enrolled in this study. All data collected during the course of this study must be reviewed and verified for completeness and accuracy by the Investigator. A copy of the source documents and draft CRFs will remain at the Investigator's site at the completion of the study.

16.2 Data Management Procedures

The data will be entered into a validated REDCap database maintained at the Penn State College of Medicine Data Management Unit. The Data Management Unit will be responsible for data processing, in accordance with procedural documentation. Database lock will occur once quality assurance procedures have been completed.

All procedures for the handling and analysis of data will be conducted using good computing practices meeting FDA guidelines for the handling and analysis of data for clinical trials.

16.3 Data Quality Control and Reporting

After data have been entered into the study database, a system of computerized data validation checks will be implemented and applied to the database on a regular basis. Queries are entered, tracked, and resolved through the REDCap system directly. During study monitoring, a random sample of source documents will be compared to the eCRF data to ensure accuracy. The study database will be updated in accordance with the resolved queries. All changes to the study database will be documented.

16.4 Archival of Data

The database is safeguarded against unauthorized access by established security procedures; appropriate backup copies of the database and related software files will be

maintained. Databases are backed up by the database administrator in conjunction with any updates or changes to the database.

At critical junctures of the protocol (e.g., production of interim reports and final reports), data for analysis is locked and cleaned per established procedures.

16.5 Availability and Retention of Investigational Records

The Investigator must make study data accessible to the monitor, other authorized representatives of the Sponsor (or designee), IRB/IEC, and Regulatory Agency (e.g., FDA) inspectors upon request. A file for each subject must be maintained that includes the signed Informed Consent, HIPAA Authorization and Assent Form and copies of all source documentation related to that subject. The Investigator must ensure the reliability and availability of source documents from which the information on the CRF was derived.

All study documents (patient files, signed informed consent forms, copies of CRFs, Study File Notebook, etc.) must be kept secured for a period of three years following the conclusion of the study. There may be other circumstances for which the Sponsor is required to maintain study records and, therefore, the Sponsor should be contacted prior to removing study records for any reason.

16.6 Monitoring

Monitoring visits will be conducted by representatives of the Sponsor according to the U.S. CFR Title 21 Parts 50, 56, and 312 and ICH Guidelines for GCP (E6). By signing this protocol, the Investigator grants permission to the Sponsor (or designee), and appropriate regulatory authorities to conduct on-site monitoring and/or auditing of all appropriate study documentation.

Protocol compliance will be monitored by the staff of the Data Center for this trial that will be supervised by Dr. Duanping Liao in the School of Public Health, Penn State College of Medicine. This group has experience in the oversight of multi-center trials both in person and remotely. Personnel from the Data Center will routinely check each eCRF for validity and consistency and immediately ask research coordinators to resolve any discrepancies. This will include reviewing subject eligibility at entry and throughout the trial. Personnel from the Data Center will also make two in-person trips to each site to review source documents, investigational drug control, and compliance with local regulatory authorities. Any non-compliance by site investigators such as failure to resolve eCRF queries, failure to follow protocol procedures, or recruitment of ineligible subjects will be reported immediately to the Sponsor.

16.7 Subject Confidentiality

In order to maintain subject confidentiality, only a site number, subject number and subject initials will identify all study subjects on CRFs and other documentation submitted to the Sponsor. Additional subject confidentiality issues (if applicable) are covered in the NIH sub-award contract.

Links between the study ID and subject identifiers will be maintained in secure files by the site PIs and only available to their local study personnel for the safe conduct of the study. De-identified study data will be sent to Data Coordinating Center established for this trial at the Hershey Medical Center for aggregate analysis.

Data will be transmitted to the Data Coordinating Center using eCRF on the REDCap implementation maintained by the Penn State Biostatistics, Epidemiology, and Research Design (BERD) team within the Penn State Clinical and Translational Science Institute (CTSI). The database is securely hosted at the Penn State Hershey Medical Center and College of Medicine data center as a central location for data processing and management. Standard operational protocols are in place for the security and backup of the REDCap database within the Hershey Medical Center's data center. Within the data center, network access is controlled through multiple layers of protection that, at a minimum, consists of unique username and password authentication. Strict password strength and 90-day aging policies are applied to all user accounts from operating system to custom-developed DPHS applications. All information transmitted across the Internet is secured via 2048 bit Secure Sockets Layer (SSL) and/or Virtual Private Network (VPN)/Internet Protocol Security (IPSec) encryption. The DPHS network is also protected from security threats by enterprise level firewall and intrusion detection systems at Hershey Medical Center/College of Medicine campus and PSU network levels.

17 ADMINISTRATIVE, ETHICAL, AND REPORTING CONSIDERATIONS

The study will be conducted according to the Declaration of Helsinki, Protection of Human Volunteers (21 CFR 50), Institutional Review Boards (21 CFR 56), and Obligations of Clinical Investigators (21 CFR 312).

To maintain confidentiality, all laboratory specimens, evaluation forms, reports and other records will be identified by a coded number and initials only. All study records will be kept in a locked file cabinet and code sheets linking a patient's name to a patient identification number will be stored separately in another locked file cabinet. Clinical information will not be released without written permission of the subject, except as necessary for monitoring by the FDA. The Investigator must also comply with all applicable privacy regulations (e.g., Health Insurance Portability and Accountability Act of 1996, EU Data Protection Directive 95/46/EC).

17.1 Multi-Site Research Administration

17.1.1 Leadership

Dr. Nancy J. Olsen (Penn State M.S. Hershey Medical Center) is the study Sponsor and Protocol Co-Chair for overall administration, including Operations, Fiscal Management, and the Data Coordinating Center.

Dr. David R. Karp (UT Southwestern Medical Center) is the Protocol Co-Chair for Regulatory Affairs including IRB compliance, training, and reporting.

In the event that either Co-Chair is unable to continue their leadership role, a second Co-Chair will be selected from the other Principal Investigators at the participating sites.

17.1.2 Communication Plan

Dr. Olsen will conduct monthly teleconferences with all site principal investigators to review study operations (recruitment, informed consent, treatment, and data collection) as well as discuss any problems. Urgent problems with regard to the conduct of the study or subject safety

will be communicated by immediate teleconference and/or email. Dr. Olsen will communicate the results of the DSMB meetings to each site and notify the sites immediately of unplanned study closure.

Dr. Karp will maintain a repository of all current study documents including the most recent versions of the protocol and consent forms, local IRB approval letters, and evidence of training in human subjects protection by all study staff. Once approved by Dr. Olsen, a copy of the current protocol (original or amended) will be sent to each site by Dr. Karp. Site investigators will sign the assurance page and return that to Dr. Karp for archiving. Dr. Karp will notify Dr. Olsen and the Data Coordinating Center when each site has provided evidence of IRB approval and training so that site initiation can be started.

Site Investigators and their staffs will be responsible for the timely reporting of adverse events and unanticipated problems to Dr. Karp and the Medical Monitor as outlined in Sections 11 and 13 above.

17.2 Protocol Amendments

Any amendment to the protocol will be written by the Sponsor. Protocol amendments cannot be implemented without prior written IRB/IEC approval except as necessary to eliminate immediate safety hazards to patients. A protocol amendment intended to eliminate an apparent immediate hazard to patients may be implemented immediately, provided the IRBs are notified within five working days.

17.3 Institutional Review Boards and Independent Ethics Committees

The protocol and consent form will be reviewed and approved by the IRB/IEC of each participating center prior to study initiation. Serious adverse Events regardless of causality will be reported to the IRB/IEC in accordance with the standard operating procedures and policies of the IRB/IEC, and the Investigator will keep the IRB/IEC informed as to the progress of the study. The Investigator will obtain assurance of IRB/IEC compliance with regulations.

Any documents that the IRB/IEC may need to fulfill its responsibilities (such as protocol, protocol amendments, Investigator's Brochure, consent forms, information concerning patient recruitment, payment or compensation procedures, or other pertinent information) will be submitted to the IRB/IEC. The IRB/IECs written unconditional approval of the study protocol and the informed consent form will be in the possession of the Investigator before the study is initiated. The IRB/IECs unconditional approval statement will be transmitted by the Investigator to the Sponsor or designee prior to the shipment of study supplies to the site. This approval must refer to the study by exact protocol title and number and should identify the documents reviewed and the date of review.

Protocol and/or informed consent modifications or changes may not be initiated without prior written IRB/IEC approval except when necessary to eliminate immediate hazards to the patients or when the change(s) involves only logistical or administrative aspects of the study. Such modifications will be submitted to the IRB/IEC and written verification that the modification was submitted and subsequently approved should be obtained.

The IRB/IEC must be informed of revisions to other documents originally submitted for review; serious and/or unexpected adverse events occurring during the study in accordance with the standard operating procedures and policies of the IRB; new information that may affect

adversely the safety of the patients of the conduct of the study; an annual update and/or request for re-approval; and when the study has been completed.

17.4 Informed Consent Form

Informed consent will be obtained in accordance with the Declaration of Helsinki, ICH GCP, US Code of Federal Regulations for Protection of Human Subjects (21 CFR 50.25[a,b], CFR 50.27, and CFR Part 56, Subpart A), the Health Insurance Portability and Accountability Act (HIPAA, if applicable), and local regulations.

The Investigator will prepare the informed consent form, assent and HIPAA authorization and provide the documents to the Sponsor or designee for approval prior to submission to the IRB/IEC. The consent form generated by the Investigator must be acceptable to the Sponsor and be approved by the IRB/IEC. The written consent document will embody the elements of informed consent as described in the International Conference on Harmonization and will also comply with local regulations. The Investigator will send an IRB/IEC-approved copy of the Informed Consent Form to the Sponsor (or designee) for the study file.

A properly executed, written, informed consent will be obtained from each subject prior to entering the subject into the trial. Information should be given in both oral and written form and subjects (or their legal representatives) must be given ample opportunity to inquire about details of the study. If appropriate and required by the local IRB/IEC, assent from the subject will also be obtained. If a subject is unable to sign the informed consent form (ICF) and the HIPAA authorization, a legal representative may sign for the subject. A copy of the signed consent form (and assent) will be given to the subject or legal representative of the subject and the original will be maintained with the subject’s records.

17.5 Publications

The preparation and submittal for publication of manuscripts containing the study results shall be in accordance with a process determined by mutual written agreement among the study Sponsor and participating institutions. The publication or presentation of any study results shall comply with all applicable privacy laws, including, but not limited to, the Health Insurance Portability and Accountability Act of 1996.

17.6 Investigator Responsibilities

By signing the Agreement of Investigator form, the Investigator agrees to:

1. Conduct the study in accordance with the protocol and only make changes after notifying the Sponsor (or designee), except when to protect the safety, rights or welfare of subjects.
2. Personally conduct or supervise the study (or investigation).
3. Ensure that the requirements relating to obtaining informed consent and IRB review and approval meet federal guidelines, as stated in § 21 CFR, parts 50 and 56.
4. Report to the Sponsor or designee any AEs that occur in the course of the study, in accordance with §21 CFR 312.64.
5. Ensure that all associates, colleagues and employees assisting in the conduct of the study are informed about their obligations in meeting the above commitments.

6. Maintain adequate and accurate records in accordance with §21 CFR 312.62 and to make those records available for inspection with the Sponsor (or designee).
7. Ensure that an IRB that complies with the requirements of §21 CFR part 56 will be responsible for initial and continuing review and approval of the clinical study.
8. Promptly report to the IRB and the Sponsor (or designee) all changes in the research activity and all unanticipated problems involving risks to subjects or others (to include amendments and IND safety reports).
9. Seek IRB approval before any changes are made in the research study, except when necessary to eliminate hazards to the patients/subjects.
10. Comply with all other requirements regarding the obligations of clinical investigators and all other pertinent requirements listed in § 21 CFR part 312.

18 REFERENCES

1. Sanofi-Aventis US, LLC. PLAQUENIL Prescribing Information. 2006.

2. McChesney EW. Animal toxicity and pharmacokinetics of hydroxychloroquine sulfate. Am J Med 1983;75:11-8.

3. Ziegler HK, Unanue ER. Decrease in macrophage antigen catabolism caused by ammonia and chloroquine is associated with inhibition of antigen presentation to T cells. Proc Natl Acad Sci U S A 1982;79:175-8.

4. Kalish RS, Koujak S. Minocycline inhibits antigen processing for presentation to human T cells: additive inhibition with chloroquine at therapeutic concentrations. Clin Immunol 2004;113:270-7.

5. Schultz KR, Nelson D, Bader S. Synergy between lysosomotropic amines and cyclosporin A on human T cell responses to an exogenous protein antigen, tetanus toxoid. Bone Marrow Transplant 1996;18:625-31.

6. Kuznik A, Bencina M, Svajger U, Jeras M, Rozman B, Jerala R. Mechanism of endosomal TLR inhibition by antimalarial drugs and imidazoquinolines. J Immunol 2011;186:4794-804.

7. Rönnblom L, Alm GV. Systemic lupus erythematosus and the type I interferon system. Arthritis Res Ther 2003;5:68-75.

8. Sacre K, Criswell LA, McCune JM. Hydroxychloroquine is associated with impaired interferon-alpha and tumor necrosis factor-alpha production by plasmacytoid dendritic cells in systemic lupus erythematosus. Arthritis Res Ther 2012;14:R155.

9. Goldman FD, Gilman AL, Hollenback C, Kato RM, Premack BA, Rawlings DJ. Hydroxychloroquine inhibits calcium signals in T cells: a new mechanism to explain its immunomodulatory properties. Blood 2000;95:3460-6.

10. Hugosson E, Björkman A, Troye-Blomberg M. Chloroquine enhances the number of IL-10 producing cells and the expression of B7-2 and ICAM-1 in in vitro-cultured PBMC. Scand J Immunol 2002;55:399-408.

11. A randomized study of the effect of withdrawing hydroxychloroquine sulfate in systemic lupus erythematosus. The Canadian Hydroxychloroquine Study Group. N Engl J Med 1991;324:150-4.

12. Pons-Estel GJ, Alarcon GS, McGwin G, Jr., et al. Protective effect of hydroxychloroquine on renal damage in patients with lupus nephritis: LXV, data from a multiethnic US cohort. Arthritis Rheum 2009;61:830-9.

13. James JA, Kim-Howard XR, Bruner BF, et al. Hydroxychloroquine sulfate treatment is associated with later onset of systemic lupus erythematosus. Lupus 2007;16:401-9.

14. Pons-Estel GJ, Alarcon GS, Gonzalez LA, et al. Possible protective effect of hydroxychloroquine on delaying the occurrence of integument damage in lupus: LXXI, data from a multiethnic cohort. Arthritis Care Res (Hoboken) 2010;62:393-400.

15. Ruiz-Irastorza G, Ramos-Casals M, Brito-Zeron P, Khamashta MA. Clinical efficacy and side effects of antimalarials in systemic lupus erythematosus: a systematic review. Ann Rheum Dis 2010;69:20-8.

16. Rainsford KD, Parke AL, Clifford-Rashotte M, Kean WF. Therapy and pharmacological properties of hydroxychloroquine and chloroquine in treatment of systemic lupus erythematosus, rheumatoid arthritis and related diseases. Inflammopharmacology 2015;23:231-69.

17. Marmor MF, Kellner U, Lai TY, Lyons JS, Mieler WF, American Academy of O. Revised recommendations on screening for chloroquine and hydroxychloroquine retinopathy. Ophthalmology 2011;118:415-22.

18. Melles RB, Marmor MF. The risk of toxic retinopathy in patients on long-term hydroxychloroquine therapy. JAMA Ophthalmol 2014;132:1453-60.

19. Rahman A, Isenberg DA. Systemic lupus erythematosus. N Engl J Med 2008;358:929-39.

20. Ortega LM, Schultz DR, Lenz O, Pardo V, Contreras GN. Review: Lupus nephritis: pathologic features, epidemiology and a guide to therapeutic decisions. Lupus 2010;19:557-74.

21. Asanuma Y, Oeser A, Shintani AK, et al. Premature coronary-artery atherosclerosis in systemic lupus erythematosus. N Engl J Med 2003;349:2407-15.

22. Olsen NJ, Li QZ, Quan J, Wang L, Mutwally A, Karp DR. Autoantibody profiling to follow evolution of lupus syndromes. Arthritis Res Ther 2012;14:R174.

23. Wandstrat AE, Carr-Johnson F, Branch V, et al. Autoantibody profiling to identify individuals at risk for systemic lupus erythematosus. J Autoimmun 2006;27:153-60.

24. Li QZ, Zhou J, Lian Y, et al. Interferon signature gene expression is correlated with autoantibody profiles in patients with incomplete lupus syndromes. Clin Exp Immunol 2010;159:281-91.

25. Deane KD, El-Gabalawy H. Pathogenesis and prevention of rheumatic disease: focus on preclinical RA and SLE. Nat Rev Rheumatol 2014;10:212-28.

26. Olsen NJ, Karp DR. Autoantibodies and SLE--the threshold for disease. Nat Rev Rheumatol 2014;10:181-6.

27. Wandstrat A, Carr-Johnson F, Branch V, et al. Autoantibody profiling to identify individuals at risk for systemic lupus erythematosus. J Autoimmun 2006;27:153-60.

28. Pisetsky DS. Antinuclear antibodies in healthy people: the tip of autoimmunity's iceberg? Arthritis Res Ther 2011;13:109.

29. Somers EC, Marder W, Cagnoli P, et al. Population-based incidence and prevalence of systemic lupus erythematosus: The Michigan Lupus Epidemiology & Surveillance (MILES) Program. Arthritis Rheum 2013.

30. van de Stadt LA, Witte BI, Bos WH, van Schaardenburg D. A prediction rule for the development of arthritis in seropositive arthralgia patients. Ann Rheum Dis 2013;72:1920-6.

31. Greer JM, Panush RS. Incomplete lupus erythematosus. Arch Intern Med 1989;149:2473-6.

32. Li Q, Zhou J, Wandstrat A, et al. Protein array autoantibody profiles for insights into systemic lupus erythematosus and incomplete lupus syndromes. Clin Exp Immunol 2007;147:60-70.

33. Li Q, Zhou J, Lian Y, et al. Interferon signature gene expression is correlated with autoantibody profiles in patients with incomplete lupus syndromes. Clin Exp Immunol 2010;159:281-91.

34. Li QZ, Karp DR, Quan J, et al. Risk factors for ANA positivity in healthy persons. Arthritis Res Ther 2011;13:R38.

35. Touma Z, Urowitz MB, Ibanez D, Gladman DD. SLEDAI-2K 10 days versus SLEDAI-2K 30 days in a longitudinal evaluation. Lupus 2011;20:67-70.

36. Agresti A. Analysis of Ordinal Categorical Data, 2nd Edition: Wiley; 2010.

37. Peters SP, Kunselman SJ, Icitovic N, et al. Tiotropium bromide step-up therapy for adults with uncontrolled asthma. N Engl J Med 2010;363:1715-26.

38. Vonesh EF, Greene T, Schluchter MD. Shared parameter models for the joint analysis of longitudinal data and event times. Stat Med 2006;25:143-63.

39. Prentice RL, Gloeckler LA. Regression analysis of grouped survival data with application to breast cancer data. Biometrics 1978;34:57-67.

40. Wieczorek IT, Propert KJ, Okawa J, Werth VP. Systemic symptoms in the progression of cutaneous to systemic lupus erythematosus. JAMA Dermatol 2014;150:291-6.

APPENDIX 1 – SCHEDULE OF STUDY VISITS

**Visit number**	**1**	**2**	**3**	**4**	**5**	**6**	**7**	**8**	**9**	**10**	**11**	**NA**	**12**
**Visit description**[a]	SCRN	EYE	BSLN	F/U	F/U	F/U	F/U	F/U	F/U	F/U	EOS	US/EW	EYE
**Visit Week**	**0**	**0-5**	**0**-5	**16**[b]	**28**[b]	**40**[b]	**52**[b]	**64**[b]	**76**[b]	**88**[b]	**100**[b]		**100-104**
Informed consent	X												
Eligibility criteria	X		X										
Randomization			X										
Med History	X												
Demographics	X												
Vital signs	X		X	X	X	X	X	X	X	X	X	X	
Physical exam	X		X	X	X	X	X	X	X	X	X	X	
ANA by IFA and anti-CCP3.1	X												
Lab assessments	X		X	X	X	X	X	X	X	X	X	X[c]	
Urine pregnancy test	X						X				X		
Urinalysis	X		X	X	X	X	X	X	X	X	X		
Biomarker samples			X	X	X	X	X	X	X	X	X	X[c]	
Adverse events			X	X	X	X	X	X	X	X	X	X	
Concomitant medications	X		X	X	X	X	X	X	X	X	X	X	
Ophthalmology exam		X											X
CSQ	X												
SLEDAI/CLASI			X	X	X	X	X	X	X	X	X	X[c]	
SLICC/ACR Classification Criteria	X		X	X	X	X	X	X	X	X	X	X[c]	
SLICC/ACR Damage Index			X				X				X	X[c]	
Patient Reported Outcomes			X	X	X	X	X	X	X	X	X	X[c]	
Study drug compliance				X	X	X	X	X	X	X	X	X[c]	
Dispense study medication			X	X	X	X	X	X	X	X			

[a]SCRN – Screening; EYE – Ophthalmology; F/U – Follow Up; EOS – End of Study; US/ET – Unscheduled Visit or Early Withdrawal

[b]±7 days

[c]Perform only clinical portions of classification criteria; other items only if participant is withdrawing from study

APPENDIX 2 - SYSTEMIC LUPUS INTERNATIONAL COLLABORATING CLINICS 2012 CRITERIA FOR THE CLASSIFICATION OF SYSTEMIC LUPUS ERYTHEMATOSUS[1]

Category[2,3]	Features (score one per category)
Clinical Criteria	
Acute cutaneous lupus	• Lupus malar rash (do not count if malar discoid)<br>• Bullous lupus<br>• Toxic epidermal necrolysis variant of SLE<br>• Maculopapular lupus rash<br>• Photosensitive lupus rash *in the absence of dermatomyositis,* OR sub-acute cutaneous lupus
Chronic cutaneous lupus	• Classic discoid rash<br>o Localized (above the neck)<br>o Generalized (above and below the neck)<br>• Hypertrophic (verrucous) lupus<br>• Lupus panniculitis (profundus)<br>• Mucosal lupus<br>• Lupus erythematosus tumidus<br>• Chilblain lupus<br>• Discoid lupus/lichen planus overlap
Oral ulcers	• Palate<br>o Buccal<br>o Tongue<br>• OR, nasal ulcers<br>*In the absence of other causes, such as vasculitis, Behçet's disease, infection (herpesvirus), inflammatory bowel disease, reactive arthritis, and acidic foods*
Non-scarring alopecia	Diffuse thinning or fragility (with visible broken hairs) *in the absence of other causes such as alopecia areata, drugs, iron deficiency, or androgenic alopecia*
Synovitis	Involving 2 or more joints, characterized by swelling or effusion,<br>OR, tenderness if 2 or more joints and at least 30 minutes of morning stiffness

<table>
<tr><td>Serositis</td><td>Typical pleurisy for more than one day
OR pleural effusion
OR pleural rub
Typical pericardial pain (pain with recumbency improved by sitting forwarded) for more than one day
OR pericardial effusion
OR pericardial rub
OR pericarditis by electrocardiography in the absence of other causes such as infection, uremia, or Dressler's pericarditis</td></tr>
<tr><td>Renal</td><td>Urine protein-to-creatinine ration (or 24-hour urine protein) representing 500 mg protein/24 hours
OR red blood cell casts
OR a renal biopsy compatible with SLE nephritis</td></tr>
<tr><td>Neurologic</td><td>Seizures
Psychosis
Mononeuritis multiplex in the absence of other known causes such as primary vasculitis
Myelitis
Peripheral or cranial neuropathy in the absence of other known causes primary vasculitis, infection, or diabetes mellitus
Acute confusional state in the absence of other known causes, including toxic/metabolic, uremia, drugs</td></tr>
<tr><td>Hemolytic anemia</td><td></td></tr>
<tr><td>Leukopenia</td><td>Less than 4,000 WBC per mm^3 at least once in the absence of other known causes, including Felty's syndrome, drugs, and portal hypertension
OR, less than 1,000 lymphocytes per mm^3 at least once in the absence of other known causes such as corticosteroids, drugs, and infection</td></tr>
<tr><td>Thrombocytopenia</td><td>Less than 100,000 platelets per mm^3 at least once in the absence of other known causes such as drugs, portal hypertension, and thrombotic thrombocytopenia</td></tr>
<tr><td colspan="2">Immunological Criteria</td></tr>
<tr><td>Anti-nuclear antibody</td><td>Level above the laboratory reference range</td></tr>
</table>

Anti-dsDNA antibody	Level above the laboratory reference range, or > 2-fold the reference range if tested by ELISA
Anti-Sm	Presence of antibody to Sm nuclear antigen
Anti-phospholipid antibody	• Positive test result for lupus anticoagulant<br>• False-positive test result for rapid plasma reagin<br>• Medium- or high-titer anti-cardiolipin antibody level (IgG, IgM, IgA)<br>• Positive test result for anti-β2-glycoprotein I (IgG, IgM, IgA)
Low complement	• Low C3<br>• Low C4<br>• Low CH50
Direct Coombs' test	*In the absence of hemolytic anemia*

[1]Petri, M, *et al*., Arthritis & Rheumatism, 2012: 64:2677-2686.

[2]According to these criteria, a person is classified as having systemic lupus erythematosus (SLE) if they have at least one relevant feature present in four or more categories. Multiple features in each category are not counted extra. The features do not need to be present simultaneously. At least one clinical and one immunological feature need to be present. In addition, a person is classified as having SLE if they have a renal biopsy compatible with lupus nephritis and either an ANA or anti-dsDNA antibody.

[3]For this study, the subjects are assessed for the total number of clinical and immunological criteria with at least one relevant feature present. In addition, the individual features will be assessed and recorded.

APPENDIX 3 - 1997 UPDATE OF THE 1982 AMERICAN COLLEGE OF RHEUMATOLOGY REVISED CRITERIA FOR CLASSIFICATION OF SYSTEMIC LUPUS ERYTHEMATOSUS[1]

Criterion	Definition
Malar rash	Fixed erythema, flat or raised, over the malar eminence, tending to spare the nasolabial fold
Discoid Rash	Erythematous raised patches with adherent keratotic scaling and follicular plugging; atrophic scarring may occur in older lesions
Photosensitive Rash	Skin rash as a result of unusual reaction to sunlight, by patient history or physician observation
Oral ulcers	Oral or nasopharyngeal ulceration, usually painless, observed by physician
Non-erosive arthritis	Involving 2 or more peripheral joints, characterized by tenderness, swelling, or effusion
Pleuritis or Pericarditis	• Pleuritis--convincing history of pleuritic pain or rubbing heard by a physician or evidence of pleural effusion<br>• OR, Pericarditis--documented by electrocardiogram or rub or evidence of pericardial effusion
Renal Disorder	• Persistent proteinuria > 0.5 grams per day or > than 3+ if quantitation not performed<br>• OR, Cellular casts--may be red cell, hemoglobin, granular, tubular, or mixed
Neurological Disorder	• Seizures--in the absence of offending drugs or known metabolic derangements; e.g., uremia, ketoacidosis, or electrolyte imbalance<br>• OR, Psychosis--in the absence of offending drugs or known metabolic derangements, e.g., uremia, ketoacidosis, or electrolyte imbalance
Hematological Disorder	• Hemolytic anemia with reticulocytosis<br>• OR, Leukopenia < 4,000/mm3 on ≥ 2 occasions<br>• OR, Lymphopenia < 1,500/ mm3 on ≥ 2 occasions<br>• Thrombocytopenia <100,000/ mm3 in the absence of offending drugs
Immunologic Disorder	• Anti-DNA: antibody to native DNA in abnormal titer<br>• OR, Anti-Sm: presence of antibody to Sm nuclear antigen<br>• OR, Positive finding of anti-phospholipid antibodies on:<br>o An abnormal serum level of IgG or IgM anti-cardiolipin antibodies,<br>o A positive test result for lupus anticoagulant using a standard method, or<br>o A false-positive test result for at least 6 months confirmed by Treponema pallidum immobilization or fluorescent treponemal antibody absorption test

Positive Antinuclear Antibody	Abnormal titer of antinuclear antibody by immunofluorescence or an equivalent assay at any point in time and in the absence of drugs

[1] Hochberg, M.C., *Arthritis Rheum,* 1997: 40:1725.

[2]According to these criteria, a person is classified as having systemic lupus erythematosus (SLE) if they fulfill four or more criteria. The criteria do not need to be present simultaneously.

[3]For this study, the subjects are assessed for the total number of criteria satisfied. In addition, the individual defining features will be assessed and recorded.
