# Supplementary figures and images for: A Randomized, Placebo‐Controlled Trial of Hydroxychloroquine in Incomplete Lupus
Source: Arthritis Rheumatol. 2025 Dec 12;78(4):870–9. doi: 10.1002/art.43391 (PMC13054461; doi:10.1002/art.43391)

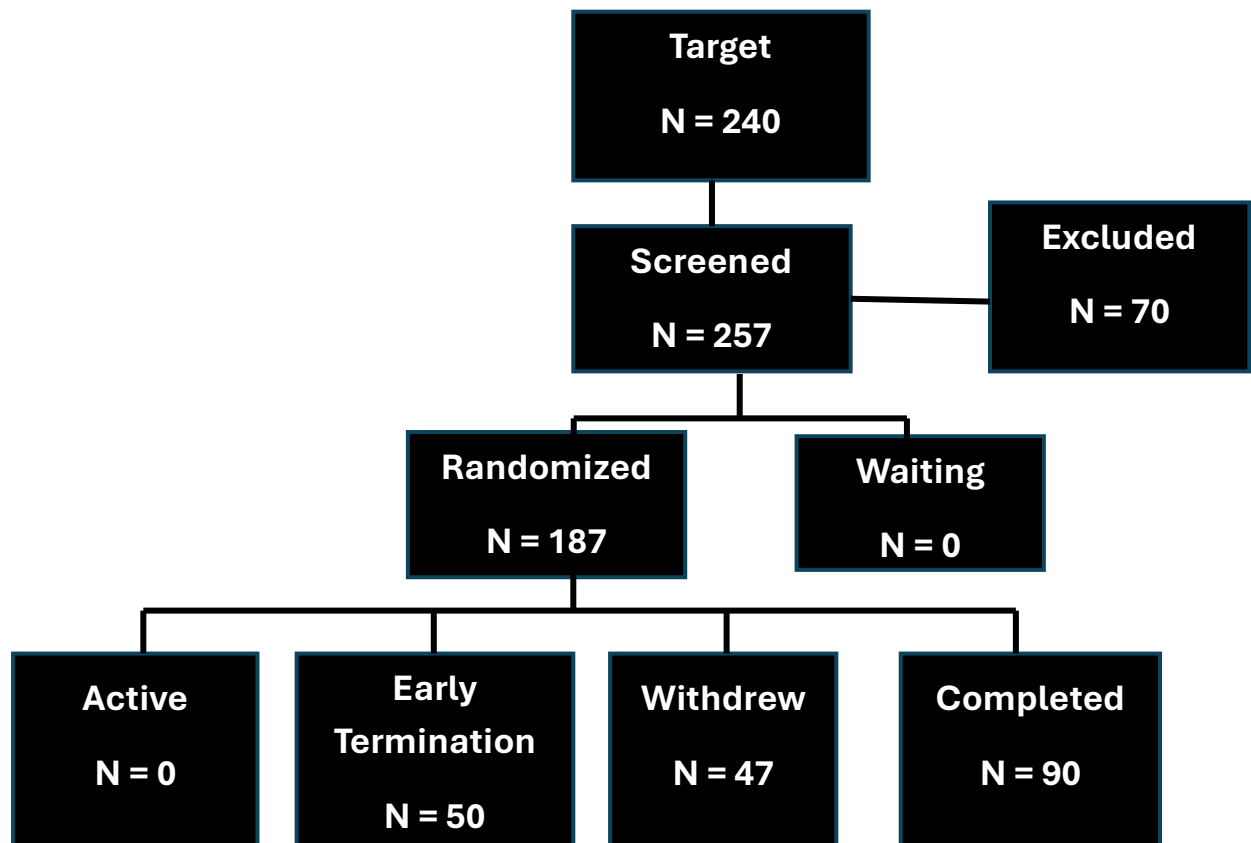

Supplement: Supplementary file 3 — Figure S1: [file ART-78-870-s004.pdf]
