# Supplementary material for: A Randomized, Placebo‐Controlled Trial of Hydroxychloroquine in Incomplete Lupus
Source: Arthritis Rheumatol. 2025 Dec 12;78(4):870–9. doi: 10.1002/art.43391 (PMC13054461; doi:10.1002/art.43391)
Supplement: Supplementary file 4 — Table S1: Final Study Termination Status [file ART-78-870-s002.docx]

| Supplementary Table 1  Final Study Termination Status | | | | |
| --- | --- | --- | --- | --- |
|  | | Hydroxychloroquine  N=92 | Placebo  N=88 | Total  N=180 |
| Discontinued treatment due to participant withdrawal | | 26 (28.3) | 21 (23.9) | 47 (26.1) |
| Reasons for early termination: | | N=23 | N=20 |  |
|  | Developed criteria for SLE | 11 (47.8) | 10 (50.0) | 21 (48.8) |
|  | Pregnancy | 2 (8.7) | 2 (10.0) | 4 (9.3) |
|  | Concomitant Medications | 1 (4.3) | 1 (5.0) | 2 (4.7) |
|  | Adverse Event | 1 (4.3) | 1 (5.0) | 2 (4.7) |
|  | Lost to followup | 5 (21.7) | 6 (30.0) | 11 (25.6) |
|  | Other reason | 2 (8.7) | 0 (0.0) | 2 (4.7) |
|  | Developed criteria for SLE and concomitant medications | 1 (4.3) | 0 (0.0) | 1 (2.3) |

* Number of participants (%)
